# Mendelian Randomization Studies of Myopia: Choosing the Right Summary Statistics

**DOI:** 10.1167/iovs.66.13.57

**Published:** 2025-10-31

**Authors:** Thu Nga Nguyen, Louise Terry, Jeremy A. Guggenheim

**Affiliations:** 1 School of Optometry and Vision Sciences, Cardiff University, Cardiff, United Kingdom

**Keywords:** mendelian randomization, myopia, glaucoma, case definition, educational attainment

## Abstract

**Purpose:**

To examine whether the choice of genome-wide association study (GWAS) summary statistics can yield invalid or misleading conclusions in Mendelian randomization (MR) studies of myopia.

**Methods:**

The relationships between (1) years of full-time education and myopia, and (2) myopia and primary open-angle glaucoma (POAG), were used as exemplar test cases. MR analyses were performed with nine different sets of summary statistics for myopia: seven from sources widely used in published MR studies, plus two newly derived sets (a GWAS for myopia in either 66,773 unrelated participants or 93,036 participants that included relatives).

**Results:**

Using the two newly derived sets of summary statistics from GWAS for myopia in unrelated and related samples, MR analyses demonstrated a positive causal relationship between education and myopia: odds ratio (OR) for myopia = 1.18, 95% confidence interval (CI) = 1.10 to 1.26 and OR = 1.16, 95% CI = 1.09 to 1.23 per additional year of education, respectively, and a positive relationship between myopia and POAG: OR = 1.11, 95% CI = 1.03 to 1.19 and OR = 1.12, 95% CI = 1.03 to 1.21, respectively. MR analyses performed using existing published GWAS summary statistics yielded inconsistent results, including MR estimates that suggested education protected against myopia and that myopia reduced the risk of POAG. Re-analysis of a selection of published MR studies of myopia confirmed that most published results were invalid.

**Conclusions:**

Care is required when designing MR analyses. Many published MR studies of myopia have reported misleading results.

Eye diseases have become increasingly prevalent, with detrimental effects on individuals, healthcare systems, and society.^1^^,^^2^ For example, myopia now affects approximately one third of the global population.^3^ Myopia increases the risk of sight-threatening eye disorders and results in an estimated productivity loss of around US$250 billion per year, globally.^4^^–^^9^ To address the burden of eye diseases, it is imperative to develop comprehensive preventive and management strategies by elucidating the risk factors and pathophysiological mechanisms underlying them.

Mendelian randomization (MR) is a statistical method for investigating the causal relationship between a potential exposure and an outcome, relying solely on observational data.^10^ MR takes advantage of the random assortment of alleles during gametogenesis to define groups that, on average, differ in their level of the exposure-of-interest: Individuals who inherit “high-risk” alleles tend to experience a higher level of the exposure in comparison to those who inherit “low-risk” alleles. The random assortment of alleles during meiosis (Mendel's second law) ensures that this genetically-conferred level of exposure is largely independent of socioeconomic and other environmental or lifestyle risk factors (but this can only be guaranteed in the absence of population stratification, assortative mating, and genetic nurture^11^). If strict, so-called “instrumental variable”, assumptions are met, then MR is robust to the presence of confounding factors and reverse causation. Numerous vision-related studies have used MR to assess the causal relationship between variables, such as years of education and myopia risk,^12^^,^^13^ myopia and primary open-angle glaucoma,^14^^,^^15^ and many more.^16^^–^^24^

One of the fundamental steps in conducting a two-sample MR analysis is to select appropriate data sources. Summary statistics from published genome-wide association studies (GWAS) that include regression coefficients for single nucleotide polymorphism (SNP)-exposure or SNP-outcome relationships are readily available for download from public databases, repositories, or research consortia websites. These sets of GWAS summary statistics enable MR analyses to be conducted in a short time with minimal resource requirements, which has led to a rapid expansion of MR publications in the research literature.^10^^,^^25^ Comprehensive guidelines on how to conduct or report MR studies have been published.^25^^,^^26^ However, these guidelines have focused little attention on the importance of choosing appropriate data sources for an MR analysis. Given the availability of multiple data repositories, researchers typically face the question of which set of summary statistics for an outcome or exposure should they choose? The various GWAS analyses for a trait-of-interest may have used different participant cohorts, case definitions, case ascertainment methods, or analysis methodologies. Each of these parameters will influence the estimated SNP-trait regression coefficients. This source of variability will feed through to influence the causal effect estimate of the MR analysis, potentially resulting in spurious findings that compromise the robustness and reliability of the research literature. The aim of the current work was to investigate if the choice of GWAS summary statistics can influence MR estimates. We focused on two exemplar test cases: (1) a two-sample MR analysis examining the role of educational attainment on myopia, and (2) a two-sample MR analysis examining the risk of primary open-angle glaucoma (POAG) in myopic vs. non-myopic individuals. Additionally, we re-analyzed selected published myopia MR studies to evaluate the reproducibility of earlier results. Our findings advocate for a more scientifically rigorous approach in selecting summary statistics for MR and interpreting the results obtained from such studies.

## Methods

### Part 1: Mendelian Randomization Analyses of the Two Exemplar Test Cases

#### Study Cohorts

We used publicly available summary statistics of GWAS from four sources: the UK Biobank, the FinnGen study, the Social Science Genetic Association Consortium, and an international consortium of glaucoma genetics researchers,^27^ as well as performing two new GWAS analyses for myopia in UK Biobank participants.

##### UK Biobank

Approximately 500,000 adults aged between 40 and 69 were recruited from 2006 to 2010.^28^ Participants visited one of 22 assessment centers across Great Britain for baseline and follow-up evaluations, during which their sociodemographic and clinical information was collected, including ophthalmic assessments completed by approximately 23% of participants. Genotype data were obtained using either the Biobank Axiom array (Affymetrix, High Wycombe, UK) or the BiLEVE Axiom array (Affymetrix), followed by imputation.

##### FinnGen

Approximately 500,000 individuals from Finland, averaging 53 years of age, consented to the use of their electronic health record (EHR) information and biological samples, as part of disease-based and population-based studies. The FinnGen cohort has been intentionally enriched with individuals suffering from various diseases. Phenotype data, which includes International Classification of Diseases (ICD)-10 codes, primarily derive from the Finnish national health registers, given Finland's comprehensive population-wide registry coverage. Genotype data were generated using the FinnGen ThermoFisher Axiom custom chip array (versions 1 and 2).^29^

##### Social Science Genetic Association Consortium

A total of 293,723 adults of European ancestry from different cohorts with educational attainment information were included in a large GWAS meta-analysis conducted by Okbay et al.^30^ The primary outcome variable, EduYears, was imputed based on the participants’ years of schooling, which was standardized according to the 1997 International Standard Classification of Education.

##### Glaucoma Genetics Consortium

A GWAS meta-analysis of POAG was conducted in 216,257 adults of European ancestry. POAG diagnosis was based on ICD9/ICD10 codes or other specific criteria, as listed in the Supplementary Data S1 of the original publication.^27^ Ethical approval for the study was obtained from each of the participating institutions. All participants provided informed consent.

#### GWAS Summary Statistics for Years of Education

We used summary statistics from the Okbay et al.^30^ GWAS for EduYears discovery analysis (*n* = 293,723) to avoid overlap with UK Biobank. The SNP-EduYears regression coefficients were reported in units of standard deviation in the original article; for the current analyses, these were converted to units of years of full-time education using a conversion factor of 1 *SD* = 3.6 years in school.^30^

#### GWAS Summary Statistics for POAG

Summary statistics for a GWAS meta-analysis of POAG across 21 independent samples of European ancestry (*n* = 16,677 POAG cases and *n* = 199,580 controls) were reported by Gharahkhani et al.^27^ UK Biobank participants (*n* = 1448 cases and *n* = 22,107) and FinnGen participants (*n* = 1824 cases and *n* = 93,036) were included in the GWAS meta-analysis of POAG of Gharahkhani et al.^27^. However, this small degree of sample overlap with UK Biobank and FinnGen was not expected to appreciably bias two-sample MR analyses.^31^

#### GWAS Summary Statistics for Myopia

Publicly available GWAS summary statistics for myopia in European-ancestry individuals were identified by searching the following public databases: GeneATLAS (Roslin Institute and Medical Research Council Human Genetics Unit, University of Edinburgh), GWASATLAS (VU University of Amsterdam), GWAS Catalog (National Human Genome Research Institute, European Molecular Biology Laboratory – European Bioinformatics Institute), IEU OpenGWAS project (UK Medical Research Council Integrative Epidemiology Unit [IEU], University of Bristol), and FinnGen. To facilitate the search, the terms “myopia,” “nearsightedness,” “shortsightedness,” and “refractive error” were used in the database queries. The results are presented in Table 1. The majority of published MR studies used GWAS summary statistics for analyses conducted in the UK Biobank cohort, making use of participants’ self-reported “Reason for glasses/contact lens: For short-sightedness (called ‘myopia’)” (UK Biobank data field 6147; response option 1) or Phecode 367.1 to define myopia. The remainder of the published studies used GWAS summary statistics from analyses conducted in FinnGen, which used the ICD code H52.1 to identify myopia cases. Ultimately, seven existing sets of GWAS summary statistics were included in a series of MR analyses. Details of each dataset are provided in Table 1 and Supplementary Note S1. For two of the seven selected sets of myopia summary statistics—those from the Neale lab and those from the IEU OpenGWAS—the GWAS regression coefficients were reported on the absolute “risk difference” scale rather than the logOR scale (the scale typically used in a case-control GWAS^32^). We suspected that previous MR studies may have not transformed the scale, despite reporting their MR results as odds ratios. To address this, we used both the original Neale lab and IEU OpenGWAS summary statistics and the data after transforming regression coefficients to the logOR scale. Details of the transformation calculation are provided in Supplementary Note S2.

**Table 1.** Publicly Available and Newly Derived GWAS Summary Statistics for Myopia

Database	ID/Authors	Study Cohort	Myopia Definition	Cases (N)	Controls (N)	Myopia Prevalence In GWAS Sample	Published MR Study Using the Summary Statistics
GWAS Catalog	GCST90044326	UK Biobank	Reason for glasses/contact lenses: For	36,623	419,031	8.0%	—
	Jiang et al.^43^		short-sightedness (UKB data field 6147)				
GWAS Catalog	GCST90435990	UK Biobank	Myopia (PheCode 367.1)	1257	406,530	0.3%	—
	Zhou et al.^34^						
IEU OpenGWAS	ukb-a-419	UK Biobank	Reason for glasses/contact lenses: For	26,943	308,757	8.0%	Xu et al.^58^
	(Neale Lab)		short-sightedness (UKB data field 6147)				
IEU OpenGWAS	ukb-b-6353	UK Biobank	Reason for glasses/contact lenses: For	37,362	423,174	8.1%	Deng et al.^59^
			short-sightedness (UKB data field 6147)				Jiang et al.^60^
							Li et al.^61^
							Liang et al.^62^
							Lv et al.^63^
							Mo et al.^64^
							Wei et al.^65^
							Xu et al.^58^
							Xu et al.^66^
							Zhang et al.^67^
							Zhu et al.^68^
FinnGen (R5)	finn-b-H7_MYOPIA	FinnGen	ICD Code (H52.1)	1640	210,931	0.8%	Li et al.^69^
							Wei et al.^70^
							Wei et al.^65^
							Xu et al.^58^
							Zhu et al.^68^
FinnGen (R9)	H7_MYOPIA	FinnGen	ICD Code (H52.1)	3534	361,237	1.0%	Deng et al.^59^
FinnGen (R10)	finn-b-H7_MYOPIA	FinnGen	ICD Code (H52.1)	4106	394,028	1.0%	Hui et al.^71^
Newly-derived	Current study	UK Biobank	SER in at least one eye ≤−0.5 diopter (including related individuals)	35,531	57,505	38.2%	—
Newly-derived	Current study	UK Biobank	SER in at least one eye ≤−0.5 diopter (only unrelated individuals)	25,804	40,969	38.6%	—

GWAS, Genome wide association study; ICD, International Classification of Diseases; SER, Spherical equivalent refractive error.

#### Newly Performed GWAS for Myopia in UK Biobank

Given the limitations of the currently available GWAS summary statistics for myopia (see below), we performed two new GWAS analyses for myopia in UK Biobank participants of European ancestry who had non-cycloplegic autorefraction measurement data and no other eye disorders. Details of the GWAS analyses can be found in Supplementary Note S3. Briefly, spherical equivalent refractive error (SER) was calculated as the autorefraction sphere power plus half the cylinder power. Participants were classified as myopia cases if the SER ≤ −0.50 diopters (D) in at least one eye. Controls were classified as individuals with SER > −0.50 D in both eyes. A GWAS for myopia in unrelated individuals (*n* = 25,804 cases; *n* = 40,969 controls) was conducted using PLINK2,^33^ while a GWAS for myopia that included related individuals (*n* = 35,531 cases; *n* = 57,505 controls) was conducted using SAIGE/GATE.^34^ Ethical approval for the UK Biobank study was obtained from the Northwest Multicentre Research Ethics Committee (Reference: 11/NW/0382). Participants provided informed consent and were free to withdraw from the study at any time. The research adhered to the tenets of the Declaration of Helsinki.

#### Selection of Instrumental Variables

Genetic variants were required to meet the following criteria to be included as instrumental variables (IVs) in our two exemplar testcases: (1) Independently associated with the respective exposure; (2) Available in the clumping reference panel and the outcome GWAS summary statistics; (3) F-statistic > 10 to reduce the risk of weak instrument bias.^35^ Full details of the instrumental variables selection for the two exposures, years of education and myopia, can be found in Supplementary Note S4. Ultimately, 62 genetic variants remained suitable for use as IVs for years of education (Supplementary Table S1). Table 3 shows the number of genetic variants used as IVs for myopia from each myopia summary statistic dataset.

Steiger filtering is a method for selecting genetic IVs designed to reduce the risk of reverse causation.^36^ To highlight the variability in results when using the same set of IVs, we present the results of MR analyses without Steiger filtering in the main manuscript. The corresponding analyses performed with Steiger filtering, which potentially yielded a different number of IVs for each analysis, produced broadly comparable results. The MR results obtained with Steiger filtering are provided in Supplementary Tables S3 and S5.

#### Mendelian Randomization Analysis

Full details of the MR analyses for the two exemplar testcases can be found in Supplementary Note S5, and code to reproduce the analyses is provided in Supplementary Notes S7-S9. The inverse-variance weighted (IVW) MR method was chosen as the primary analysis method.^37^ The following sensitivity analyses were performed to evaluate the robustness of the IVW-MR analysis against the assumption of no horizontal pleiotropy: MR-EGGER,^38^ weighted median MR,^39^ mode-based MR,^40^ and MR PRESSO.^41^

### Part 2: Re-Analyzing Published Myopia Mendelian Randomization Studies

To further illustrate the limitations of publicly available case-control myopia GWAS summary statistics, we re-analyzed selected published MR studies using our newly derived myopia GWAS summary statistics and the unit-of-measurement scale-transformed myopia GWAS datasets mentioned above. Full details of the literature search strategy and inclusion criteria to determine studies selected for re-analysis can be found in Supplementary Note S6. To ensure that any observed variations in the results were attributable to the choice of summary statistics, we adhered to the analysis plan outlined in the original articles. The only exception was the study conducted by Wei et al.,^42^ which adopted unusually liberal clumping criteria to select IVs. For the study by Wei et al.,^42^ we conducted our re-analyses using both their original methodology and a more conventional approach to assess the potential influence of analytical choices on the results.

## Results

A search of online GWAS repositories yielded 16 sets of myopia summary statistics publicly available for download. Twelve of the 16 sets of summary statistics were from the FinnGen study. We included the myopia summary statistics from FinnGen release 5, 9, and 10 in the current work, because these three sets have been used in published MR studies of myopia (Table 1). Two of the 16 sets of myopia summary statistics were from the IEU OpenGWAS database; we included these as they have been very widely used in published MR studies of myopia (Table 1). Finally, two of the 16 sets of myopia summary statistics were from the GWAS Catalog. Although these have not been used in published MR studies to our knowledge, we included them in the current analyses because their convenient availability would make them potentially suitable for an MR study of myopia.

To provide benchmark MR causal effect estimates to compare against those obtained using the publicly available myopia summary statistics, we performed two new GWAS analyses for myopia in samples of UK Biobank participants whose SER had been measured by autorefraction. These new sets of summary statistics have been made openly accessible.

### Mendelian Randomization Analysis of the Relationship Between Years of Education and Myopia

An IVW-MR analysis using summary statistics from our newly-performed GWAS for myopia in unrelated participants (25,804 cases and 40,969 controls) yielded an estimate for the causal effect of education on myopia of OR = 1.18 per year of education (95% CI, 1.10–1.26; *P* = 3.1e-06). An IVW-MR analysis using summary statistics from our newly-performed GWAS for myopia that included related individuals (35,531 cases and 57,505 controls) yielded a similar causal effect estimate of OR = 1.16 per year of education (95% CI, 1.09–1.23; *P* = 2.2e-06).

**Figure 1.**
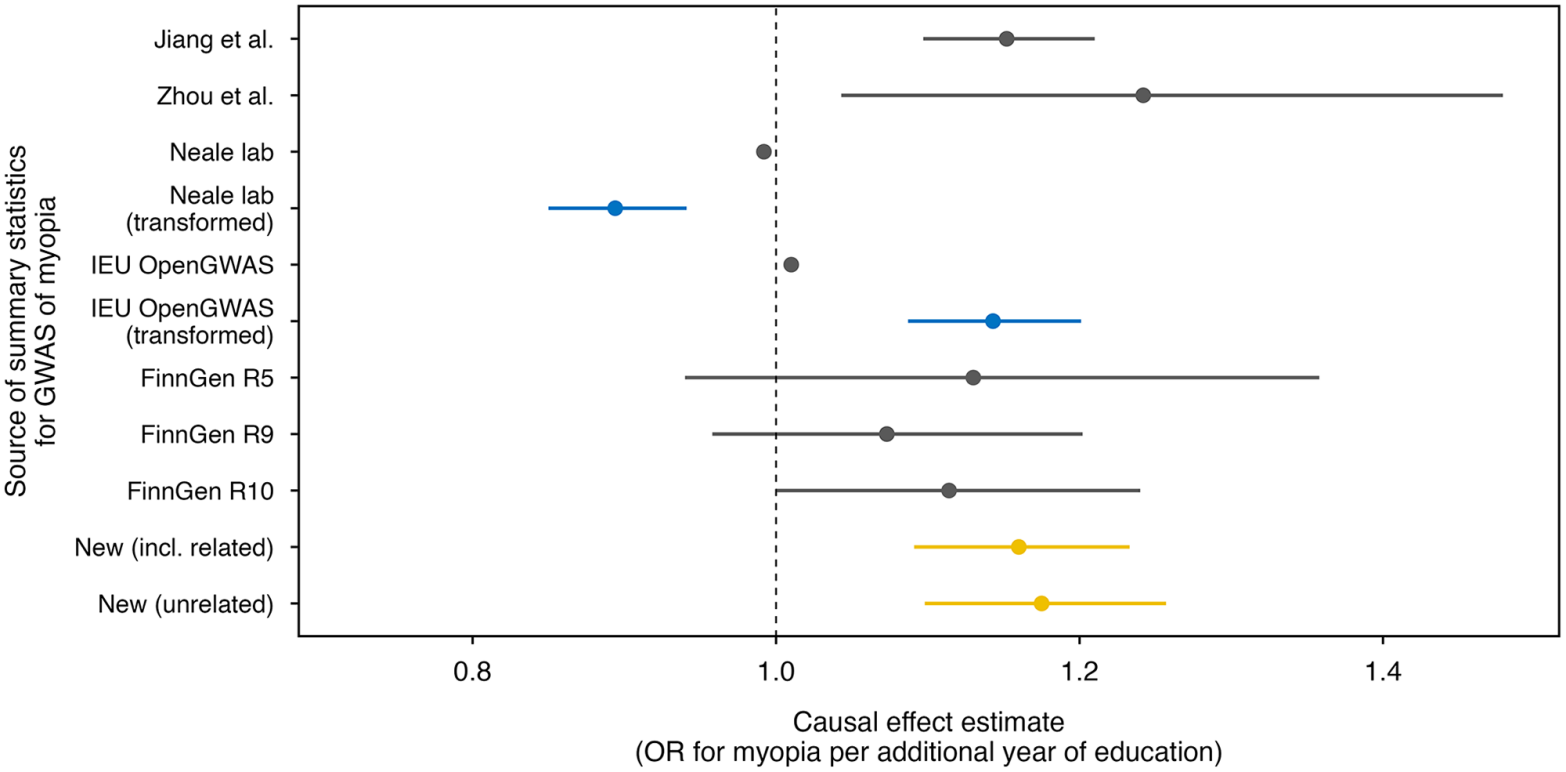
**Causal effect estimates from Mendelian randomization analyses of education as a risk factor for myopia obtained using different sets of GWAS summary statistics for myopia.** Each data point represents an analysis using a different set of GWAS summary statistics for myopia (*yellow* indicates analyses using newly derived GWAS summary statistics; *blue* represents analyses with transformed summary statistics, whereas *gray* denotes analyses using publicly available data datasets). *Error bars* indicate 95% CIs.

The results of the IVW-MR analyses using the seven sets of publicly available GWAS summary statistics for myopia are presented in Table 2; Figure 1, and Supplementary Figure S1. Complete results using the full range of MR methods are presented in Supplementary Table S2. These MR analyses yielded varying causal effect estimates for the relationship between education and myopia. As well as variability in effect size, the level of statistical significance of the causal effect varied widely, too. For instance, the MR analyses using the original and transformed versions of the GWAS summary statistics ukb-a-419 from the Neale lab repository suggested a highly significant *negative* causal relationship between years spent in full-time education and myopia status, albeit with different magnitudes (using the original dataset, OR = 0.99 per year of education; 95% CI, 0.988–0.996; *P* = 1.7e-05; using the transformed dataset, OR = 0.89 per year of education; 95% CI, 0.85–0.94; *P* = 1.5e-05), implying that additional education was protective against myopia. In contrast, analyses based on two versions of dataset ukb-b-6353 (original and transformed) from the IEU OpenGWAS project and the two datasets from the GWAS Catalog suggested evidence of a positive causal effect, with OR ranging from a minimal increase (OR = 1.01 per year of education in the original ukb-b-6353 dataset) to a more pronounced association (OR = 1.24). Meanwhile, MR analyses using myopia GWAS summary statistics from FinnGen releases R5, R9, and R10 failed to discern any causal effect of schooling years on myopia risk (all 95% CIs included the null value).

**Table 2.** Causal Effect of Years of Education on the Risk of Myopia Using Different Sets of Summary Statistics

GWAS for Myopia Summary Statistics	OR	95% CI	Effect^*^	SE	*P* Value
GCST90044326 (Jiang et al.^43^)	1.152	1.097–1.210	0.141	0.025	1.63e-08
GCST90435990 (Zhou et al.^34^)	1.242	1.043–1.479	0.217	0.089	1.49e-02
ukb-a-419 (Neale lab)	0.992	0.988–0.996	−0.008	0.002	1.70e-05
ukb-a-419_transformed^†^ (Neale lab)	0.894	0.850–0.941	−0.112	0.026	1.51e-05
ukb-b-6353 (IEU OpenGWAS)	1.010	1.006–1.014	0.010	0.002	1.74e-07
ukb-b-6353_transformed^†^ (IEU OpenGWAS)	1.143	1.087–1.201	0.133	0.025	1.63e-07
FinnGen (R5)	1.130	0.940–1.358	0.122	0.094	1.94e-01
FinnGen (R9)	1.073	0.958–1.202	0.070	0.058	2.24e-01
FinnGen (R10)	1.114	1.000–1.240	0.108	0.055	4.92e-02
Newly derived (including related individuals)	1.160	1.091–1.233	0.148	0.031	2.15e-06
Newly derived (unrelated individuals only)	1.175	1.098–1.257	0.161	0.035	3.11e-06

CI, Confidence interval; OR, Odds ratio; SE, Standard error.

Results were obtained using the IVW-MR method.

* Estimated effect in units of log odds ratio for myopia per additional year spent in education.

† Effect size of each genetic variant in the ukb-b-6353 and ukb-a-419 summary statistics files was transformed from an absolute risk difference scale to a log odds ratio scale according to the MRC IEU UK Biobank GWAS pipeline, version 2, 18/01/2019.

### Mendelian Randomization Analysis of the Relationship Between Myopia and POAG

An IVW-MR analysis using summary statistics from the newly-performed GWAS for myopia in unrelated participants (25,804 myopia cases and 40,969 controls) yielded an estimate for the risk of POAG in myopic vs. non-myopic individuals of OR = 1.11 (95% CI, 1.03–1.19; *P* = 6.5e-03). An IVW-MR analysis using summary statistics from the newly-performed GWAS for myopia that included relatives (35,531 myopia cases and 57,505 controls) yielded an estimate for the risk of POAG in myopic vs. non-myopic individuals of OR = 1.12 (95% CI, 1.03–1.21, *P* = 5.7e-03).

**Figure 2.**
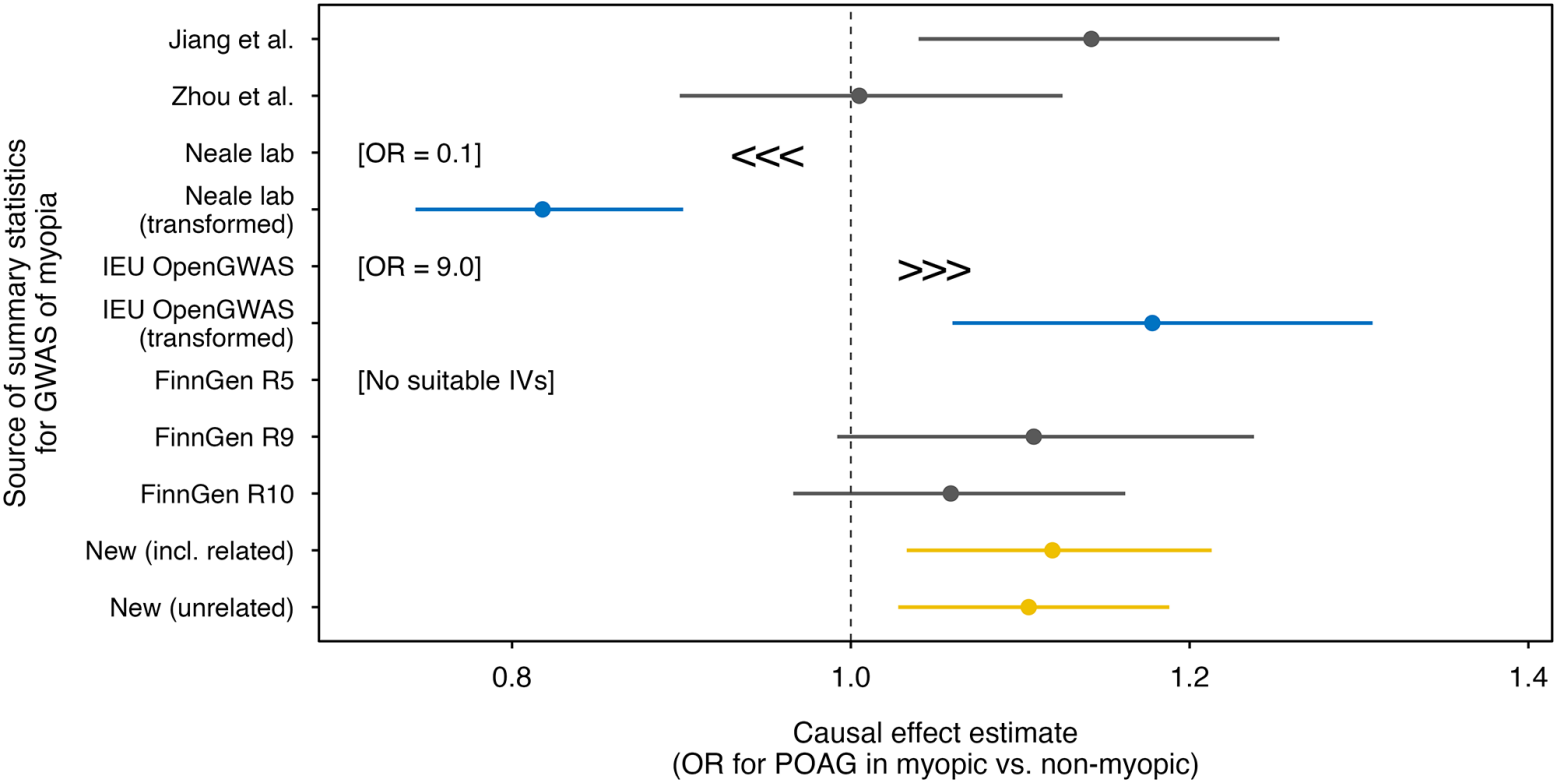
**Causal effect estimates from Mendelian randomization analyses of myopia as a risk factor for POAG obtained using different sets of GWAS summary statistics for myopia.** Each data point represents an analysis using a different set of GWAS summary statistics for myopia (*yellow* indicates analyses using newly derived GWAS summary statistics; *blue* represents analyses with transformed summary statistics, whereas *gray* denotes analyses using publicly available data datasets). *Error bars* indicate 95% CIs.

The results of the IVW-MR analyses using the publicly-available GWAS summary statistics are presented in Table 3; Figure 2, and Supplementary Figure S2. Complete results using the full range of MR methods are presented in Supplementary Table S4. Once again, MR analyses using different public sets of GWAS summary statistics for myopia yielded highly varied results. Strikingly, the MR analysis using the original version of myopia summary statistics ukb-a-419 from the Neale lab repository suggested myopia had a highly protective effect against POAG (OR = 0.07; 95% CI, 0.02–0.24; *P* = 4.1e-05). Equally striking was the estimated causal effect of myopia on POAG obtained with the original version of summary statistics ukb-b-6353 from the IEU OpenGWAS database, which suggested myopia significantly increased the risk of POAG several fold (OR = 8.98; 95% CI, 2.19–36.83, *P* = 2.3e-03). However, when utilizing the transformed versions of these two sets of summary statistics (ukb-a-419 and ukb-b-6353), the magnitude of the estimated causal relationship between myopia and POAG significantly diminished (using transformed ukb-a-419: OR = 0.82; 95% CI, 0.74–0.90; *P* = 4.1e-05; using transformed ukb-b-6353: OR = 1.18; 95% CI, 1.06–1.31; *P* = 2.3e-03). MR analyses using myopia summary statistics from Zhou et al.^34^ or FinnGen R9 and R10 suggested no evidence of a causal effect of myopia on POAG (all *P* > 0.05). Last, the myopia summary statistics of Jiang et al.^43^ yielded results similar to those from the newly derived and the transformed ukb-b-6353 summary statistics: OR = 1.14; 95% CI, 1.04–1.25; *P* = 5.6e-03. The number of IVs for myopia in these MR analyses also varied widely (Table 3). Indeed, for the FinnGen R5 myopia summary statistics, no GWAS variant met our *P* value threshold of *P* < 5.0e-08; hence, there were no IVs were available for an MR analysis using the FinnGen R5 summary statistics.

**Table 3.** Causal Effect of Myopia on the Risk of POAG Using Different Sets of Summary Statistics

GWAS for Myopia Summary Statistics	IVs	OR	95% CI	Effect^*^	SE	*P* Value
GCST90044326 (Jiang et al.^43^)	32	1.142	1.040–1.253	0.132	0.048	5.56E-03
GCST90435990 (Zhou et al.^34^)	1	1.005	0.899–1.125	0.005	0.057	9.26E-01
ukb-a-419 (Neale lab)	17	0.066	0.018–0.242	−2.718	0.663	4.14E-05
ukb-a-419_transformed^†^ (Neale lab)	17	0.818	0.743–0.901	−0.201	0.049	4.14E-05
ukb-b-6353 (IEU OpenGWAS)	36	8.977	2.188–36.829	2.195	0.720	2.31E-03
ukb-b-6353_transformed^†^ (IEU OpenGWAS)	36	1.178	1.060–1.308	0.164	0.054	2.31E-03
FinnGen (R5)	0	—	—	—	—	—
FinnGen (R9)	5	1.108	0.992–1.238	0.102	0.057	7.04E-02
FinnGen (R10)	8	1.059	0.966–1.162	0.058	0.047	2.22E-01
Newly derived (including related individuals)	48	1.119	1.033–1.213	0.113	0.041	5.72E-03
Newly derived (unrelated individuals only)	54	1.105	1.028–1.188	0.100	0.037	6.47E-03

Results were obtained using the IVW-MR method.

* Estimated effect in units of log odds ratio for POAG in myopic versus non-myopic individuals.

† Effect size of each genetic variant in the ukb-b-6353 and ukb-a-419 summary statistics files was transformed from an absolute risk difference scale to a log odds ratio scale according to the MRC IEU UK Biobank GWAS pipeline, version 2, 18/01/2019.

**Figure 3.**
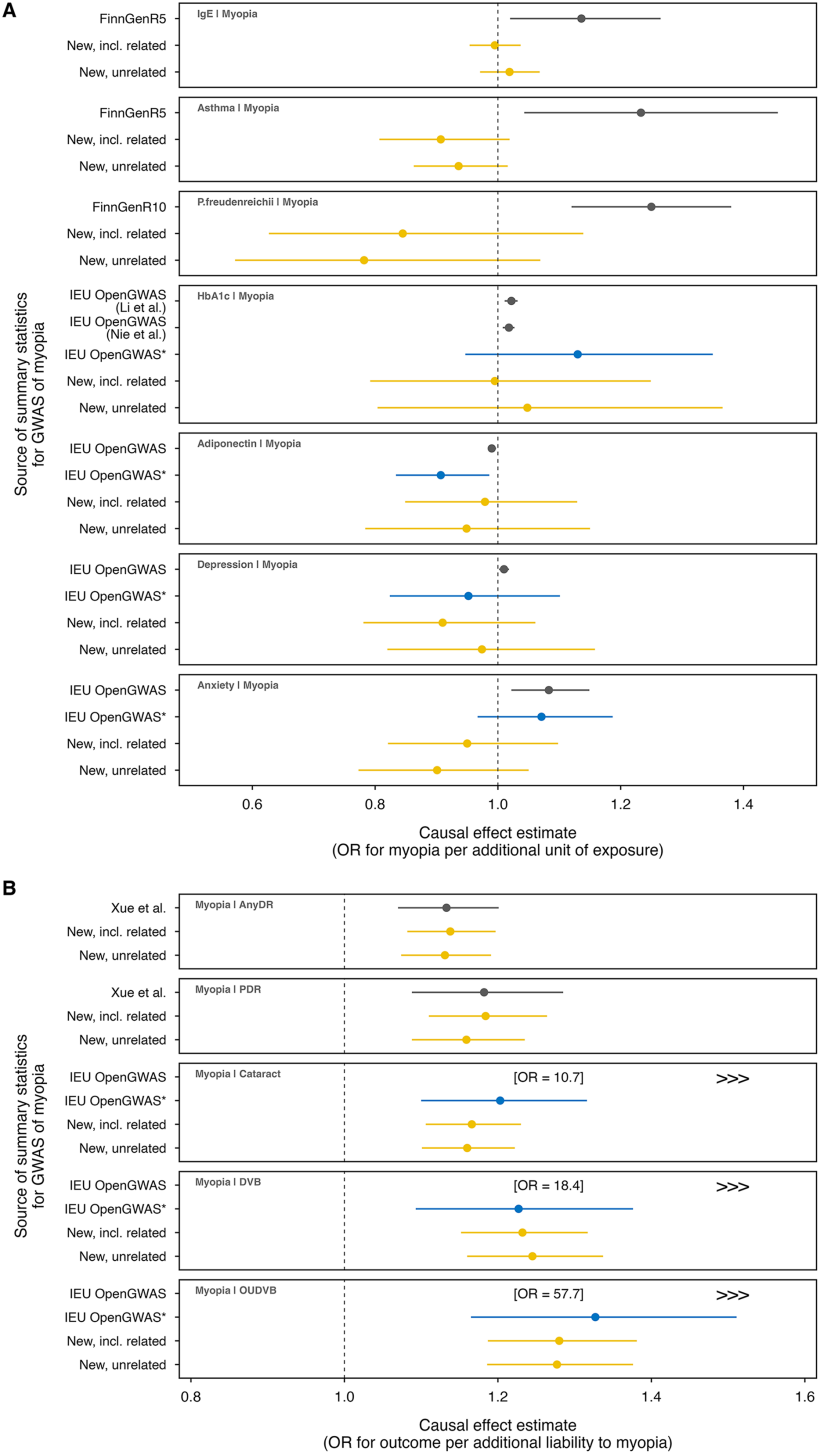
**Causal effect estimates from re-analyses of selected published Mendelian randomization studies of myopia using different sets of GWAS summary statistics.** Each data point represents an analysis using a different set of GWAS summary statistics for myopia (*yellow* indicates analyses using newly derived GWAS summary statistics; *blue* represents analysis with transformed IEU OpenGWAS summary statistics, while *gray* denotes analyses using publicly available data datasets). *Error bars* indicate 95% CIs. **(A)** Causal effect estimates from re-analyses where myopia is examined as the outcome. **(B)** Causal effect estimates from re-analyses where myopia is examined as the exposure. AnyDR, any diabetic retinopathy; PDR, proliferative diabetic retinopathy; DVB, disorders of vitreous body; OUDVB, other unspecified disorders of the vitreous body; IEU OpenGWAS*, IEU OpenGWAS (transformed).

### Prevalence of Myopia in the GWAS Samples

The prevalence of myopia in the GWAS samples used in published sets of summary statistics from UK Biobank ranged from 0.3%–8.1% (Table 1), whereas the true prevalence of myopia in UK Biobank participants is approximately 38%.^44^ The prevalence of myopia in the GWAS samples used in existing sets of summary statistics from FinnGen ranged from 0.8%–1.0%, whereas the prevalence of myopia in Finland is approximately 22%–30%.^45^^,^^46^

### Re-Analysis of Previously Published Mendelian Randomization Studies of Myopia

From 35 published MR studies that used GWAS summary statistics of myopia case-control status, we selected eight studies that met our criteria for re-analysis (Supplementary Note S6). The results of the re-analyses of the eight studies are presented in Table 4 and Figure 3 for the IVW-MR method, with the full results shown in Supplementary Tables S6–S12.

**Table 4.** Results of Re-Analyzing Selected Published Mendelian Randomization Studies of Myopia

			Original Study^§^			Re-Analysis With Transformed Ukb-b-6353			Re-Analysis 1 (GWAS for Myopia in Unrelated Participants)^§^			Re-Analysis 2 (GWAS for Myopia Including Relatives)^§^			
Study	Exposure	Outcome	IVs	OR	*P*	IVs	OR	*P*	IVs	OR	*P*	IVs	OR	*P*	Myopia GWAS Summary Statistics
Xu et al.^53^	Myopia	Any DR	26	1.133	1.91E-05	—	—	—	41	1.131	2.80E-06	36	1.138	4.27E-07	Xue et al.^54^
Xu et al.^53^	Myopia	Proliferative DR	26	1.182	8.31E-05	—	—	—	41	1.159	5.44E-06	36	1.184	3.38E-07	Xue et al.^54^
Liu et al.^72^	Myopia	Cataract (AR)	26	10.657	<0.001	28	1.203	5.27E-05	40	1.160	2.81E-08	35	1.166	1.67E-08	ukb-b-6353
Wei et al.^42^	Asthma^*^	Myopia	8	1.233	1.40E-02	—	—	—	5	0.936	1.14E-01	4	1.265	5.59E-01	FinnGen R5
Wei et al.^42^	Asthma^†^	Myopia	—	—	—	—	—	—	3	0.871	3.50E-02	3	0.900	9.83E-02	FinnGen R5
Wei et al.^42^	IgE^*^	Myopia	9	1.136	2.07E-02	—	—	—	19	1.019	4.49E-01	19	0.995	8.00E-01	FinnGen R5
Wei et al.^42^	IgE^†^	Myopia	—	—	—	—	—	—	1	1.263	5.45E-03	1	1.042	5.73E-01	FinnGen R5
Hui et al.^71^	*P.* *freudenreichii*	Myopia	Not reported	1.25	0.048^‡^	—	—	—	1	0.782	1.24E-01	1	0.845	2.70E-01	FinnGen R10
Xu et al.^73^	Anxiety	Myopia	10	1.083	8.0E-03	1	1.071	1.90E-01	1	0.901	1.83E-01	1	0.950	4.86E-01	ukb-b-6353
Xu et al.^73^	Depression	Myopia	21	1.010	1.60E-02	18	0.952	5.09E-01	19	0.974	7.69E-01	18	0.910	2.28E-01	ukb-b-6353
Nie et al.^74^	Adiponectin	Myopia	13	0.990	3.0E-03	12	0.907	2.21E-02	12	0.949	5.94E-01	13	0.979	7.72E-01	ukb-b-6353
Nie et al.^74^	HbA1c	Myopia	204	1.018	<0.001	10	1.130	1.76E-01	10	1.048	7.27E-01	10	0.995	9.64E-01	ukb-b-6353
Li et al.^61^	Adiponectin	Myopia	13	0.990	2.66E-03	12	0.907	2.21E-02	12	0.949	5.94E-01	13	0.979	7.72E-01	ukb-b-6353
Li et al.^61^	HbA1c	Myopia	213	1.022	3.06E-05	10	1.130	1.76E-01	10	1.048	7.27E-01	10	0.995	9.64E-01	ukb-b-6353
Xu et al.^66^	Myopia	DVB	28	18.387	<0.01	31	1.227	5.01E-04	41	1.245	1.54E-09	35	1.232	1.23E-09	ukb-b-6353
Xu et al.^66^	Myopia	OUDVB	28	57.729	<0.01	31	1.327	1.93E-05	40	1.277	1.24E-10	35	1.280	1.76E-10	ukb-b-6353

AR, age-related; DR, diabetic retinopathy; DVB, disorders of vitreous body; IVs, Instrumental variables; OUDVB, other unspecified disorders of the vitreous body.

Causal effect estimates reported in the original study were compared to those obtained in a re-analysis using our two sets of newly-derived GWAS summary statistics for myopia. Results are shown for the IVW-MR method; full results using a wider range of MR methods are presented in Supplementary Tables S5–S11.

* Re-analysis conducted adhering to the analysis plan in the original article (with a clumping threshold of *P* < 5E-06, *r*^2^ < 0.01, within an LD distance of ±1000kb).

† Re-analysis conducted according to conventional (stricter) approach (with a clumping threshold of *P* < 5E-08, *r*^2^ < 0.01, within LD distance of ±1000kb).

‡ False discovery rate adjusted *P* value.

§ Original: Results from the original articles; Re-analysis 1: Results from MR re-analysis using newly derived PLINK myopia GWAS summary statistics; Re-analysis 2: Results from MR re-analysis using newly derived SAIGE myopia GWAS summary statistics.

Only one re-analysis—an evaluation of the causal effect of myopia on any or proliferative diabetic retinopathy—produced findings that were consistent with the original article (for the outcome, “Any diabetic retinopathy”; original study: OR = 1.13; 95% CI, 1.07–1.20; *P =* 1.9e-05; re-analysis with GWAS for myopia in unrelated participants: OR = 1.13; 95% CI, 1.07–1.19; *P* = 2.8e-06; re-analysis with GWAS for myopia including related participants: OR = 1.14; 95% CI, 1.08–1.20, *P* = 4.3e-07; for the outcome, “Proliferative diabetic retinopathy”; original study: OR = 1.18; 95% CI, 1.09–1.29; *P =* 8.3e-05; re-analysis with GWAS for myopia including related participants: OR = 1.16; 95% CI, 1.09–1.24; *P* = 5.4e-06; re-analysis with GWAS for myopia including related participants: OR = 1.18; 95% CI, 1.11–1.26; *P* = 3.4e-07). The remaining seven re-analyses failed to validate the original claims made in the corresponding studies.

The discrepancy between the original study and our re-analyses varied from modest but statistically significant associations to non-significant results for the proposed causal effects of asthma, IgE, *Propionibacterium* *freudenreichii*, anxiety, depression, adiponectin, and HbA1c on myopia. Additionally, for the reported effects of myopia on age-related cataract, disorders of the vitreous body, and other unspecified disorders of the vitreous body, the re-analyses yielded markedly attenuated, albeit still statistically significant, causal estimates. As expected, when using the measurement scale-transformed myopia GWAS summary statistics, the new MR causal effect estimates were very different from the erroneous estimates reported in the published studies (Table 4).

## Discussion

This work revealed that MR analyses using different sets of publicly available GWAS summary statistics for myopia can yield contradictory findings. Our first exemplar test case examined the relationship between years of education and myopia. The two prior MR studies of this relationship both suggested that education is a causal risk factor for myopia.^12^^,^^13^^,^^47^^,^^48^ Here, we found that researchers performing an MR analysis of education and myopia would have obtained evidence of a positive causal relationship, a null relationship or even a negative causal association, depending on the choice of GWAS summary statistics. Our second exemplar test case examined the risk of POAG in myopic versus non-myopic individuals. Of the two prior MR studies examining this relationship, both reported a positive causal effect of myopia on the risk of POAG; in addition, one of the studies also reported a bidirectional causal relationship, namely, that POAG also increased the risk of myopia.^14^^,^^15^ Here, we found that researchers performing an MR analysis would have obtained evidence of a large protective effect of myopia on the risk of POAG (OR < 0.1), a large increased risk (OR ≈ 9.0), or a non-significant relationship, depending on the choice of summary statistics for myopia. As discussed in detail below, the reasons for these disparate findings either stem from poorly designed GWAS analyses in which myopia cases and controls were often misclassified or from the lack of attention by the researcher to the measurement scale of the GWAS summary statistics.

Myopia is a common refractive error. Among the middle and older-aged populations of Europe the prevalence of myopia is approximately 30.6%.^49^ However, the benign nature of myopia in most individuals has led to myopia being underreported in EHR.^50^^,^^51^ For example, the myopia prevalence in the FinnGen sample based on EHR information (ICD code H52.1) is approximately 1%, yet the true prevalence of myopia in Finland is 22%–30%.^45^^,^^46^ Work by Wittenborn and colleagues^51^ suggested that, as well as myopia, several other eye conditions were markedly underreported in an EHR system. Thus a GWAS for myopia in the FinnGen sample using EHR-based information would analyze a sample of participants with a case-control ratio of approximately 1:100; the cases would be bona fide myopia cases, but 20%–30% of the controls would be myopic individuals who were misclassified. When using MR to evaluate the relationship between education and myopia, these misclassification issues with the FinnGen myopia summary statistics led to loss of statistical power, even with an imbalance case-control GWAS adjusted method,^34^ such that the causal effect of education on myopia was difficult to distinguish from a null causal effect (Table 2; Fig. 1). Similarly, in the MR examining the relationship between myopia and POAG, the misclassification issues with the FinnGen myopia summary statistics led to few SNPs being available as instrumental variables, leading to limited statistical power to distinguish a causal effect from a null effect.

By contrast to the FinnGen study, the UK Biobank study performed direct assessments of refractive error using autorefraction and specifically asked participants if they were nearsighted. However, because the ophthalmic assessment component was introduced late in the UK Biobank recruitment process, only 23% of participants underwent the eye examinations and completed the ophthalmic questionnaire.^44^ When researchers from the study by Jiang et al.,^43^ the Neale lab, and the IEU OpenGWAS project classified UK Biobank participants as myopia cases or controls, the lack of ophthalmic assessment information was not taken into account; instead, the 385,000 participants who were not asked about their myopia status were all classified as non-myopic controls. Specifically, the GWAS for myopia performed by the Neale lab, IEU OpenGWAS project, and Jiang et al.^43^ used 27,000, 36,500, and 37,000 correctly classified cases, respectively, but 309,000, 419,000, and 423,000 controls, respectively, of whom about 38% were misclassified. On the other hand, the study by Zhou et al.^34^ used PheCode, a hierarchical phenotyping tool based on ICD codes, to identify myopia cases within the UK Biobank cohort. This approach, which also relied on the EHR system to define myopia, identified only 1257 myopic cases versus 406,000 controls—again with roughly 38% of controls misclassified—yielding even fewer cases than FinnGen's myopia GWAS summary statistics. Compared with the Neale lab, IEU OpenGWAS, and Jiang et al.^43^ studies, which used the same cohort for GWAS analyses, the Zhou et al.^34^ myopia GWAS exhibited more pronounced misclassification and an exceptionally high case-control ratio (approximately 1:300). This resulted in diminished statistical power for MR analyses that, even with adjustment for GWAS case-control imbalance,^34^ suggested a null MR causal effects for the myopia-POAG relationship (Table 3). Conversely, the somewhat less severe case-control misclassification in the remaining three UK Biobank myopia GWAS summary statistics (Jiang et al.^43^ and the transformed versions of ukb-a-419 and ukb-b-6353) produced broadly comparable effect magnitudes to the benchmark estimates in the two exemplar test cases, although the direction of the effects in the case of ukb-a-419 was opposite to that in the other two (as discussed below). This raises the question of what level of GWAS case-control misclassification can be tolerated before downstream MR analyses are significantly impacted?

An additional concern regarding the use of existing GWAS summary statistics for myopia, which specifically related to sources ukb-a-419 (Neale lab) and ukb-b-6353 (IEU OpenGWAS project), was misinterpretation of the regression coefficient unit. Of the sets of publicly available myopia summary statistics, most employed GWAS regression methods designed for analyzing binary traits; these methods output results in which the regression coefficient units are on the “logOR” measurement scale: for example, Jiang et al.^43^ used fastGWA-GLMM, whereas Zhou et al.^34^ and the FinnGen team^34^ used SAIGE. However, the Neale lab and IEU OpenGWAS project used linear regression for their GWAS analyses, with Hail and BOLT-LMM, respectively. Consequently, the GWAS regression (beta) coefficients from the Neale lab and IEU GWAS analyses were measured on the absolute “risk difference” scale, indicating the absolute change in the prevalence of myopia per copy of the effect allele. Failure to carefully consider the units of the measurement scale could yield misleading MR causal effect estimates and erroneous study conclusions. In the context of an MR analysis using a binary exposure with measurement units on the absolute risk difference scale, the causal effect estimate corresponds to the change in the outcome if the population prevalence of the trait-of-interest were increased by 1.0 unit (i.e., from 0% to 100% prevalence of the exposure); such a change would be extreme and may even necessitate rescaling of the MR estimates.^52^ The impact of not transforming GWAS regression coefficients from the absolute risk difference scale to the logOR scale was evident in our two exemplar test cases. For both the EduYears-myopia and myopia-POAG MR analyses, converting the regression coefficient units to the logOR scale changed the causal effect estimates from strikingly different, to roughly comparable, with the benchmark results, aside from the sign-reversal issue of the Neale lab summary statistics (Tables 2, 3; Figs. 1, 2).

Finally, as mentioned above, the GWAS summary statistics from the Neale lab had wrongly labeled risk and reference alleles, which led to MR causal effect estimates in the opposite direction to that expected, for example suggesting that additional education reduced the risk of myopia. We downloaded the Neale lab myopia summary statistics from the IEU OpenGWAS repository, since the original Neale lab GWAS repository no longer exists and because the IEU OpenGWAS repository is the most widely used source of myopia summary statistics for use in published MR studies (Table 1). We were able to confirm that a more recent release of myopia summary statistics by the Neale lab has the risk and effect alleles correctly labeled (however, the misclassification of controls and the misinterpretation of GWAS regression coefficient units remain as potential issues).

We further illustrated the limitations of some of the publicly available GWAS summary statistics for myopia through our re-analysis of specific published myopia MR studies. Only one out of eight re-analyses generated findings that were consistent with the original study. The study^53^ whose findings we did validate used myopia GWAS summary statistics from a prior UK Biobank analysis^54^ that defined myopic cases and controls based on SER measurements, as we did in our new GWAS analyses. The seven non-replicated re-analyses relied on myopia GWAS summary statistics from either the IEU OpenGWAS project (ukb-b-6353) or FinnGen R5/R10. Thus we suspect that the spurious findings reported in those studies are attributable to the shortcomings of these summary statistics, as discussed above.

In light of our findings, we offer recommendations for selecting summary statistics for an MR study. First, we suggest that researchers prioritize using GWAS summary statistics based on continuous traits whenever feasible (e.g., SER instead of myopia status) to circumvent the limitations of the case ascertainment methods discussed above. Continuous variables also simplify the calculation of key statistics such as *R*^2^ and the *F*-statistic^55^ and reduce the potential for misinterpretation of effect sizes. However, we acknowledge that GWAS summary statistics for highly relevant continuous traits may not always be readily available. In such instances, we recommend that researchers endeavor to identify GWAS summary statistics datasets that possess a substantial sample size and use phenotyping methods that minimize the potential for misclassification bias. This can be achieved by using ICD codes or EHRs in conditions that necessitate hospitalization, such as glaucoma or cataract. Alternatively, for conditions that are typically managed in primary care settings, researchers should strive to obtain GWAS summary statistics from samples in which the case prevalence closely approximates the population prevalence of the trait. When this is not feasible, researchers should acknowledge the limitations of their chosen summary statistics and explain their rationale to readers to ensure clear interpretation of their MR results.

Although our exemplar analyses were limited in scope, we acknowledge that several other crucial caveats of MR studies warrant attention. These include the absence of multiple testing corrections in some prior studies, high degrees of sample overlap in two-sample MR designs without steps to address its effects, the use of mixed-ancestry samples, overly permissive criteria for genetic instrument selection, lack of transparency in analytical plans, and variability in genetically predicted exposure levels across different life stages, among others. In addition, pioneers of the use of MR in epidemiology have cautioned that even well-conducted MR studies have limitations.^25^^,^^26^ Genetic variants identified in large scale GWAS analyses may have small effects and be at risk of introducing “weak instrument bias.”^56^ For a two-sample MR analysis, weak instrument bias will generally bias causal effect estimates toward the null. Alternatively, if the SNPs selected as instrumental variables have horizontally pleiotropic effects, then this can lead to either exaggerated or underestimated causal effect estimates.^57^ Although these additional issues fall outside the focus of our article, we emphasize that MR, although a powerful tool for causal inference, must be applied with methodological rigor and critical judgment.

## Conclusions

MR analyses performed using different sets of publicly available GWAS summary statistics for myopia can yield conflicting results. Indeed, the results of some previously published MR studies that have used these resources were shown to be invalid (Table 4). Although we investigated just two exemplar scenarios and re-analyzed eight MR studies of myopia, we suspect the issue of inappropriate binary trait GWAS summary statistics may extend to other ophthalmic diseases. We urge researchers to exercise caution when selecting GWAS summary statistics for an MR study: The adage “choose the GWAS study with the largest sample size” may not always hold.^10^ Specifically, researchers should prioritize GWAS summary statistics of continuous traits whenever feasible. If this is not possible, careful consideration should be given to the underlying population, case definitions, case ascertainment methods, and association analysis procedures when selecting binary trait summary statistics for MR. We have made our two newly derived sets of myopia summary statistics openly available and encourage researchers to use these for future MR studies of myopia.

## Supplementary Material

Supplement 1
