# Supplementary material for: Mendelian Randomization Studies of Myopia: Choosing the Right Summary Statistics
Source: Invest Ophthalmol Vis Sci. 2025 Oct 31;66(13):57. doi: 10.1167/iovs.66.13.57 (PMC12582192; doi:10.1167/iovs.66.13.57)
Supplement: Supplement 1 [file iovs-66-13-57_s001.docx]

**Mendelian Randomization Studies of Myopia: Choosing the right Summary Statistics**

**Supplementary Material**

Contents

[Supplementary Note S1. Existing publicly available GWAS summary statistics for myopia 3](#_Toc208669840)

[Supplementary Note S2. Transformation of the effect sizes and standard errors of IEU OpenGWAS and the Neale lab’s myopia GWAS summary statistics 5](#_Toc208669841)

[Supplementary Note S3. Newly-performed GWAS for myopia 6](#_Toc208669842)

[Supplementary Note S4. Selection of instrumental variables 7](#_Toc208669843)

[Supplementary Note S5. Mendelian randomization analysis of the two exemplar testcases 8](#_Toc208669844)

[Supplementary Note S6. Search strategy, inclusion criteria, and list of papers excluded for MR re-analysis 9](#_Toc208669845)

[Supplementary Note S7. Statistical code to reproduce the analyses. 11](#_Toc208669846)

[Supplementary Note S8. Code to reproduce R^2^, R^2^_l_, and F-statistic calculations 25](#_Toc208669847)

[Supplementary Note S9. Code to reproduce Steiger filtering 29](#_Toc208669848)

[Supplementary Table S1. Genetic variants (N=62) from the SSGAC GWAS for EduYears used as instrumental variables for MR. 31](#_Toc208669849)

[Supplementary Table S2. Full MR results for analyses examining the effect of EduYears on myopia without Steiger filtering. 33](#_Toc208669850)

[Supplementary Table S3. Full MR results for analyses examining the effect of EduYears on myopia with Steiger filtering. 35](#_Toc208669851)

[Supplementary Table S4. Full MR results for analyses examining the effect of myopia on POAG without Steiger filtering. 37](#_Toc208669852)

[Supplementary Table S5. Full MR results for analyses examining the effect of myopia on POAG with Steiger filtering. 39](#_Toc208669853)

[Supplementary Table S6. Full MR results for re-analyses examining the effect of myopia on any diabetic retinopathy (AnyDR) and proliferative diabetic retinopathy (PDR). 41](#_Toc208669854)

[Supplementary Table S7. Full MR results for re-analyses examining the effect of myopia on age-related cataract (ARC). 42](#_Toc208669855)

[Supplementary Table S8. Full MR results for re-analyses examining the effect of myopia on disorders of the vitreous body (vitrbodyglobe) and other unspecified disorders of the vitreous body (vitroth). 43](#_Toc208669856)

[Supplementary Table S9. Full MR results for re-analyses examining the effect of asthma and immunoglobulin E (IgE) on myopia. 44](#_Toc208669857)

[Supplementary Table S10. Full MR results for re-analyses examining the effect of *Propionibacterium Freudenreichii (P. freudenreichii)* on myopia. 45](#_Toc208669858)

[Supplementary Table S11. Full MR results for re-analyses examining the effect of anxiety and depression on myopia. 46](#_Toc208669859)

[Supplementary Table S12. Full MR results for re-analyses examining the effect of adiponectin and HbA1c on myopia. 47](#_Toc208669860)

[Figure S1. Graphs of SNP vs. EduYears and SNP vs. Myopia regression coefficients for the inverse variance-weighted Mendelian randomization analyses using different sets of myopia summary statistics. 48](#_Toc208669861)

[Figure S2. Graphs of SNP vs. Myopia and SNP vs. POAG regression coefficients for the inverse variance-weighted Mendelian randomization analyses using different sets of myopia summary statistics. 49](#_Toc208669862)

[Figure S3. The impact of misinterpretation of GWAS measurement scale on the SNPs used for the EduYears-Myopia MR analysis. 50](#_Toc208669863)

[Figure S4. The impact of misinterpretation of GWAS measurement scale on the SNPs used for the Myopia-POAG MR analysis. 51](#_Toc208669864)

[References 52](#_Toc208669865)

# Supplementary Note S1. Existing publicly available GWAS summary statistics for myopia

**GCST90435990***, Zhou et al*.^1^ This GWAS for myopia was reported in 2018 in an article presenting the Scalable and Accurate Implementation of Generalized mixed model (SAIGE) method. A total of 28,000,000 genetic markers were tested on a sample of 407,787 individuals from the UK Biobank using a generalized linear mixed model (GLMM)-based method. Myopia case-control status was defined using PheCode 367.1, which is based on hospital records. Since myopia is rarely listed in general medical records by the UK National Health Service, this resulted in severe misclassification bias (myopia prevalence inferred to be 0.3% rather than the true prevalence of ~38%).

**GCST90044326***, Jiang et al.*^2^ This GWAS for myopia was reported in 2021 as part of an article presenting the fastGWA-GLMM method. Using a GLMM-based method, 11,842,647 SNPs were tested in a sample of 455,654 individuals from the UK Biobank. Myopia was defined based on UKB data field 6147 (Reason for glasses/contact lenses: For short-sightedness). However, Jiang et al. did not take into account that only 25% of UK Biobank participants were asked this question. Participants who were not asked the question were categorized as controls, which resulted in severe misclassification bias (myopia prevalence inferred to be 8% rather than the true prevalence of ~38%).

**ukb-b-6353**. This GWAS for myopia was conducted between 2017 and 2018 on a sample of 460,536 individuals from the UK Biobank by the MRC-IEU Consortium.^3^ A total of 9,851,867 SNPs were tested using the linear mixed model association method implemented in BOLT-LMM (version 2.3). Myopia case-control status was defined based on UKB data field 6147 (Reason for glasses/contact lenses: For short-sightedness). However, the researchers did not take into account that only 25% of UK Biobank participants were asked this question. Participants who were not asked the question were categorized as controls, which resulted in severe misclassification bias (myopia prevalence inferred to be 8% rather than the true prevalence of ~38%).

**ukb-a-419**. This GWAS for myopia was conducted in 2017 in a sample of 335,700 individuals from the UK Biobank by the Neale Lab. A total of 10,894,596 SNPs were tested using the linear regression model using Hail. A comprehensive description of the pipeline is provided in their Github repository (https://github.com/Nealelab/UK_Biobank_GWAS/tree/master/imputed-v2-gwas#association-in-hail). Myopia case-control status was defined based on UKB data field 6147 (Reason for glasses/contact lenses: For short-sightedness). However, the researchers did not take into account that only 25% of UK Biobank participants were asked this question. Participants who were not asked the question were categorized as controls, which resulted in severe misclassification bias (myopia prevalence inferred to be 8% rather than the true prevalence of ~38%).

**H7_MYOPIA and finn-b-H7_MYOPIA.**^4^ The FinnGen consortium have published eleven sets of GWAS summary statistic for myopia (one set for each of the eleven FinnGen data releases). The most recent public data release (R12) was in November 24, 2024; this included 482,182 individuals in the myopia association analysis (5,406 cases; 476,776 controls). A total of 21,311,644 variants were tested using SAIGE (v.0.35.8.8). Earlier releases used in myopia MR studies included 1,640 cases and 210,931 controls (R5), 3,534 cases and 361,237 controls (R9) and 4,106 cases and 394,028 controls (R10). Myopia was defined based on the ICD code H52.1 from EHR. Since myopia is rarely listed in EHR, this resulted in severe misclassification bias (myopia prevalence inferred to be approximately 1%).

# Supplementary Note S2. Transformation of the effect sizes and standard errors of IEU OpenGWAS and the Neale lab’s myopia GWAS summary statistics

The effect sizes and standard errors of the two linear model myopia GWAS were transformed and adjusted using the following formulas:

$$logOR= \frac{\beta_{linear}}{\mu(1-\mu)}$$

$$se\left( logOR \right)= \frac{\mathrm{se}_{linear}}{\mu\left( 1-\mu\right)}$$

where β_linear_ is the effect size (beta coefficient) for a given SNP on the absolute risk difference scale, se_linear_ is the standard error of the linear effect size, and $\mu$is the case prevalence in the investigated cohort: $\mu=n_{case}/(n_{case}+ n_{control})$.

# Supplementary Note S3. Newly-performed GWAS for myopia

*Sample selection*. We identified UK Biobank participants who underwent refractive error measurement (Tomey RC5000 autorefractor; data field #5084-5088). The SER for each eye was calculated as: spherical power + (cylindrical power * 0.5). Participants were classified as myopia cases if the SER ≤ -0.50 diopters (D) in at least one eye. Controls were classified as individuals with SER > -0.50 D in both eyes. To ensure that the GWAS sample was drawn from the same ancestry group as individuals in SSGAC and the Gharahkhani et al.^5^ GWAS, we restricted the analysis to participants of European ancestry (defined as individuals whose first two genetic principal components [PCs; data field #22009] were within the mean ± 3 standard deviations of all unrelated UK Biobank participants who self-reported as White British). Additionally, individuals were required to have heterozygosity [data field #22004] within the mean ± 4 standard deviations of all unrelated participants who self-reported as White British. Subsequently, participants were excluded if their missing genotyping rate [data field #22005] exceeded 0.05 or if their genetic sex [data field #22001] did not match their self-reported sex. Lastly, we retained participants who had no history of eye surgery (cataract [data field #5324], corneal [data field #5328], strabismus [data field #6147]). This resulted in a sample of 93,041 related individuals and a sample of 66,776 unrelated individuals. The age of the participants at the assessment visit was calculated from their date of visit [data field #53] and their month and year of birth [data fields #52 and #34, respectively].

*Association analyses.* A GWAS for myopia in unrelated individuals was carried out with logistic-Firth hybrid regression model using Plink2.^6^ Age, sex, genotyping array, and the first ten PCs were included as covariates. A total of 9,802,868 imputed genetic variants with minor allele frequency (MAF) > 0.01 and per variant genotyping call rate ≥ 0.9 were tested in 66,773 individuals (25,804 cases : 40,969 controls). A GWAS that included related individuals was performed using SAIGE/GATE v0.44. Age, sex, genotyping array, and the first four PCs were included as covariates, following the covariates used by Zhou et al.^1^ A total of 9,850,118 imputed genetic variants with minor allele frequency MAF > 0.01 and imputation quality INFO > 0.6 were tested on 93,036 individuals (35,531 cases : 57,505 controls).

# Supplementary Note S4. Selection of instrumental variables

***Calculation of R^2^ and F-statistic***

For a continuous trait, the proportion of variance explained by an instrument (R^2^) was calculated using the formula: R^2^ = β^2^ / (β^2^ + (se(β)^2^ * N)), where β is the effect size (beta coefficient) for a given SNP, se(β) is the standard error of the effect size, and N is the GWAS sample size.

For a binary trait, R^2^ liability was calculated based on the R^2^ logit liability scale^7^: R^2^_l_ = Vg/(Vg+Ve), where Vg = β^2^*popEAF*(1-popEAF), Ve = π^2^/3, and popEAF is the allele frequency of the effect allele in the population.

The F-statistic was calculated using the following formula: F = R^2^ * (N - 1 - k) / ((1 - R^2^) * k), where R^2^ represents the proportion of variance (or variance of liability) explained by the instrument, N denotes the sample size, and k denotes the number of SNPs included in the instrument (k = 1 for calculating the F-statistic for each SNP).

***Selection of instrumental variables for years of education***

LD between variants in the summary statistics of Okbay et al.^8^ was checked with the GBR population as the LD reference panel. GWAS variants independently associated with EduYears at P < 5 x 10^-8^ were selected. Subsequently, variants not included in the Haplotype Reference Consortium (HRC) were excluded, as were SNPs not present in any of the myopia GWAS datasets, to ensure consistency in the genetic instrumental variables utilized across all of the MR analyses. F-statistics and variance explained (R^2^) were used to assess the strength of genetic instruments. Variants with an F-statistic below 10 were excluded.^9^

***Selection of instrumental variables for myopia***

We used a consistent approach to select IVs for myopia from each of the nine sets of GWAS summary statistics for myopia listed in Table 1. We first excluded variants not available in the summary statistics for POAG^5^ or not available in the clumping reference panel (N = 10,000 UK Biobank participants of European ancestry). Variants were clumped using PLINK v1.9 using a p-value threshold of *P* < 5.0e-08, a distance metric of ±1000 kb and a linkage disequilibrium (LD) threshold of *r*^2^ < 0.05.^6^ Genetic variants with an F-statistic below 10 were excluded.^9^

# Supplementary Note S5. Mendelian randomization analysis of the two exemplar testcases

IVW-MR, MR-EGGER, weighted median MR, and mode-based MR analyses were carried out using the R package MendelianRandomization (version 0.10.0).^10^ MR-PRESSO analysis was carried out using the R package MR-PRESSO (version 1.0).^11^ The R package LDlinkR^12^ (version 1.4.0) was used to check for LD between variants in the summary statistics of Okbay et al.,^8^ with the GBR population as the LD reference panel. R software was utilized in version 4.4.3. Details of the 62 IVs used in the education-myopia MR can be found in Supplementary Table S1. These 62 IVs were selected from the 74 genome-wide significant variants reported by Okbay et al.^8^ after excluding variants in LD (*r*^2^ > 0.05) and excluding variants not available in all ten sets of myopia summary statistics.

# Supplementary Note S6. Search strategy, inclusion criteria, and list of papers excluded for MR re-analysis

To find previously published Mendelian randomization studies of myopia, we employed the following search strategy via PubMed (queried on 4^th^ of July, 2025):

| #1 | ((myopia[MeSH Terms]) OR (myopic[Title/Abstract]) OR (myopia[Title/Abstract]) OR (myope[Title/Abstract]) OR (nearsighted* [Title/Abstract]) OR (near-sightedness[Title/Abstract]) OR (shortsighted*[Title/Abstract]) OR (short-sightedness[Title/Abstract]) OR (“refractive error*”[Title/Abstract])) |
| --- | --- |
| #2 | (“Mendelian randomization”[Title/Abstract]) |
|  | #1 AND #2 |

We identified a total of 59 relevant articles published between 2016 and 2025, with the majority appearing in 2024 and 2025. These previous Mendelian randomization studies on myopia were conducted using summary statistics derived from three primary variable types: (1) continuous refractive error (21 studies), (2) dichotomous myopia case-control status (35 studies), and (3) other myopia-related traits, including high myopia, polygenic risk score for age of onset myopia, axial length (AL), corneal radius (CR), and the AL/CR ratio (3 studies).

Since our analyses focused on GWAS analyses of myopia as a binary trait, we included only studies that met all of the following criteria for re-analysis:

(1) Utilized publicly existing GWAS summary statistics for myopia case-control status;

(2) Employed two non-overlapping samples for the exposure and outcome GWAS in the original MR study;

(3) Did not include any GWAS summary statistics that overlapped with our newly derived myopia GWAS datasets;

(4) Reported a significant causal relationship between two variables, with a non-trivial effect size;

(5) Did not apply overly liberal clumping threshold when selecting genetic instruments (e.g., avoided criteria such as p < 5e-5, *r*^2^ < 0.1, and within ±500kb);

(6) Used ancestrally-matched samples for the exposure and outcome GWAS;

(7) Provided a publicly downloadable summary statistics source;

(8) Contained no conflicting information for genetic variants (i.e., no duplicate variant IDs with inconsistent beta and standard error statistics).

Of the 35 Mendelian randomization studies that used GWAS summary statistics of myopia case-control status, we excluded a total of 27 studies based on the following criteria: 3 studies that generated their own myopia GWAS summary statistics^13-15^; one study in which the summary statistics of the other variable were unavailable^16^; one study whose summary statistics for the other variable contained problematic duplicate variants^17^; 5 studies that used heavily overlapping samples for the exposure and outcome GWAS ^18-23^; 6 studies in which the GWAS sample overlapped with our newly derived myopia GWAS ^23-28^; 4 studies that reported only minimal causal estimates^29-32^; 2 studies that applied overly liberal clumping thresholds^33, 34^; 2 studies that used mixed-ethnicity sample^35, 36^; and 3 studies that did not report any significant association ^21, 37, 38^. As a result, 8 studies were retained for re-analysis.

# Supplementary Note S7. Statistical code to reproduce the analyses.

library(LDlinkR)

library(data.table)

library(MendelianRandomization)

library(MRPRESSO)

library(plyr)

library(ggplot2)

library(cowplot)

rm(list=ls())

####

mydir <- "[ path to folder containing data ]"

mytoken <- "[ X ]"

####

# Files

# -----

okbay_sumstats_file <- paste0(mydir, "Okbay_SNPs_2025-03-17.csv")

okbay_ldmat_file <- paste0(mydir, "Okbay_SNPs_LDmatrix_2025-03-17.csv")

merged_sumstats_file <- paste0(mydir, "Okbay_Merged_2025-03-17.csv")

tableS2_file <- paste0(mydir, "Manuscript/TableS2_2025-05-25.csv")

table2_file <- paste0(mydir, "Manuscript/Table2_2025-05-25.csv")

tableS1_file <- paste0(mydir, "Manuscript/TableS1_2025-05-25.csv")

final_snplist_file <- paste0(mydir, "Okbay_MR-SNPlist_2025-03-17.csv")

# Parameters

# ----------

snp_ld_r2_threshold <- 0.05

convert_eduyears_sd <- 3.6

skip_mr_presso <- FALSE

# Read in Okbay SNP sumstats and calculate SE (n=74 SNPs)

# -------------------------------------------------------

dataOKBAY <- read.csv(file=okbay_sumstats_file, header=TRUE)

names(dataOKBAY) <- c("SNP","CHR","POS_GRCh37","EA","FreqEA","BETA_TEMP","P_OKBAY")

dataOKBAY$BETA_OKBAY <- dataOKBAY$BETA_TEMP*convert_eduyears_sd

dataOKBAY$Z <- sign(dataOKBAY$BETA_OKBAY) * abs( qnorm(dataOKBAY$P_OKBAY/2))

dataOKBAY$SE_OKBAY <- dataOKBAY$BETA_OKBAY/dataOKBAY$Z

dataOKBAY$Z <- NULL

num_snps <- nrow(dataOKBAY)

# Create a squared cor^2 (R2) LD matrix using LDlink

# --------------------------------------------------

if(file.exists(okbay_ldmat_file)!=TRUE){

progress_max <- num_snps^2

ldmatOKBAY <- matrix(nrow=num_snps, ncol=num_snps)

rownames(ldmatOKBAY) <- dataOKBAY$SNP

colnames(ldmatOKBAY) <- dataOKBAY$SNP

for(s1 in 1:num_snps){

for(s2 in 1:num_snps){

if(s1==s2){

ldmatOKBAY[s1,s2] <- 1

} else {

if(dataOKBAY$CHR[s1]==dataOKBAY$CHR[s2]){

x <- suppressMessages(LDmatrix(snps = c(dataOKBAY$SNP[s1],dataOKBAY$SNP[s2]), pop = "GBR", r2d = "r2", token = mytoken))

ldmatOKBAY[s1,s2] <- ifelse(ncol(x)==3, x[1,3], NA)

ldmatOKBAY[s2,s1] <- ifelse(ncol(x)==3, x[1,3], NA)

} else {

ldmatOKBAY[s1,s2] <- 0

ldmatOKBAY[s2,s1] <- 0

}

}

}

}

write.csv(ldmatOKBAY, file=okbay_ldmat_file, row.names=FALSE)

}

###############################

# HARMONIZE SUMSTATS

# ------------------

# FinnGen R5

mydir2="/scratch/c.sopjg2/MR_2025/"

okbay_file="Nga_74_Okbay_SNPs_2025-03-17.csv"

sumstats_file="finngen_R5_H7_MYOPIA.gz"

snp_file="okbay_snps74.txt"

merge_file="finngen_R5_H7_MYOPIA_okbay_snps74.txt"

# [system commands]

# cd ${mydir2}

# tail -n +2 ${okbay_file} | awk 'BEGIN {FS=","}{print $1}' > ${snp_file}

# gunzip -c ${sumstats_file} | head -1 > ${merge_file}

# gunzip -c ${sumstats_file} | grep -w -f ${snp_file} >> ${merge_file}

# sed -e 's/#//' ${merge_file} > temp.txt

# mv temp.txt ${merge_file}

# cp ${merge_file} ${mydir}${merge_file}

dataFinnGenR5 <- as.data.frame(fread(file=paste0(mydir,merge_file), header=TRUE))

dataFinnGenR5_1 <- dataFinnGenR5[,c("rsids","chrom","pos","ref","alt","beta","sebeta","pval")]

names(dataFinnGenR5_1) <- c("SNP","CHR","POS_GRCh38","NEA_FinnGenR5","EA_FinnGenR5","BETA_Temp","SE_FinnGenR5","P_FinnGenR5")

dataM <- merge(dataOKBAY,dataFinnGenR5_1, by=c("SNP","CHR"), all=TRUE)

dataM$BETA_FinnGenR5 <- ifelse(dataM$EA==dataM$EA_FinnGenR5, dataM$BETA_Temp, NA)

dataM$BETA_FinnGenR5 <- ifelse(dataM$EA==dataM$NEA_FinnGenR5, -1*dataM$BETA_Temp, dataM$BETA_FinnGenR5)

dataM$NEA_FinnGenR5 <- NULL

dataM$EA_FinnGenR5 <- NULL

dataM$BETA_Temp <- NULL

dataM$POS_GRCh38 <- NULL

write.csv(dataM, file=merged_sumstats_file, row.names=FALSE)

# FinnGen R9

mydir2="/scratch/c.sopjg2/MR_2025/"

okbay_file="Nga_74_Okbay_SNPs_2025-03-17.csv"

sumstats_file="finngen_R9_H7_MYOPIA.gz"

snp_file="okbay_snps74.txt"

merge_file="finngen_R9_H7_MYOPIA_okbay_snps74.txt"

# [system commands]

# cd ${mydir2}

# tail -n +2 ${okbay_file} | awk 'BEGIN {FS=","}{print $1}' > ${snp_file}

# gunzip -c ${sumstats_file} | head -1 > ${merge_file}

# gunzip -c ${sumstats_file} | grep -w -f ${snp_file} >> ${merge_file}

# sed -e 's/#//' ${merge_file} > temp.txt

# mv temp.txt ${merge_file}

# cp ${merge_file} ${mydir}${merge_file}

dataOKBAY <- as.data.frame(fread(file=merged_sumstats_file, header=TRUE))

dataFinnGenR9 <- as.data.frame(fread(file=paste0(mydir,merge_file), header=TRUE))

dataFinnGenR9_1 <- dataFinnGenR9[,c("rsids","chrom","pos","ref","alt","beta","sebeta","pval")]

names(dataFinnGenR9_1) <- c("SNP","CHR","POS_GRCh38","NEA_FinnGenR9","EA_FinnGenR9","BETA_Temp","SE_FinnGenR9","P_FinnGenR9")

dataM <- merge(dataOKBAY,dataFinnGenR9_1, by=c("SNP","CHR"), all=TRUE)

dataM$BETA_FinnGenR9 <- ifelse(dataM$EA==dataM$EA_FinnGenR9, dataM$BETA_Temp, NA)

dataM$BETA_FinnGenR9 <- ifelse(dataM$EA==dataM$NEA_FinnGenR9, -1*dataM$BETA_Temp, dataM$BETA_FinnGenR9)

dataM$NEA_FinnGenR9 <- NULL

dataM$EA_FinnGenR9 <- NULL

dataM$BETA_Temp <- NULL

dataM$POS_GRCh38 <- NULL

write.csv(dataM, file=merged_sumstats_file, row.names=FALSE)

# FinnGen R10

mydir2="/scratch/c.sopjg2/MR_2025/"

okbay_file="Nga_74_Okbay_SNPs_2025-03-17.csv"

sumstats_file="finngen_R10_H7_MYOPIA.gz"

snp_file="okbay_snps74.txt"

merge_file="finngen_R10_H7_MYOPIA_okbay_snps74.txt"

# [system commands]

# cd ${mydir2}

# tail -n +2 ${okbay_file} | awk 'BEGIN {FS=","}{print $1}' > ${snp_file}

# gunzip -c ${sumstats_file} | head -1 > ${merge_file}

# gunzip -c ${sumstats_file} | grep -w -f ${snp_file} >> ${merge_file}

# sed -e 's/#//' ${merge_file} > temp.txt

# mv temp.txt ${merge_file}

# cp ${merge_file} ${mydir}${merge_file}

dataOKBAY <- as.data.frame(fread(file=merged_sumstats_file, header=TRUE))

dataFinnGenR10 <- as.data.frame(fread(file=paste0(mydir,merge_file), header=TRUE))

dataFinnGenR10_1 <- dataFinnGenR10[,c("rsids","chrom","pos","ref","alt","beta","sebeta","pval")]

names(dataFinnGenR10_1) <- c("SNP","CHR","POS_GRCh38","NEA_FinnGenR10","EA_FinnGenR10","BETA_Temp","SE_FinnGenR10","P_FinnGenR10")

dataM <- merge(dataOKBAY,dataFinnGenR10_1, by=c("SNP","CHR"), all=TRUE)

dataM$BETA_FinnGenR10 <- ifelse(dataM$EA==dataM$EA_FinnGenR10, dataM$BETA_Temp, NA)

dataM$BETA_FinnGenR10 <- ifelse(dataM$EA==dataM$NEA_FinnGenR10, -1*dataM$BETA_Temp, dataM$BETA_FinnGenR10)

dataM$NEA_FinnGenR10 <- NULL

dataM$EA_FinnGenR10 <- NULL

dataM$BETA_Temp <- NULL

dataM$POS_GRCh38 <- NULL

write.csv(dataM, file=merged_sumstats_file, row.names=FALSE)

# FinnGen R11

mydir2="/scratch/c.sopjg2/MR_2025/"

okbay_file="Nga_74_Okbay_SNPs_2025-03-17.csv"

sumstats_file="finngen_R11_H7_MYOPIA.gz"

snp_file="okbay_snps74.txt"

merge_file="finngen_R11_H7_MYOPIA_okbay_snps74.txt"

# [system commands]

# cd ${mydir2}

# tail -n +2 ${okbay_file} | awk 'BEGIN {FS=","}{print $1}' > ${snp_file}

# gunzip -c ${sumstats_file} | head -1 > ${merge_file}

# gunzip -c ${sumstats_file} | grep -w -f ${snp_file} >> ${merge_file}

# sed -e 's/#//' ${merge_file} > temp.txt

# mv temp.txt ${merge_file}

# cp ${merge_file} ${mydir}${merge_file}

dataOKBAY <- as.data.frame(fread(file=merged_sumstats_file, header=TRUE))

dataFinnGenR11 <- as.data.frame(fread(file=paste0(mydir,merge_file), header=TRUE))

dataFinnGenR11_1 <- dataFinnGenR11[,c("rsids","chrom","pos","ref","alt","beta","sebeta","pval")]

names(dataFinnGenR11_1) <- c("SNP","CHR","POS_GRCh38","NEA_FinnGenR11","EA_FinnGenR11","BETA_Temp","SE_FinnGenR11","P_FinnGenR11")

dataM <- merge(dataOKBAY,dataFinnGenR11_1, by=c("SNP","CHR"), all=TRUE)

dataM$BETA_FinnGenR11 <- ifelse(dataM$EA==dataM$EA_FinnGenR11, dataM$BETA_Temp, NA)

dataM$BETA_FinnGenR11 <- ifelse(dataM$EA==dataM$NEA_FinnGenR11, -1*dataM$BETA_Temp, dataM$BETA_FinnGenR11)

dataM$NEA_FinnGenR11 <- NULL

dataM$EA_FinnGenR11 <- NULL

dataM$BETA_Temp <- NULL

dataM$POS_GRCh38 <- NULL

write.csv(dataM, file=merged_sumstats_file, row.names=FALSE)

# New PLINK GWAS in UKBB (unrelated)

mydir2="/scratch/c.sopjg2/MR_2025/"

okbay_file="Nga_74_Okbay_SNPs_2025-03-17.csv"

sumstats_file="/scratch/scw1193/Nga/MR_EduYears_Myopia/results/myopia_noed_plink2_chrALL_MR.txt"

snp_file="okbay_snps74.txt"

merge_file="plink_unrelated_okbay_snps74.txt"

# [system commands]

# cd ${mydir2}

# tail -n +2 ${okbay_file} | awk 'BEGIN {FS=","}{print $1}' > ${snp_file}

# head -1 ${sumstats_file} > ${merge_file}

# grep -w -f ${snp_file} ${sumstats_file} >> ${merge_file}

# sed -e 's/#//' ${merge_file} > temp.txt

# mv temp.txt ${merge_file}

# cp ${merge_file} ${mydir}${merge_file}

dataOKBAY <- as.data.frame(fread(file=merged_sumstats_file, header=TRUE))

dataPLINK <- as.data.frame(fread(file=paste0(mydir,merge_file), header=TRUE))

dataPLINK <- read.table(file=paste0(mydir,merge_file), header=TRUE)

dataPLINK$EA <- dataPLINK$A1

dataPLINK$NEA <- ifelse(dataPLINK$EA==dataPLINK$REF, dataPLINK$ALT, dataPLINK$REF)

dataPLINK_1 <- dataPLINK[,c("ID","CHROM","POS","NEA","EA","BETA","SE","p_value")]

names(dataPLINK_1 ) <- c("SNP","CHR","POS_GRCh38","NEA_PLINK","EA_PLINK","BETA_Temp","SE_PLINK","P_PLINK")

dataM <- merge(dataOKBAY,dataPLINK_1, by=c("SNP","CHR"), all=TRUE)

dataM$BETA_PLINK <- ifelse(dataM$EA==dataM$EA_PLINK, dataM$BETA_Temp, NA)

dataM$BETA_PLINK <- ifelse(dataM$EA==dataM$NEA_PLINK, -1*dataM$BETA_Temp, dataM$BETA_PLINK)

dataM$NEA_PLINK <- NULL

dataM$EA_PLINK <- NULL

dataM$BETA_Temp <- NULL

dataM$POS_GRCh38 <- NULL

write.csv(dataM, file=merged_sumstats_file, row.names=FALSE)

# New SAIGE GWAS in UKBB (including relatives)

mydir2="/scratch/c.sopjg2/MR_2025/"

okbay_file="Nga_74_Okbay_SNPs_2025-03-17.csv"

sumstats_file="/scratch/scw1193/Nga/MR_EduYears_Myopia/results/sumstat_myopia_no_ed_gate_MR.txt"

snp_file="okbay_snps74.txt"

merge_file="saige_related_okbay_snps74.txt"

# [system commands]

# cd ${mydir2}

# tail -n +2 ${okbay_file} | awk 'BEGIN {FS=","}{print $1}' > ${snp_file}

# head -1 ${sumstats_file} > ${merge_file}

# grep -w -f ${snp_file} ${sumstats_file} >> ${merge_file}

# sed -e 's/#//' ${merge_file} > temp.txt

# mv temp.txt ${merge_file}

# cp ${merge_file} ${mydir}${merge_file}

dataOKBAY <- as.data.frame(fread(file=merged_sumstats_file, header=TRUE))

dataSAIGE <- as.data.frame(fread(file=paste0(mydir,merge_file), header=TRUE))

dataSAIGE <- read.table(file=paste0(mydir,merge_file), header=TRUE)

dataSAIGE_1 <- dataSAIGE[,c("rsid","CHR","POS","REF","ALT","BETA","SE","p.value")]

names(dataSAIGE_1 ) <- c("SNP","CHR","POS_GRCh38","NEA_SAIGE","EA_SAIGE","BETA_Temp","SE_SAIGE","P_SAIGE")

dataM <- merge(dataOKBAY,dataSAIGE_1, by=c("SNP","CHR"), all=TRUE)

dataM$BETA_SAIGE <- ifelse(dataM$EA==dataM$EA_SAIGE, dataM$BETA_Temp, NA)

dataM$BETA_SAIGE <- ifelse(dataM$EA==dataM$NEA_SAIGE, -1*dataM$BETA_Temp, dataM$BETA_SAIGE)

dataM$NEA_SAIGE <- NULL

dataM$EA_SAIGE <- NULL

dataM$BETA_Temp <- NULL

dataM$POS_GRCh38 <- NULL

write.csv(dataM, file=merged_sumstats_file, row.names=FALSE)

# GCST90044326 (Jiang et al.)

mydir2="/scratch/c.sopjg2/MR_2025/"

okbay_file="Nga_74_Okbay_SNPs_2025-03-17.csv"

sumstats_file="GCST90044326_buildGRCh37.tsv.gz"

snp_file="okbay_snps74.txt"

merge_file="jiang_MYOPIA_okbay_snps74.txt"

# [system commands]

# cd ${mydir2}

# tail -n +2 ${okbay_file} | awk 'BEGIN {FS=","}{print $1}' > ${snp_file}

# gunzip -c ${sumstats_file} | head -1 > ${merge_file}

# gunzip -c ${sumstats_file} | grep -w -f ${snp_file} >> ${merge_file}

# sed -e 's/#//' ${merge_file} > temp.txt

# mv temp.txt ${merge_file}

# cp ${merge_file} ${mydir}${merge_file}

dataOKBAY <- as.data.frame(fread(file=merged_sumstats_file, header=TRUE))

dataJIANG <- as.data.frame(fread(file=paste0(mydir,merge_file), header=TRUE))

dataJIANG_1 <- dataJIANG[,c("variant_id","chromosome","base_pair_location","other_allele","effect_allele","beta","standard_error","p_value")]

names(dataJIANG_1) <- c("SNP","CHR","POS_GRCh38","NEA_JIANG","EA_JIANG","BETA_Temp","SE_JIANG","P_JIANG")

dataM <- merge(dataOKBAY,dataJIANG_1, by=c("SNP","CHR"), all=TRUE)

dataM$BETA_JIANG <- ifelse(dataM$EA==dataM$EA_JIANG, dataM$BETA_Temp, NA)

dataM$BETA_JIANG <- ifelse(dataM$EA==dataM$NEA_JIANG, -1*dataM$BETA_Temp, dataM$BETA_JIANG)

dataM$NEA_JIANG <- NULL

dataM$EA_JIANG <- NULL

dataM$BETA_Temp <- NULL

dataM$POS_GRCh38 <- NULL

write.csv(dataM, file=merged_sumstats_file, row.names=FALSE)

# GCST90435990 (Zhou et al.)

mydir2="/scratch/c.sopjg2/MR_2025/"

okbay_file="Nga_74_Okbay_SNPs_2025-03-17.csv"

sumstats_file="GCST90435990.tsv.gz"

snp_file="okbay_snps74.txt"

merge_file="zhou_MYOPIA_okbay_snps74.txt"

# [system commands]

# cd ${mydir2}

# tail -n +2 ${okbay_file} | awk 'BEGIN {FS=","}{print $1}' > ${snp_file}

# gunzip -c ${sumstats_file} | head -1 > ${merge_file}

# gunzip -c ${sumstats_file} | grep -w -f ${snp_file} >> ${merge_file}

# sed -e 's/#//' ${merge_file} > temp.txt

# mv temp.txt ${merge_file}

# cp ${merge_file} ${mydir}${merge_file}

dataOKBAY <- as.data.frame(fread(file=merged_sumstats_file, header=TRUE))

dataZHOU <- as.data.frame(fread(file=paste0(mydir,merge_file), header=TRUE))

dataZHOU_1 <- dataZHOU[,c("variant_id","chromosome","base_pair_location","other_allele","effect_allele","beta","standard_error","p_value")]

names(dataZHOU_1) <- c("SNP","CHR","POS_GRCh38","NEA_ZHOU","EA_ZHOU","BETA_Temp","SE_ZHOU","P_ZHOU")

dataM <- merge(dataOKBAY,dataZHOU_1, by=c("SNP","CHR"), all=TRUE)

dataM$BETA_ZHOU <- ifelse(dataM$EA==dataM$EA_ZHOU, dataM$BETA_Temp, NA)

dataM$BETA_ZHOU <- ifelse(dataM$EA==dataM$NEA_ZHOU, -1*dataM$BETA_Temp, dataM$BETA_ZHOU)

dataM$NEA_ZHOU <- NULL

dataM$EA_ZHOU <- NULL

dataM$BETA_Temp <- NULL

dataM$POS_GRCh38 <- NULL

write.csv(dataM, file=merged_sumstats_file, row.names=FALSE)

# ukb-a-419 (Neale Lab)

mydir2="/scratch/c.sopjg2/MR_2025/"

okbay_file="Nga_74_Okbay_SNPs_2025-03-17.csv"

sumstats_file="ukb-a-419.vcf.gz"

snp_file="okbay_snps74.txt"

merge_file="neale_MYOPIA_okbay_snps74.txt"

# [system commands]

# cd ${mydir2}

# tail -n +2 ${okbay_file} | awk 'BEGIN {FS=","}{print $1}' > ${snp_file}

# echo "SNP CHR POS REF ALT BETA SE LOG10_P" > ${merge_file}

# gunzip -c ${sumstats_file} | grep -w -f ${snp_file} > temp.txt

# awk '{split($10,a,":")}{print $3,$1,$2,$4,$5,a[1],a[2],a[3]}' temp.txt >> ${merge_file}

# sed -e 's/#//' ${merge_file} > temp.txt

# mv temp.txt ${merge_file}

# cp ${merge_file} ${mydir}${merge_file}

dataOKBAY <- as.data.frame(fread(file=merged_sumstats_file, header=TRUE))

dataNEALE <- as.data.frame(fread(file=paste0(mydir,merge_file), header=TRUE))

dataNEALE$P <- 10^(-1*dataNEALE$LOG10_P)

dataNEALE_1 <- dataNEALE[,c("SNP","CHR","POS","REF","ALT","BETA","SE","P")]

names(dataNEALE_1) <- c("SNP","CHR","POS_GRCh38","NEA_NEALE","EA_NEALE","BETA_Temp","SE_NEALE","P_NEALE")

dataM <- merge(dataOKBAY,dataNEALE_1, by=c("SNP","CHR"), all=TRUE)

dataM$BETA_NEALE <- ifelse(dataM$EA==dataM$EA_NEALE, dataM$BETA_Temp, NA)

dataM$BETA_NEALE <- ifelse(dataM$EA==dataM$NEA_NEALE, -1*dataM$BETA_Temp, dataM$BETA_NEALE)

dataM$NEA_NEALE <- NULL

dataM$EA_NEALE <- NULL

dataM$BETA_Temp <- NULL

dataM$POS_GRCh38 <- NULL

write.csv(dataM, file=merged_sumstats_file, row.names=FALSE)

# ukb-b-6353 (MRCIEU)

mydir2="/scratch/c.sopjg2/MR_2025/"

okbay_file="Nga_74_Okbay_SNPs_2025-03-17.csv"

sumstats_file="ukb-b-6353.vcf.gz"

snp_file="okbay_snps74.txt"

merge_file="mrcieu_MYOPIA_okbay_snps74.txt"

# [system commands]

# cd ${mydir2}

# tail -n +2 ${okbay_file} | awk 'BEGIN {FS=","}{print $1}' > ${snp_file}

# echo "SNP CHR POS REF ALT BETA SE LOG10_P" > ${merge_file}

# gunzip -c ${sumstats_file} | grep -w -f ${snp_file} > temp.txt

# awk '{split($10,a,":")}{print $3,$1,$2,$4,$5,a[1],a[2],a[3]}' temp.txt >> ${merge_file}

# sed -e 's/#//' ${merge_file} > temp.txt

# mv temp.txt ${merge_file}

# cp ${merge_file} ${mydir}${merge_file}

dataOKBAY <- as.data.frame(fread(file=merged_sumstats_file, header=TRUE))

dataMRCIEU <- as.data.frame(fread(file=paste0(mydir,merge_file), header=TRUE))

dataMRCIEU$P <- 10^(-1*dataMRCIEU$LOG10_P)

dataMRCIEU_1 <- dataMRCIEU[,c("SNP","CHR","POS","REF","ALT","BETA","SE","P")]

names(dataMRCIEU_1) <- c("SNP","CHR","POS_GRCh38","NEA_MRCIEU","EA_MRCIEU","BETA_Temp","SE_MRCIEU","P_MRCIEU")

dataM <- merge(dataOKBAY,dataMRCIEU_1, by=c("SNP","CHR"), all=TRUE)

dataM$BETA_MRCIEU <- ifelse(dataM$EA==dataM$EA_MRCIEU, dataM$BETA_Temp, NA)

dataM$BETA_MRCIEU <- ifelse(dataM$EA==dataM$NEA_MRCIEU, -1*dataM$BETA_Temp, dataM$BETA_MRCIEU)

dataM$NEA_MRCIEU <- NULL

dataM$EA_MRCIEU <- NULL

dataM$BETA_Temp <- NULL

dataM$POS_GRCh38 <- NULL

write.csv(dataM, file=merged_sumstats_file, row.names=FALSE)

###############################

# MENDELIAN RANDOMIZATION

# -----------------------

ldmatOKBAY <- as.matrix(read.csv(file=okbay_ldmat_file, header=TRUE))

rownames(ldmatOKBAY) <- colnames(ldmatOKBAY)

num_snps <- nrow(ldmatOKBAY)

dataT <- as.data.frame(cbind(colnames(ldmatOKBAY),1:num_snps))

names(dataT) <- c("SNP","SortOrder")

dataOKBAY <- read.csv(file=merged_sumstats_file, header=TRUE)

dataOKBAY <- merge(dataOKBAY,dataT,by="SNP")

dataOKBAY <- dataOKBAY[order(as.numeric(dataOKBAY$SortOrder)),]

# Remove SNPs not present in all datasets

dataFULL <- dataOKBAY[complete.cases(dataOKBAY),]

bad_snps <- as.numeric(dataOKBAY[!dataOKBAY$SNP %in% dataFULL$SNP,]$SortOrder)

ldmatOKBAY <- ldmatOKBAY[-bad_snps,-bad_snps]

num_snps <- num_snps - length(bad_snps)

# Remove SNPs in LD

removed_snps <- NULL

ldOK <- 0

while(ldOK==0){

bad_ld_snps <- NULL

for (row_snp in 1:num_snps){

for (col_snp in row_snp:num_snps){

x <- ifelse(is.na(ldmatOKBAY[col_snp, row_snp]),0,ldmatOKBAY[col_snp, row_snp])

if(x > snp_ld_r2_threshold & col_snp!=row_snp){ bad_ld_snps <- c(bad_ld_snps, col_snp, row_snp) }

}

}

if(length(bad_ld_snps)>0){

x1 <- as.data.frame(table(bad_ld_snps))

x2 <- x1[order(-x1$Freq),]

worst_snp <- as.numeric(as.character(x2$bad_ld_snps[1]))

removed_snps <- c(removed_snps, colnames(ldmatOKBAY)[worst_snp])

ldmatOKBAY <- ldmatOKBAY[-worst_snp,-worst_snp]

num_snps <- num_snps - 1

}

if(length(bad_ld_snps)==0){ ldOK=1 }

}

# Create a table for the results

fullres <- as.data.frame(matrix(ncol=14, nrow=2))

names(fullres) <- c("Method","Sumstats","Num_IVs","OR","LCI95","UCI95","BETA","SE","P","Qstat", "Qpval","Egger_intercept","Egger_P","MRP_outliers")

myrow <- 1

# Run MR

sumstats_list <- c("JIANG","ZHOU","NEALE","MRCIEU","FinnGenR5","FinnGenR9","FinnGenR10","FinnGenR11","SAIGE","PLINK")

dataMR <- dataFULL[!dataFULL$SNP %in% removed_snps,]

for(k in 1:length(sumstats_list)){

mystats <- sumstats_list[k]

mybeta <- paste0("BETA_",mystats)

myse <- paste0("SE_",mystats)

dataMR$BETA_OUT <- dataMR[,mybeta]

dataMR$SE_OUT <- dataMR[,myse]

MR_obj <- MendelianRandomization::mr_input(exposure = "EduYears",

outcome = "Myopia status",

snps = dataMR$SNP,

bx = dataMR$BETA_OKBAY,

bxse = dataMR$SE_OKBAY,

by = dataMR[,mybeta],

byse = dataMR[,myse])

MR_ivw <- MendelianRandomization::mr_ivw (MR_obj, robust = FALSE, distribution = "normal", penalized = FALSE, alpha = 0.05, correl = FALSE)

fullres[myrow,1] <- "IVW-MR"

fullres[myrow,2] <- mystats

fullres[myrow,3] <- MR_ivw$SNPs

fullres[myrow,4] <- sprintf("%.3f", exp(MR_ivw$Estimate))

fullres[myrow,5] <- sprintf("%.3f", exp(MR_ivw$CILower))

fullres[myrow,6] <- sprintf("%.3f", exp(MR_ivw$CIUpper))

fullres[myrow,7] <- sprintf("%.3f", MR_ivw$Estimate)

fullres[myrow,8] <- sprintf("%.3f", MR_ivw$StdError)

fullres[myrow,9] <- sprintf("%.2e", MR_ivw$Pvalue)

fullres[myrow,10] <- sprintf("%.3f", MR_ivw$Heter.Stat[1])

fullres[myrow,11] <- sprintf("%.3f", MR_ivw$Heter.Stat[2])

fullres[myrow,12:14] <- c("-","-","-")

myrow <- myrow + 1

MR_egg <- MendelianRandomization::mr_egger (MR_obj, robust = FALSE, distribution = "normal", penalized = FALSE, alpha = 0.05, correl = FALSE)

fullres[myrow,1] <- "MR-EGGER"

fullres[myrow,2] <- mystats

fullres[myrow,3] <- MR_egg$SNPs

fullres[myrow,4] <- sprintf("%.3f", exp(MR_egg$Estimate))

fullres[myrow,5] <- sprintf("%.3f", exp(MR_egg$CILower.Est))

fullres[myrow,6] <- sprintf("%.3f", exp(MR_egg$CIUpper.Est))

fullres[myrow,7] <- sprintf("%.3f", MR_egg$Estimate)

fullres[myrow,8] <- sprintf("%.3f", MR_egg$StdError.Est)

fullres[myrow,9] <- sprintf("%.2e", MR_egg$Pvalue.Est)

fullres[myrow,10] <- sprintf("%.3f", MR_egg$Heter.Stat[1])

fullres[myrow,11] <- sprintf("%.3f", MR_egg$Heter.Stat[2])

fullres[myrow,12] <- sprintf("%.3f", MR_egg$Intercept)

fullres[myrow,13] <- sprintf("%.2e", MR_egg$Pvalue.Int)

fullres[myrow,14] <- "-"

myrow <- myrow + 1

MR_median <- MendelianRandomization::mr_median (MR_obj, distribution = "normal", weighting = "weighted", alpha = 0.05)

fullres[myrow,1] <- "MR-WMEDIAN"

fullres[myrow,2] <- mystats

fullres[myrow,3] <- MR_median$SNPs

fullres[myrow,4] <- sprintf("%.3f", exp(MR_median$Estimate))

fullres[myrow,5] <- sprintf("%.3f", exp(MR_median$CILower))

fullres[myrow,6] <- sprintf("%.3f", exp(MR_median$CIUpper))

fullres[myrow,7] <- sprintf("%.3f", MR_median$Estimate)

fullres[myrow,8] <- sprintf("%.3f", MR_median$StdError)

fullres[myrow,9] <- sprintf("%.2e", MR_median$Pvalue)

fullres[myrow,10:14] <- c("-","-","-","-","-")

myrow <- myrow + 1

MR_mbe <- MendelianRandomization::mr_mbe (MR_obj, distribution = "normal", weighting = "weighted", stderror = "simple", phi = 1, alpha = 0.05)

fullres[myrow,1] <- "MR-MBE"

fullres[myrow,2] <- mystats

fullres[myrow,3] <- MR_mbe$SNPs

fullres[myrow,4] <- sprintf("%.3f", exp(MR_mbe$Estimate))

fullres[myrow,5] <- sprintf("%.3f", exp(MR_mbe$CILower))

fullres[myrow,6] <- sprintf("%.3f", exp(MR_mbe$CIUpper))

fullres[myrow,7] <- sprintf("%.3f", MR_mbe$Estimate)

fullres[myrow,8] <- sprintf("%.3f", MR_mbe$StdError)

fullres[myrow,9] <- sprintf("%.2e", MR_mbe$Pvalue)

fullres[myrow,10:14] <- c("-","-","-","-","-")

myrow <- myrow + 1

if(!skip_mr_presso){

MR_mrp <- mr_presso(BetaOutcome = "BETA_OUT", BetaExposure = "BETA_OKBAY", SdOutcome = "SE_OUT", SdExposure = "SE_OKBAY",

OUTLIERtest = TRUE, DISTORTIONtest = TRUE, NbDistribution = 1000, SignifThreshold = 0.05, data=dataMR)

num_ouliers <- sum(MR_mrp$MR$Outlier[,2]<0.05)

h <- ifelse(num_ouliers>0, 2, 1)

LCI <- MR_mrp[[1]]$Causal[h] - (1.96*MR_mrp[[1]]$Sd[h])

UCI <- MR_mrp[[1]]$Causal[h] + (1.96*MR_mrp[[1]]$Sd[h])

fullres[myrow,1] <- "MR-PRESSO"

fullres[myrow,2] <- mystats

fullres[myrow,3] <- MR_ivw$SNPs - num_ouliers

fullres[myrow,4] <- sprintf("%.3f", exp(MR_mrp[[1]]$Causal[h]))

fullres[myrow,5] <- sprintf("%.3f", exp(LCI))

fullres[myrow,6] <- sprintf("%.3f", exp(UCI))

fullres[myrow,7] <- sprintf("%.3f", MR_mrp[[1]]$Causal[h])

fullres[myrow,8] <- sprintf("%.3f", MR_mrp[[1]]$Sd[h])

fullres[myrow,9] <- sprintf("%.2e", MR_mrp[[1]]$P[h])

fullres[myrow,10:13] <- c("-","-","-","-")

fullres[myrow,14] <- num_ouliers

myrow <- myrow + 1

}

}

fullres

ivw_res <- fullres[which(fullres$Method=="IVW-MR"),]

ivw_res$ci95 <- paste0(ivw_res$LCI95, " to ", ivw_res$UCI95)

write.csv(fullres, file=tableS2_file, row.names=FALSE)

write.csv(ivw_res[,c("Sumstats","OR","ci95","BETA","SE","P")], file=table2_file, row.names=FALSE)

write.csv(dataMR[,c("SNP","CHR","POS_GRCh37","EA","BETA_OKBAY","SE_OKBAY","P_OKBAY")],file=tableS1_file, row.names=FALSE)

# Save list of included SNPs

write.table(dataMR$SNP, file=final_snplist_file, col.names=FALSE, row.names=FALSE, quote=FALSE)

####

# Files

# -----

poag_sumstats_file <- paste0(mydir, "clumped/poag_sumstats_for_clumped_vars_2025-05-20.txt")

merged_sumstats_file <- paste0(mydir, "poag_merged_sumstats_2025-05-20.txt")

tableS3_file <- paste0(mydir, "Manuscript/TableS3_2025-05-25.csv")

table3_file <- paste0(mydir, "Manuscript/Table3_2025-05-25.csv")

clumped_zhou_file <- paste0(mydir, "clumped/zhou_2025-05-20_clumped.out")

clumped_jiang_file <- paste0(mydir, "clumped/jiang_2025-05-20_clumped.out")

clumped_neale_file <- paste0(mydir, "clumped/neale419_2025-05-20_clumped.out")

clumped_mrcieu_file <- paste0(mydir, "clumped/mrcieu6353_2025-05-20_clumped.out")

clumped_finngenR9_file <- paste0(mydir, "clumped/finngen_r9_2025-05-20_clumped.out")

clumped_finngenR10_file <- paste0(mydir, "clumped/finngen_r10_2025-05-20_clumped.out")

clumped_finngenR11_file <- paste0(mydir, "clumped/finngen_r11_2025-05-20_clumped.out")

clumped_new_plink_file <- paste0(mydir, "clumped/plink_new_2025-05-20_clumped.out")

clumped_new_saige_file <- paste0(mydir, "clumped/saige_new_2025-05-20_clumped.out")

# Parameters

# ----------

skip_mr_presso <- FALSE

###############################

# HARMONIZE SUMSTATS

# ------------------

# SAIGE/GATE

dataPOAG <- as.data.frame(fread(file=poag_sumstats_file, header=TRUE))

dataSAIGE <- as.data.frame(fread(file=clumped_new_saige_file, header=TRUE))

names(dataSAIGE) <- c("SNP","CHR","POS_SAIGE","EA_SAIGE","NEA_SAIGE","BETA_Temp","SE_SAIGE","P_SAIGE")

names(dataPOAG) <- c("CHR","POS","SNP","EA_POAG","NEA_POAG","BETA_POAG","SE_POAG","P_POAG")

dataM <- merge(dataPOAG,dataSAIGE, by=c("SNP","CHR"), all=TRUE)

dataM$BETA_SAIGE <- ifelse(dataM$EA_POAG==dataM$EA_SAIGE, dataM$BETA_Temp, NA)

dataM$BETA_SAIGE <- ifelse(dataM$EA_POAG==dataM$NEA_SAIGE, -1*dataM$BETA_Temp, dataM$BETA_SAIGE)

dataM$NEA_SAIGE <- NULL

dataM$EA_SAIGE <- NULL

dataM$BETA_Temp <- NULL

dataM$POS_SAIGE <- NULL

write.csv(dataM, file=merged_sumstats_file, row.names=FALSE)

# PLINK

dataPOAG <- as.data.frame(fread(file=merged_sumstats_file, header=TRUE))

dataPLINK <- as.data.frame(fread(file=clumped_new_plink_file, header=TRUE))

names(dataPLINK) <- c("SNP","CHR","POS_PLINK","EA_PLINK","NEA_PLINK","BETA_Temp","SE_PLINK","P_PLINK")

dataM <- merge(dataPOAG,dataPLINK, by=c("SNP","CHR"), all=TRUE)

dataM$BETA_PLINK <- ifelse(dataM$EA_POAG==dataM$EA_PLINK, dataM$BETA_Temp, NA)

dataM$BETA_PLINK <- ifelse(dataM$EA_POAG==dataM$NEA_PLINK, -1*dataM$BETA_Temp, dataM$BETA_PLINK)

dataM$NEA_PLINK <- NULL

dataM$EA_PLINK <- NULL

dataM$BETA_Temp <- NULL

dataM$POS_PLINK <- NULL

write.csv(dataM, file=merged_sumstats_file, row.names=FALSE)

# ZHOU

dataPOAG <- as.data.frame(fread(file=merged_sumstats_file, header=TRUE))

dataZHOU <- as.data.frame(fread(file=clumped_zhou_file, header=TRUE))

names(dataZHOU) <- c("SNP","CHR","POS_ZHOU","EA_ZHOU","NEA_ZHOU","BETA_Temp","SE_ZHOU","P_ZHOU")

dataM <- merge(dataPOAG,dataZHOU, by=c("SNP","CHR"), all=TRUE)

dataM$BETA_ZHOU <- ifelse(dataM$EA_POAG==dataM$EA_ZHOU, dataM$BETA_Temp, NA)

dataM$BETA_ZHOU <- ifelse(dataM$EA_POAG==dataM$NEA_ZHOU, -1*dataM$BETA_Temp, dataM$BETA_ZHOU)

dataM$NEA_ZHOU <- NULL

dataM$EA_ZHOU <- NULL

dataM$BETA_Temp <- NULL

dataM$POS_ZHOU <- NULL

write.csv(dataM, file=merged_sumstats_file, row.names=FALSE)

# JIANG

dataPOAG <- as.data.frame(fread(file=merged_sumstats_file, header=TRUE))

dataJIANG <- as.data.frame(fread(file=clumped_jiang_file, header=TRUE))

names(dataJIANG) <- c("SNP","CHR","POS_JIANG","EA_JIANG","NEA_JIANG","BETA_Temp","SE_JIANG","P_JIANG")

dataM <- merge(dataPOAG,dataJIANG, by=c("SNP","CHR"), all=TRUE)

dataM$BETA_JIANG <- ifelse(dataM$EA_POAG==dataM$EA_JIANG, dataM$BETA_Temp, NA)

dataM$BETA_JIANG <- ifelse(dataM$EA_POAG==dataM$NEA_JIANG, -1*dataM$BETA_Temp, dataM$BETA_JIANG)

dataM$NEA_JIANG <- NULL

dataM$EA_JIANG <- NULL

dataM$BETA_Temp <- NULL

dataM$POS_JIANG <- NULL

write.csv(dataM, file=merged_sumstats_file, row.names=FALSE)

# MRCIEU

dataPOAG <- as.data.frame(fread(file=merged_sumstats_file, header=TRUE))

dataMRCIEU <- as.data.frame(fread(file=clumped_mrcieu_file, header=TRUE))

names(dataMRCIEU) <- c("SNP","CHR","POS_MRCIEU","EA_MRCIEU","NEA_MRCIEU","BETA_Temp","SE_MRCIEU","P_MRCIEU")

dataM <- merge(dataPOAG,dataMRCIEU, by=c("SNP","CHR"), all=TRUE)

dataM$BETA_MRCIEU <- ifelse(dataM$EA_POAG==dataM$EA_MRCIEU, dataM$BETA_Temp, NA)

dataM$BETA_MRCIEU <- ifelse(dataM$EA_POAG==dataM$NEA_MRCIEU, -1*dataM$BETA_Temp, dataM$BETA_MRCIEU)

dataM$NEA_MRCIEU <- NULL

dataM$EA_MRCIEU <- NULL

dataM$BETA_Temp <- NULL

dataM$POS_MRCIEU <- NULL

write.csv(dataM, file=merged_sumstats_file, row.names=FALSE)

# NEALE

dataPOAG <- as.data.frame(fread(file=merged_sumstats_file, header=TRUE))

dataNEALE <- as.data.frame(fread(file=clumped_neale_file, header=TRUE))

names(dataNEALE) <- c("SNP","CHR","POS_NEALE","EA_NEALE","NEA_NEALE","BETA_Temp","SE_NEALE","P_NEALE")

dataM <- merge(dataPOAG,dataNEALE, by=c("SNP","CHR"), all=TRUE)

dataM$BETA_NEALE <- ifelse(dataM$EA_POAG==dataM$EA_NEALE, dataM$BETA_Temp, NA)

dataM$BETA_NEALE <- ifelse(dataM$EA_POAG==dataM$NEA_NEALE, -1*dataM$BETA_Temp, dataM$BETA_NEALE)

dataM$NEA_NEALE <- NULL

dataM$EA_NEALE <- NULL

dataM$BETA_Temp <- NULL

dataM$POS_NEALE <- NULL

write.csv(dataM, file=merged_sumstats_file, row.names=FALSE)

# FinnGenR9

dataPOAG <- as.data.frame(fread(file=merged_sumstats_file, header=TRUE))

dataFinnGenR9 <- as.data.frame(fread(file=clumped_finngenR9_file, header=TRUE))

names(dataFinnGenR9) <- c("SNP","CHR","POS_FinnGenR9","EA_FinnGenR9","NEA_FinnGenR9","BETA_Temp","SE_FinnGenR9","P_FinnGenR9")

dataM <- merge(dataPOAG,dataFinnGenR9, by=c("SNP","CHR"), all=TRUE)

dataM$BETA_FinnGenR9 <- ifelse(dataM$EA_POAG==dataM$EA_FinnGenR9, dataM$BETA_Temp, NA)

dataM$BETA_FinnGenR9 <- ifelse(dataM$EA_POAG==dataM$NEA_FinnGenR9, -1*dataM$BETA_Temp, dataM$BETA_FinnGenR9)

dataM$NEA_FinnGenR9 <- NULL

dataM$EA_FinnGenR9 <- NULL

dataM$BETA_Temp <- NULL

dataM$POS_FinnGenR9 <- NULL

write.csv(dataM, file=merged_sumstats_file, row.names=FALSE)

# FinnGenR10

dataPOAG <- as.data.frame(fread(file=merged_sumstats_file, header=TRUE))

dataFinnGenR10 <- as.data.frame(fread(file=clumped_finngenR10_file, header=TRUE))

names(dataFinnGenR10) <- c("SNP","CHR","POS_FinnGenR10","EA_FinnGenR10","NEA_FinnGenR10","BETA_Temp","SE_FinnGenR10","P_FinnGenR10")

dataM <- merge(dataPOAG,dataFinnGenR10, by=c("SNP","CHR"), all=TRUE)

dataM$BETA_FinnGenR10 <- ifelse(dataM$EA_POAG==dataM$EA_FinnGenR10, dataM$BETA_Temp, NA)

dataM$BETA_FinnGenR10 <- ifelse(dataM$EA_POAG==dataM$NEA_FinnGenR10, -1*dataM$BETA_Temp, dataM$BETA_FinnGenR10)

dataM$NEA_FinnGenR10 <- NULL

dataM$EA_FinnGenR10 <- NULL

dataM$BETA_Temp <- NULL

dataM$POS_FinnGenR10 <- NULL

write.csv(dataM, file=merged_sumstats_file, row.names=FALSE)

# FinnGenR11

dataPOAG <- as.data.frame(fread(file=merged_sumstats_file, header=TRUE))

dataFinnGenR11 <- as.data.frame(fread(file=clumped_finngenR11_file, header=TRUE))

names(dataFinnGenR11) <- c("SNP","CHR","POS_FinnGenR11","EA_FinnGenR11","NEA_FinnGenR11","BETA_Temp","SE_FinnGenR11","P_FinnGenR11")

dataM <- merge(dataPOAG,dataFinnGenR11, by=c("SNP","CHR"), all=TRUE)

dataM$BETA_FinnGenR11 <- ifelse(dataM$EA_POAG==dataM$EA_FinnGenR11, dataM$BETA_Temp, NA)

dataM$BETA_FinnGenR11 <- ifelse(dataM$EA_POAG==dataM$NEA_FinnGenR11, -1*dataM$BETA_Temp, dataM$BETA_FinnGenR11)

dataM$NEA_FinnGenR11 <- NULL

dataM$EA_FinnGenR11 <- NULL

dataM$BETA_Temp <- NULL

dataM$POS_FinnGenR11 <- NULL

write.csv(dataM, file=merged_sumstats_file, row.names=FALSE)

###############################

# MENDELIAN RANDOMIZATION

# -----------------------

dataM <- read.csv(file=merged_sumstats_file, header=TRUE)

# Create a table for the results

fullres <- as.data.frame(matrix(ncol=14, nrow=2))

names(fullres) <- c("Method","Sumstats","Num_IVs","OR","LCI95","UCI95","BETA","SE","P","Qstat", "Qpval","Egger_intercept","Egger_P","MRP_outliers")

myrow <- 1

# Run MR

sumstats_list <- c("JIANG","ZHOU","NEALE","MRCIEU","FinnGenR9","FinnGenR10","FinnGenR11","SAIGE","PLINK")

for(k in 1:length(sumstats_list)){

mystats <- sumstats_list[k]

mybeta <- paste0("BETA_",mystats)

myse <- paste0("SE_",mystats)

dataMR <- dataM[which(!is.na(dataM[,mybeta])),]

dataMR$BETA_EXP <- dataMR[,mybeta]

dataMR$SE_EXP <- dataMR[,myse]

MR_obj <- MendelianRandomization::mr_input(exposure = "Myopia status",

outcome = "POAG status",

snps = dataMR$SNP,

bx = dataMR[,mybeta],

bxse = dataMR[,myse],

by = dataMR$BETA_POAG,

byse = dataMR$SE_POAG)

MR_ivw <- MendelianRandomization::mr_ivw (MR_obj, robust = FALSE, distribution = "normal", penalized = FALSE, alpha = 0.05, correl = FALSE)

fullres[myrow,1] <- "IVW-MR"

fullres[myrow,2] <- mystats

fullres[myrow,3] <- MR_ivw$SNPs

fullres[myrow,4] <- sprintf("%.3f", exp(MR_ivw$Estimate))

fullres[myrow,5] <- sprintf("%.3f", exp(MR_ivw$CILower))

fullres[myrow,6] <- sprintf("%.3f", exp(MR_ivw$CIUpper))

fullres[myrow,7] <- sprintf("%.3f", MR_ivw$Estimate)

fullres[myrow,8] <- sprintf("%.3f", MR_ivw$StdError)

fullres[myrow,9] <- sprintf("%.2e", MR_ivw$Pvalue)

fullres[myrow,10] <- sprintf("%.3f", MR_ivw$Heter.Stat[1])

fullres[myrow,11] <- sprintf("%.3f", MR_ivw$Heter.Stat[2])

fullres[myrow,12:14] <- c("-","-","-")

myrow <- myrow + 1

fullres[myrow,1] <- "MR-EGGER"

if(nrow(dataMR)>1){

MR_egg <- MendelianRandomization::mr_egger (MR_obj, robust = FALSE, distribution = "normal", penalized = FALSE, alpha = 0.05, correl = FALSE)

fullres[myrow,2] <- mystats

fullres[myrow,3] <- MR_egg$SNPs

fullres[myrow,4] <- sprintf("%.3f", exp(MR_egg$Estimate))

fullres[myrow,5] <- sprintf("%.3f", exp(MR_egg$CILower.Est))

fullres[myrow,6] <- sprintf("%.3f", exp(MR_egg$CIUpper.Est))

fullres[myrow,7] <- sprintf("%.3f", MR_egg$Estimate)

fullres[myrow,8] <- sprintf("%.3f", MR_egg$StdError.Est)

fullres[myrow,9] <- sprintf("%.2e", MR_egg$Pvalue.Est)

fullres[myrow,10] <- sprintf("%.3f", MR_egg$Heter.Stat[1])

fullres[myrow,11] <- sprintf("%.3f", MR_egg$Heter.Stat[2])

fullres[myrow,12] <- sprintf("%.3f", MR_egg$Intercept)

fullres[myrow,13] <- sprintf("%.2e", MR_egg$Pvalue.Int)

fullres[myrow,14] <- "-"

}

myrow <- myrow + 1

fullres[myrow,1] <- "MR-WMEDIAN"

MR_median <- MendelianRandomization::mr_median (MR_obj, distribution = "normal", weighting = "weighted", alpha = 0.05)

if(nrow(dataMR)>1){

fullres[myrow,2] <- mystats

fullres[myrow,3] <- MR_median$SNPs

fullres[myrow,4] <- sprintf("%.3f", exp(MR_median$Estimate))

fullres[myrow,5] <- sprintf("%.3f", exp(MR_median$CILower))

fullres[myrow,6] <- sprintf("%.3f", exp(MR_median$CIUpper))

fullres[myrow,7] <- sprintf("%.3f", MR_median$Estimate)

fullres[myrow,8] <- sprintf("%.3f", MR_median$StdError)

fullres[myrow,9] <- sprintf("%.2e", MR_median$Pvalue)

fullres[myrow,10:14] <- c("-","-","-","-","-")

}

myrow <- myrow + 1

fullres[myrow,1] <- "MR-MBE"

if(nrow(dataMR)>1){

MR_mbe <- MendelianRandomization::mr_mbe (MR_obj, distribution = "normal", weighting = "weighted", stderror = "simple", phi = 1, alpha = 0.05)

fullres[myrow,2] <- mystats

fullres[myrow,3] <- MR_mbe$SNPs

fullres[myrow,4] <- sprintf("%.3f", exp(MR_mbe$Estimate))

fullres[myrow,5] <- sprintf("%.3f", exp(MR_mbe$CILower))

fullres[myrow,6] <- sprintf("%.3f", exp(MR_mbe$CIUpper))

fullres[myrow,7] <- sprintf("%.3f", MR_mbe$Estimate)

fullres[myrow,8] <- sprintf("%.3f", MR_mbe$StdError)

fullres[myrow,9] <- sprintf("%.2e", MR_mbe$Pvalue)

fullres[myrow,10:14] <- c("-","-","-","-","-")

}

myrow <- myrow + 1

if(!skip_mr_presso){

if(nrow(dataMR)>1){

MR_mrp <- mr_presso(BetaOutcome = "BETA_POAG", BetaExposure = "BETA_EXP", SdOutcome = "SE_POAG", SdExposure = "SE_EXP",

OUTLIERtest = TRUE, DISTORTIONtest = TRUE, NbDistribution = 1000, SignifThreshold = 0.05, data=dataMR)

num_ouliers <- sum(MR_mrp$MR$Outlier[,2]<0.05)

h <- ifelse(num_ouliers>0, 2, 1)

LCI <- MR_mrp[[1]]$Causal[h] - (1.96*MR_mrp[[1]]$Sd[h])

UCI <- MR_mrp[[1]]$Causal[h] + (1.96*MR_mrp[[1]]$Sd[h])

fullres[myrow,1] <- "MR-PRESSO"

fullres[myrow,2] <- mystats

fullres[myrow,3] <- MR_ivw$SNPs - num_ouliers

fullres[myrow,4] <- sprintf("%.3f", exp(MR_mrp[[1]]$Causal[h]))

fullres[myrow,5] <- sprintf("%.3f", exp(LCI))

fullres[myrow,6] <- sprintf("%.3f", exp(UCI))

fullres[myrow,7] <- sprintf("%.3f", MR_mrp[[1]]$Causal[h])

fullres[myrow,8] <- sprintf("%.3f", MR_mrp[[1]]$Sd[h])

fullres[myrow,9] <- sprintf("%.2e", MR_mrp[[1]]$P[h])

fullres[myrow,10:13] <- c("-","-","-","-")

fullres[myrow,14] <- num_ouliers

myrow <- myrow + 1

}

}

}

fullres

ivw_res <- fullres[which(fullres$Method=="IVW-MR"),]

ivw_res$ci95 <- paste0(ivw_res$LCI95, " to ", ivw_res$UCI95)

write.csv(fullres, file=tableS3_file, row.names=FALSE)

write.csv(ivw_res[,c("Sumstats","Num_IVs","OR","ci95","BETA","SE","P")], file=table3_file, row.names=FALSE)

##########################

# **Supplementary Note S8. Code to reproduce R^2^, R^2^_l_, and F-statistic calculations**

# Function to generate 2x2 genotype contingency table given EAF, prop, OR

contingency <- function(EAF, prop, odds_ratio, eps = 1e-15) {

a <- odds_ratio - 1

b <- (EAF + prop) * (1 - odds_ratio) - 1

c_ <- odds_ratio * EAF * prop

if (abs(a) < eps) {

z <- -c_ / b

} else {

d <- b^2 - 4 * a * c_

if (d < eps^2) {

s <- 0

} else {

s <- c(-1, 1)

}

z <- (-b + s * sqrt(max(0, d))) / (2 * a)

}

y <- vapply(z, function(a) {

zapsmall(matrix(c(a, prop - a, EAF - a, 1 + a - EAF - prop), 2, 2))

}, matrix(0.0, 2, 2))

i <- apply(y, 3, function(u) all(u >= 0))

return(y[, , i])

}

# Function to estimate population allele frequency accounting for case/control status and prevalence

get_population_allele_frequency <- function(EAF, odds_ratio, prop, prevalence) {

stopifnot(length(EAF) == length(odds_ratio))

EAF_out <- rep(NA_real_, length(EAF))

for (i in seq_along(odds_ratio)) {

co_all <- tryCatch(contingency(EAF[i], prop, odds_ratio[i]), error = function(e) return(NULL))

if (is.null(co_all) || length(dim(co_all)) == 0) next

co <- if (length(dim(co_all)) == 3) co_all[, , 1] else co_all

EAF_controls <- co[1, 2] / (co[1, 2] + co[2, 2])

EAF_cases <- co[1, 1] / (co[1, 1] + co[2, 1])

EAF_out[i] <- EAF_controls * (1 - prevalence) + EAF_cases * prevalence

}

return(EAF_out)

}

# Set overall myopia population prevalence (used for liability-scale conversion)

prevalence <- 0.306

# Dataset identifiers and their corresponding case proportions

steiger_sumstats_list <- c("plink", "saige", "r5", "r9", "r10", "r11", "Zhou", "Jiang")

prop_list <- c(0.386, 0.382, 0.008, 0.01, 0.01, 0.0108, 0.003, 0.08)

dfs <- list(data_edu_plink, data_edu_saige,

data_edu_r5, data_edu_r9, data_edu_r10, data_edu_r11, data_edu_zhou, data_edu_Jiang)

# Loop to compute liability-scale R² for each dataset

for (k in seq_along(steiger_sumstats_list)) {

mystats <- steiger_sumstats_list[k]

df <- dfs[[k]]

eaf_col <- paste0("EAF_", mystats)

beta_col <- paste0("BETA_", mystats)

if (!(eaf_col %in% names(df)) || !(beta_col %in% names(df))) {

warning(paste("Missing EAF or BETA column for", mystats))

next

}

EAF <- df[[eaf_col]]

BETA <- df[[beta_col]]

OR <- exp(BETA)

# Estimate population allele frequencies

popEAF <- get_population_allele_frequency(EAF, OR, prop_list[k], prevalence)

cat(mystats, " - valid popEAF:", sum(!is.na(popEAF)), "/", length(popEAF), "\n")

# Remove extreme or invalid EAFs

popEAF[popEAF < 0.001 | popEAF > 0.999] <- NA

# Liability-scale R² calculation

vg <- rep(NA_real_, length(BETA))

valid_idx <- !is.na(popEAF)

vg[valid_idx] <- BETA[valid_idx]^2 * popEAF[valid_idx] * (1 - popEAF[valid_idx])

ve <- pi^2 / 3

rsq <- vg / (vg + ve)

# Report how many R² were calculated

cat(mystats, " - rsq non-NA:", sum(!is.na(rsq)), "/", length(rsq), "\n")

# Save R² into dataframe and update list

df[[paste0("rsq_", mystats)]] <- rsq

dfs[[k]] <- df

}

# Reassign updated dataframes back to original variables

data_edu_plink <- dfs[[1]]

data_edu_saige <- dfs[[2]]

data_edu_r5 <- dfs[[3]]

data_edu_r9 <- dfs[[4]]

data_edu_r10 <- dfs[[5]]

data_edu_r11 <- dfs[[6]]

data_edu_zhou <- dfs[[7]]

data_edu_Jiang <- dfs[[8]]

data_edu_plink$Rsq_okbay <- (data_edu_plink$BETA_okbay)^2 / ((data_edu_plink$BETA_okbay)^2 + (data_edu_plink$SE_okbay)^2 * 293723)

data_edu_saige$Rsq_okbay <- (data_edu_saige$BETA_okbay)^2 / ((data_edu_saige$BETA_okbay)^2 + (data_edu_saige$SE_okbay)^2 * 293723)

data_edu_ukba419$Rsq_okbay <- (data_edu_ukba419$BETA_okbay)^2 / ((data_edu_ukba419$BETA_okbay)^2 + (data_edu_ukba419$SE_okbay)^2 * 293723)

data_edu_ukbb6353$Rsq_okbay <- (data_edu_ukbb6353$BETA_okbay)^2 / ((data_edu_ukbb6353$BETA_okbay)^2 + (data_edu_ukbb6353$SE_okbay)^2 * 293723)

data_edu_r5$Rsq_okbay <- (data_edu_r5$BETA_okbay)^2 / ((data_edu_r5$BETA_okbay)^2 + (data_edu_r5$SE_okbay)^2 * 293723)

data_edu_r9$Rsq_okbay <- (data_edu_r9$BETA_okbay)^2 / ((data_edu_r9$BETA_okbay)^2 + (data_edu_r9$SE_okbay)^2 * 293723)

data_edu_r10$Rsq_okbay <- (data_edu_r10$BETA_okbay)^2 / ((data_edu_r10$BETA_okbay)^2 + (data_edu_r10$SE_okbay)^2 * 293723)

data_edu_r11$Rsq_okbay <- (data_edu_r11$BETA_okbay)^2 / ((data_edu_r11$BETA_okbay)^2 + (data_edu_r11$SE_okbay)^2 * 293723)

data_edu_zhou$Rsq_okbay <- (data_edu_zhou$BETA_okbay)^2 / ((data_edu_zhou$BETA_okbay)^2 + (data_edu_zhou$SE_okbay)^2 * 293723)

data_edu_Jiang$Rsq_okbay <- (data_edu_Jiang$BETA_okbay)^2 / ((data_edu_Jiang$BETA_okbay)^2 + (data_edu_Jiang$SE_okbay)^2 * 293723)

#myopia GWAS in ukb-a-419 and ukb-b-6353 were based on linear scale

data_edu_ukba419$rsq_419 <- (data_edu_ukba419$BETA_419)^2 / ((data_edu_ukba419$BETA_419)^2 + (data_edu_ukba419$SE_419)^2 * 335700)

data_edu_ukbb6353$rsq_6353 <- (data_edu_ukbb6353$BETA_6353)^2 / ((data_edu_ukbb6353$BETA_6353)^2 + (data_edu_ukbb6353$SE_6353)^2 * 460536)

#################compute rsq libality for myopia in each dataset ###############################

# Set overall myopia population prevalence (used for liability-scale conversion)

prevalence_myopia <- 0.306

# Dataset identifiers and their corresponding case proportions

rsq_liability_sumstats_list <- c("plink", "saige", "r9", "r10", "r11", "Zhou", "Jiang")

prop_list <- c(0.386, 0.382, 0.01, 0.01, 0.0108, 0.003, 0.08)

dfs <- list(data_plink_glaucoma, data_saige_glaucoma,

data_r9_glaucoma, data_r10_glaucoma, data_r11_glaucoma,

data_Zhou_glaucoma, data_Jiang_glaucoma)

# Loop to compute liability-scale R² for myopia for each dataset

for (k in seq_along(rsq_liability_sumstats_list)) {

mystats <- rsq_liability_sumstats_list[k]

df <- dfs[[k]]

eaf_col <- paste0("EAF_", mystats)

beta_col <- paste0("BETA_", mystats)

if (!(eaf_col %in% names(df)) || !(beta_col %in% names(df))) {

warning(paste("Missing EAF or BETA column for", mystats))

next

}

EAF <- df[[eaf_col]]

BETA <- df[[beta_col]]

OR <- exp(BETA)

# Estimate population allele frequencies

popEAF <- get_population_allele_frequency(EAF, OR, prop_list[k], prevalence_myopia)

cat(mystats, " - valid popEAF:", sum(!is.na(popEAF)), "/", length(popEAF), "\n")

# Remove extreme or invalid EAFs

popEAF[popEAF < 0.001 | popEAF > 0.999] <- NA

# Liability-scale R² calculation

vg <- rep(NA_real_, length(BETA))

valid_idx <- !is.na(popEAF)

vg[valid_idx] <- BETA[valid_idx]^2 * popEAF[valid_idx] * (1 - popEAF[valid_idx])

ve <- pi^2 / 3

rsq <- vg / (vg + ve)

# Report how many R² were calculated

cat(mystats, " - rsq non-NA:", sum(!is.na(rsq)), "/", length(rsq), "\n")

# Save R² into dataframe and update list

df[[paste0("rsq_", mystats)]] <- rsq

df[[paste0("popEAF_", mystats)]] <- popEAF

dfs[[k]] <- df

}

# Reassign updated dataframes back to original variables

data_plink_glaucoma <- dfs[[1]]

data_saige_glaucoma <- dfs[[2]]

data_r9_glaucoma <- dfs[[3]]

data_r10_glaucoma <- dfs[[4]]

data_r11_glaucoma <- dfs[[5]]

data_Zhou_glaucoma <- dfs[[6]]

data_Jiang_glaucoma <- dfs[[7]]

#myopia GWAS in ukb-a-419 and ukb-b-6353 were based on linear scale

data_419_glaucoma$rsq_419 <- (data_419_glaucoma$BETA_419)^2 / ((data_419_glaucoma$BETA_419)^2 + (data_419_glaucoma$SE_419)^2 * 335700)

data_6353_glaucoma$rsq_6353 <- (data_6353_glaucoma$BETA_6353)^2 / ((data_6353_glaucoma$BETA_6353)^2 + (data_6353_glaucoma$SE_6353)^2 * 460536)

data_419_glaucoma$popEAF_419 <- get_population_allele_frequency(data_419_glaucoma$EAF_419, exp(data_419_glaucoma$BETA_419), 0.08, prevalence_myopia)

data_6353_glaucoma$popEAF_6353 <- get_population_allele_frequency(data_6353_glaucoma$EAF_6353, exp(data_6353_glaucoma$BETA_6353), 0.081, prevalence_myopia)

#########################################

#Rsq glaucoma for each dataset

prevalence_glaucoma <- 0.025

rsq_glaucoma_list <- c("plink", "saige", "419", "6353", "r9", "r10", "r11", "Zhou", "Jiang")

prop_glaucoma <- c(0.077, 0.077, 0.077, 0.077,0.077, 0.077,0.077, 0.077, 0.077) #(16677 cases, 199580 controls)

dfs_rsq_glaucoma <- list(data_plink_glaucoma, data_saige_glaucoma,

data_419_glaucoma, data_6353_glaucoma,

data_r9_glaucoma, data_r10_glaucoma, data_r11_glaucoma,

data_Zhou_glaucoma, data_Jiang_glaucoma)

for (i in seq_along(rsq_glaucoma_list)) {

mystats <- rsq_glaucoma_list[i]

da <- dfs_rsq_glaucoma[[i]]

eaf_col <- "EAF_glaucoma"

beta_col <- "BETA_glaucoma"

if (!(eaf_col %in% names(da)) || !(beta_col %in% names(da))) {

warning(paste("Missing EAF or BETA column for", mystats))

next

}

EAF <- da[[eaf_col]]

BETA <- da[[beta_col]]

OR <- exp(BETA)

# Estimate population allele frequencies

popEAF <- get_population_allele_frequency(EAF, OR, prop_glaucoma[i], prevalence_glaucoma)

cat(mystats, " - valid popEAF:", sum(!is.na(popEAF)), "/", length(popEAF), "\n")

# Remove extreme or invalid EAFs

popEAF[popEAF < 0.001 | popEAF > 0.999] <- NA

# Liability-scale R² calculation

vg <- rep(NA_real_, length(BETA))

valid_idx <- !is.na(popEAF)

vg[valid_idx] <- BETA[valid_idx]^2 * popEAF[valid_idx] * (1 - popEAF[valid_idx])

ve <- pi^2 / 3

rsq <- vg / (vg + ve)

# Report how many R² were calculated

cat(mystats, " - rsq non-NA:", sum(!is.na(rsq)), "/", length(rsq), "\n")

# Save R² into dataframe and update list

da$rsq_glaucoma <- rsq

dfs_rsq_glaucoma[[i]] <- da

}

data_plink_glaucoma <- dfs_rsq_glaucoma[[1]]

data_saige_glaucoma <- dfs_rsq_glaucoma[[2]]

data_419_glaucoma <- dfs_rsq_glaucoma[[3]]

data_6353_glaucoma <- dfs_rsq_glaucoma[[4]]

data_r9_glaucoma <- dfs_rsq_glaucoma[[5]]

data_r10_glaucoma <- dfs_rsq_glaucoma[[6]]

data_r11_glaucoma <- dfs_rsq_glaucoma[[7]]

data_Zhou_glaucoma <- dfs_rsq_glaucoma[[8]]

data_Jiang_glaucoma <- dfs_rsq_glaucoma[[9]]

# **Supplementary Note S9. Code to reproduce Steiger filtering**

#effective_n: effective sample size of case-control study

effective_n <- function(ncase, ncontrol){

return(2 / (1/ncase + 1/ncontrol))

}

fisher_z_test <- function(r1, r2, n1, n2) {

z1 <- atanh(r1)

z2 <- atanh(r2)

se_diff <- sqrt(1 / (n1 - 3) + 1 / (n2 - 3))

z_score <- (z1 - z2) / se_diff

p_value <- 2 * pnorm(-abs(z_score))

return(p_value)

}

####################################################################################

# Dataset identifiers

datasets <- c("plink", "saige", "419", "6353", "r5", "r9", "r10", “r11”, "Zhou", "Jiang")

#ncase, ncontrol in each sample GWAS following the list in the dataset - respectively

n_case_list <- c(25804, 35531,26943,37362,1640,3534,4106,4732,1257,36623)

n_control_list <- c(40969,57505,308757,423174,210931,361237,394028,432955,406530,419031)

# Corresponding data frame objects

dfs <- list(data_edu_plink, data_edu_saige, data_edu_ukba419, data_edu_ukbb6353,

data_edu_r5, data_edu_r9, data_edu_r10, data_edu_r11,

data_edu_zhou, data_edu_Jiang)

for (i in seq_along(datasets)) {

tag <- datasets[i]

df <- dfs[[i]]

N_exp <- 293723

N_out <- effective_n(n_case_list[i], n_control_list[i])

beta_exp <- "BETA_okbay"

rsq_exp <- "Rsq_okbay"

beta_out <- paste0("BETA_", tag)

rsq_out <- paste0("rsq_", tag)

# Check required columns exist and are numeric

required_cols <- c(beta_exp, rsq_exp, beta_out, rsq_out)

missing_cols <- required_cols[!required_cols %in% names(df)]

if (length(missing_cols) > 0) {

warning(tag, ": Missing required columns: ", paste(missing_cols, collapse = ", "))

next

}

if (!is.numeric(df[[rsq_out]]) || !is.numeric(df[[rsq_exp]])) {

warning(tag, ": R² columns are not numeric")

next

}

# Compute signed correlations

df[[paste0("r_", tag)]] <- sign(df[[beta_out]]) * sqrt(df[[rsq_out]])

df[["r_okbay"]] <- sign(df[[beta_exp]]) * sqrt(df[[rsq_exp]])

# Fisher Z-test

df[[paste0("steiger_p_", tag)]] <- mapply(function(r1, r2) {

if (is.na(r1) || is.na(r2) || abs(r1) >= 1 || abs(r2) >= 1) return(NA)

fisher_z_test(r1, r2, n1 = N_exp, n2 = N_out)

}, df[["r_okbay"]], df[[paste0("r_", tag)]])

# Keep SNPs passing Steiger directionality

df[[paste0("keep_steiger_", tag)]] <- with(df,

get(rsq_exp) > get(rsq_out))

cat(tag, "- SNPs passing Steiger:", sum(df[[paste0("keep_steiger_", tag)]], na.rm = TRUE), "/", nrow(df), "\n")

assign(paste0("data_edu_", tag), df)

write.csv(df, file = paste0("edu_", tag, "_steiger.csv"), row.names = FALSE)

# Dataset identifiers

datasets <- c("plink", "saige", "419", "6353", "r9", "r10", "r11", "Zhou", "Jiang")

#ncase, ncontrol in each sample GWAS following the list in the dataset - respectively

n_case_list <- c(25804, 35531,26943,37362,3534,4106,4732,1257,36623)

n_control_list <- c(40969,57505,308757,423174,361237,394028,432955,406530,419031)

n_case_glaucoma <- 16677

n_control_glaucoma <- 199580

# Corresponding data frame objects

dfs <- list(data_plink_glaucoma, data_saige_glaucoma, data_419_glaucoma, data_6353_glaucoma,

data_r9_glaucoma, data_r10_glaucoma, data_r11_glaucoma, data_Zhou_glaucoma, data_Jiang_glaucoma)

for (i in seq_along(datasets)) {

tag <- datasets[i]

df <- dfs[[i]]

N_out <- effective_n(n_case_glaucoma, n_control_glaucoma)

N_exp <- effective_n(n_case_list[i], n_control_list[i])

beta_exp <- paste0("BETA_", tag)

rsq_exp <- paste0("rsq_", tag)

beta_out <- "BETA_glaucoma"

rsq_out <- "rsq_glaucoma"

df[[rsq_exp]] <- as.numeric(df[[rsq_exp]])

df[[rsq_out]] <- as.numeric(df[[rsq_out]])

# Check required columns exist and are numeric

required_cols <- c(beta_exp, rsq_exp, beta_out, rsq_out)

missing_cols <- required_cols[!required_cols %in% names(df)]

if (length(missing_cols) > 0) {

warning(tag, ": Missing required columns: ", paste(missing_cols, collapse = ", "))

next

}

if (!is.numeric(df[[rsq_out]]) || !is.numeric(df[[rsq_exp]])) {

warning(tag, ": R² columns are not numeric")

next

}

# Compute signed correlations

df[[paste0("r_", tag)]] <- sign(df[[beta_exp]]) * sqrt(df[[rsq_exp]])

df[["r_glaucoma"]] <- sign(df[[beta_out]]) * sqrt(df[[rsq_out]])

# Fisher Z-test

df[[paste0("steiger_p_", tag)]] <- mapply(function(r1, r2) {

if (is.na(r1) || is.na(r2) || abs(r1) >= 1 || abs(r2) >= 1) return(NA)

fisher_z_test(r1, r2, n1 = N_exp, n2 = N_out)

}, df[[paste0("r_", tag)]], df[["r_glaucoma"]])

# Keep SNPs passing Steiger directionality

df[[paste0("keep_steiger_", tag)]] <- df[[rsq_exp]] > df[[rsq_out]]

cat(tag, "- SNPs passing Steiger:", sum(df[[paste0("keep_steiger_", tag)]], na.rm = TRUE), "/", nrow(df), "\n")

assign(paste0("data_", tag, "_glaucoma"), df)

}

# **Supplementary Table S1. Genetic variants (N=62) from the SSGAC GWAS for EduYears** used as instrumental variables for MR.

| **SNP** | **CHR** | **POS (GRCh37)** | **Effect Allele** | **BETA** | **SE** | ***P*** |
| --- | --- | --- | --- | --- | --- | --- |
| rs301800 | 1 | 8490603 | T | 0.068 | 0.012 | 1.79e-08 |
| rs11210860 | 1 | 43982527 | A | 0.061 | 0.010 | 2.36e-10 |
| rs34305371 | 1 | 72733610 | A | 0.126 | 0.017 | 3.76e-14 |
| rs1008078 | 1 | 91189731 | T | -0.058 | 0.009 | 6.01e-10 |
| rs11588857 | 1 | 204587047 | A | 0.072 | 0.012 | 5.27e-10 |
| rs1777827 | 1 | 211613114 | A | 0.054 | 0.010 | 1.55e-08 |
| rs2992632 | 1 | 243503764 | A | 0.061 | 0.011 | 8.23e-09 |
| rs76076331 | 2 | 10977585 | T | 0.072 | 0.013 | 3.63e-08 |
| rs11689269 | 2 | 15621917 | C | 0.058 | 0.010 | 1.28e-08 |
| rs1606974 | 2 | 51873599 | A | 0.079 | 0.014 | 2.8e-08 |
| rs11690172 | 2 | 57387094 | A | 0.054 | 0.010 | 1.99e-08 |
| rs2457660 | 2 | 60757419 | T | -0.061 | 0.010 | 7.11e-10 |
| rs10496091 | 2 | 61482261 | A | -0.065 | 0.010 | 5.62e-10 |
| rs13402908 | 2 | 100333377 | T | -0.065 | 0.010 | 1.7e-11 |
| rs4851251 | 2 | 100753490 | T | -0.061 | 0.011 | 1.91e-08 |
| rs17824247 | 2 | 144152539 | T | -0.058 | 0.010 | 2.77e-09 |
| rs16845580 | 2 | 161920884 | T | 0.058 | 0.010 | 2.65e-09 |
| rs4500960 | 2 | 162818621 | T | -0.058 | 0.009 | 3.75e-10 |
| rs2245901 | 2 | 194296294 | A | -0.058 | 0.010 | 4.54e-09 |
| rs55830725 | 2 | 237056854 | A | -0.079 | 0.013 | 5.37e-10 |
| rs35761247 | 3 | 48623124 | A | 0.122 | 0.022 | 3.82e-08 |
| rs62259535 | 3 | 48939052 | A | 0.173 | 0.029 | 2.63e-09 |
| rs112634398 | 3 | 50075494 | A | 0.130 | 0.024 | 4.61e-08 |
| rs62263923 | 3 | 85674790 | A | -0.058 | 0.010 | 7.01e-09 |
| rs6799130 | 3 | 160847801 | C | -0.054 | 0.010 | 2.82e-08 |
| rs12646808 | 4 | 3249828 | T | 0.058 | 0.010 | 4e-08 |
| rs34072092 | 4 | 28801221 | T | 0.086 | 0.016 | 3.91e-08 |
| rs3101246 | 4 | 42649935 | T | -0.054 | 0.010 | 1.43e-08 |
| rs4863692 | 4 | 140764124 | T | 0.065 | 0.010 | 1.56e-10 |
| rs4493682 | 5 | 45188024 | C | 0.068 | 0.012 | 3.32e-08 |
| rs2964197 | 5 | 57535206 | T | 0.054 | 0.010 | 3.02e-08 |
| rs61160187 | 5 | 60111579 | A | -0.061 | 0.010 | 3.49e-10 |
| rs10061788 | 5 | 87934707 | A | 0.076 | 0.013 | 2.46e-09 |
| rs2431108 | 5 | 103947968 | T | 0.058 | 0.010 | 5.27e-09 |
| rs1402025 | 5 | 113987898 | T | 0.061 | 0.011 | 3.42e-08 |
| rs62379838 | 5 | 120102028 | T | 0.058 | 0.010 | 3.3e-08 |
| rs56231335 | 6 | 98187291 | T | -0.061 | 0.010 | 2.07e-09 |
| rs7767938 | 6 | 153367613 | T | 0.061 | 0.011 | 2.44e-08 |
| rs2615691 | 7 | 23402104 | A | -0.133 | 0.024 | 4.71e-08 |
| rs12531458 | 7 | 39090698 | A | 0.050 | 0.009 | 3.11e-08 |
| rs12671937 | 7 | 92654365 | A | 0.058 | 0.009 | 9.15e-10 |
| rs113520408 | 7 | 128402782 | A | 0.061 | 0.011 | 1.97e-08 |
| rs17167170 | 7 | 133302345 | A | 0.072 | 0.012 | 1.14e-09 |
| rs11768238 | 7 | 135227513 | A | -0.061 | 0.010 | 9.9e-10 |
| rs12682297 | 8 | 145712860 | A | -0.058 | 0.010 | 3.93e-09 |
| rs1871109 | 9 | 1746016 | T | -0.058 | 0.009 | 4.35e-10 |
| rs13294439 | 9 | 23358875 | A | -0.083 | 0.010 | 2.2e-17 |
| rs895606 | 9 | 88003668 | A | 0.054 | 0.010 | 2.25e-08 |
| rs7854982 | 9 | 124644562 | T | -0.054 | 0.009 | 1.29e-08 |
| rs11191193 | 10 | 103802408 | A | 0.065 | 0.010 | 5.44e-11 |
| rs12772375 | 10 | 104082688 | T | -0.054 | 0.010 | 1.56e-08 |
| rs7945718 | 11 | 12748819 | A | 0.054 | 0.010 | 1.54e-08 |
| rs7955289 | 12 | 14653667 | A | 0.061 | 0.010 | 4.49e-10 |
| rs2456973 | 12 | 56416928 | A | -0.072 | 0.010 | 1.06e-12 |
| rs7131944 | 12 | 92159557 | A | 0.054 | 0.009 | 9.02e-09 |
| rs572016 | 12 | 121279083 | A | 0.050 | 0.009 | 3.46e-08 |
| rs9537821 | 13 | 58402771 | A | 0.086 | 0.010 | 1.5e-16 |
| rs1043209 | 14 | 23373986 | A | 0.065 | 0.010 | 1.82e-11 |
| rs17119973 | 14 | 84913111 | A | -0.068 | 0.011 | 3.55e-10 |
| rs12969294 | 18 | 35186122 | A | -0.058 | 0.010 | 7.24e-09 |
| rs2837992 | 21 | 42620520 | T | 0.054 | 0.010 | 3.8e-08 |
| rs165633 | 22 | 29880773 | A | -0.065 | 0.011 | 2.86e-09 |

# Supplementary Table S2. Full MR results for analyses examining the effect of EduYears on myopia without Steiger filtering.

| Method | Sum. stats^*^ | Num. IVs | OR | LCI95 | UCI95 | BETA | SE | P | Qstat | Qpval | Egger intercept | Egger P | Num. MRP outliers |
| --- | --- | --- | --- | --- | --- | --- | --- | --- | --- | --- | --- | --- | --- |
| IVW-MR | JIANG | 62 | 1.152 | 1.097 | 1.210 | 0.141 | 0.025 | 1.63e-08 | 125.895 | 0 | - | - | - |
| MR-EGGER | JIANG | 62 | 1.010 | 0.795 | 1.284 | 0.010 | 0.122 | 9.33e-01 | 123.424 | 0 | 0.009 | 2.73e-01 | - |
| MR-WMEDIAN | JIANG | 62 | 1.174 | 1.112 | 1.240 | 0.160 | 0.028 | 8.53e-09 | - | - | - | - | - |
| MR-MBE | JIANG | 62 | 1.194 | 1.032 | 1.382 | 0.177 | 0.074 | 1.70e-02 | - | - | - | - | - |
| MR-PRESSO | JIANG | 61 | 1.162 | 1.108 | 1.219 | 0.150 | 0.024 | 5.70e-08 | - | - | - | - | 1 |
| IVW-MR | ZHOU | 62 | 1.242 | 1.043 | 1.479 | 0.217 | 0.089 | 1.49e-02 | 47.718 | 0.893 | - | - | - |
| MR-EGGER | ZHOU | 62 | 0.775 | 0.332 | 1.811 | -0.254 | 0.433 | 5.57e-01 | 46.48 | 0.900 | 0.031 | 2.66e-01 | - |
| MR-WMEDIAN | ZHOU | 62 | 1.112 | 0.868 | 1.425 | 0.106 | 0.126 | 4.01e-01 | - | - | - | - | - |
| MR-MBE | ZHOU | 62 | 0.960 | 0.521 | 1.770 | -0.040 | 0.312 | 8.97e-01 | - | - | - | - | - |
| MR-PRESSO | ZHOU | 62 | 1.242 | 1.064 | 1.450 | 0.217 | 0.079 | 7.77e-03 | - | - | - | - | 0 |
| IVW-MR | NEALE | 62 | 0.992 | 0.988 | 0.996 | -0.008 | 0.002 | 1.70e-05 | 102.659 | 0.001 | - | - | - |
| MR-EGGER | NEALE | 62 | 1.002 | 0.984 | 1.021 | 0.002 | 0.009 | 7.91e-01 | 100.343 | 0.001 | -0.001 | 2.39e-01 | - |
| MR-WMEDIAN | NEALE | 62 | 0.991 | 0.987 | 0.996 | -0.009 | 0.002 | 1.19e-04 | - | - | - | - | - |
| MR-MBE | NEALE | 62 | 0.993 | 0.981 | 1.005 | -0.007 | 0.006 | 2.40e-01 | - | - | - | - | - |
| MR-PRESSO | NEALE | 62 | 0.992 | 0.988 | 0.996 | -0.008 | 0.002 | 6.24e-05 | - | - | - | - | 0 |
| IVW-MR | NEALE_transformed^†^ | 62 | 0.894 | 0.850 | 0.941 | -0.112 | 0.026 | 1.51e-05 | 102.367 | 0.001 | - | - | - |
| MR-EGGER | NEALE_transformed^†^ | 62 | 1.019 | 0.799 | 1.299 | 0.019 | 0.124 | 8.80e-01 | 100.422 | 0.001 | -0.009 | 2.81e-01 | - |
| MR-WMEDIAN | NEALE_transformed^†^ | 62 | 0.885 | 0.834 | 0.941 | -0.122 | 0.031 | 8.00e-05 | - | - | - | - | - |
| MR-MBE | NEALE_transformed^†^ | 62 | 0.903 | 0.761 | 1.070 | -0.103 | 0.087 | 2.38e-01 | - | - | - | - | - |
| MR-PRESSO | NEALE_transformed^†^ | 61 | 0.885 | 0.843 | 0.928 | -0.122 | 0.025 | 5.47e-06 | - | - | - | - | 1 |
| IVW-MR | MRCIEU | 62 | 1.010 | 1.006 | 1.014 | 0.010 | 0.002 | 1.74e-07 | 138.608 | 0 | - | - | - |
| MR-EGGER | MRCIEU | 62 | 0.997 | 0.979 | 1.015 | -0.003 | 0.009 | 7.45e-01 | 134.003 | 0 | 0.001 | 1.51e-01 | - |
| MR-WMEDIAN | MRCIEU | 62 | 1.012 | 1.008 | 1.016 | 0.012 | 0.002 | 1.25e-08 | - | - | - | - | - |
| MR-MBE | MRCIEU | 62 | 1.015 | 1.003 | 1.026 | 0.015 | 0.006 | 1.23e-02 | - | - | - | - | - |
| MR-PRESSO | MRCIEU | 61 | 1.011 | 1.007 | 1.014 | 0.011 | 0.002 | 3.77e-07 | - | - | - | - | 1 |
| IVW-MR | MRCIEU_transformed^†^ | 62 | 1.143 | 1.087 | 1.201 | 0.133 | 0.025 | 1.63e-07 | 138.406 | 0 | - | - | - |
| MR-EGGER | MRCIEU_transformed^†^ | 62 | 0.968 | 0.763 | 1.229 | -0.032 | 0.122 | 7.92e-01 | 134.082 | 0 | 0.011 | 1.64e-01 | - |
| MR-WMEDIAN | MRCIEU_transformed^†^ | 62 | 1.172 | 1.110 | 1.237 | 0.159 | 0.028 | 9.80e-09 | - | - | - | - | - |
| MR-MBE | MRCIEU_transformed^†^ | 62 | 1.215 | 1.046 | 1.411 | 0.195 | 0.076 | 1.07e-02 | - | - | - | - | - |
| MR-PRESSO | MRCIEU_transformed^†^ | 62 | 1.143 | 1.087 | 1.201 | 0.133 | 0.025 | 2.13e-06 | - | - | - | - | 0 |
| IVW-MR | FinnGenR5 | 62 | 1.130 | 0.940 | 1.358 | 0.122 | 0.094 | 1.94e-01 | 85.041 | 0.023 | - | - | - |
| MR-EGGER | FinnGenR5 | 62 | 0.764 | 0.316 | 1.845 | -0.270 | 0.450 | 5.49e-01 | 83.933 | 0.022 | 0.026 | 3.73e-01 | - |
| MR-WMEDIAN | FinnGenR5 | 62 | 1.063 | 0.843 | 1.339 | 0.061 | 0.118 | 6.07e-01 | - | - | - | - | - |
| MR-MBE | FinnGenR5 | 62 | 1.000 | 0.559 | 1.788 | 0.000 | 0.297 | 9.99e-01 | - | - | - | - | - |
| MR-PRESSO | FinnGenR5 | 61 | 1.087 | 0.917 | 1.289 | 0.083 | 0.087 | 3.41e-01 | - | - | - | - | 1 |
| IVW-MR | FinnGenR9 | 62 | 1.073 | 0.958 | 1.202 | 0.070 | 0.058 | 2.24e-01 | 71.442 | 0.170 | - | - | - |
| MR-EGGER | FinnGenR9 | 62 | 1.254 | 0.728 | 2.161 | 0.226 | 0.278 | 4.15e-01 | 71.051 | 0.156 | -0.01 | 5.66e-01 | - |
| MR-WMEDIAN | FinnGenR9 | 62 | 1.128 | 0.968 | 1.315 | 0.120 | 0.078 | 1.24e-01 | - | - | - | - | - |
| MR-MBE | FinnGenR9 | 62 | 1.149 | 0.770 | 1.714 | 0.139 | 0.204 | 4.95e-01 | - | - | - | - | - |
| MR-PRESSO | FinnGenR9 | 62 | 1.073 | 0.958 | 1.202 | 0.070 | 0.058 | 2.29e-01 | - | - | - | - | 0 |
| IVW-MR | FinnGenR10 | 62 | 1.114 | 1.000 | 1.240 | 0.108 | 0.055 | 4.92e-02 | 74.027 | 0.122 | - | - | - |
| MR-EGGER | FinnGenR10 | 62 | 1.325 | 0.792 | 2.218 | 0.282 | 0.263 | 2.84e-01 | 73.468 | 0.114 | -0.011 | 4.99e-01 | - |
| MR-WMEDIAN | FinnGenR10 | 62 | 1.143 | 0.987 | 1.323 | 0.133 | 0.075 | 7.38e-02 | - | - | - | - | - |
| MR-MBE | FinnGenR10 | 62 | 1.244 | 0.833 | 1.859 | 0.219 | 0.205 | 2.86e-01 | - | - | - | - | - |
| MR-PRESSO | FinnGenR10 | 62 | 1.114 | 1.000 | 1.240 | 0.108 | 0.055 | 5.38e-02 | - | - | - | - | 0 |
| IVW-MR | FinnGenR11 | 62 | 1.106 | 1.001 | 1.222 | 0.101 | 0.051 | 4.75e-02 | 73.508 | 0.131 | - | - | - |
| MR-EGGER | FinnGenR11 | 62 | 1.393 | 0.865 | 2.243 | 0.331 | 0.243 | 1.73e-01 | 72.373 | 0.131 | -0.015 | 3.32e-01 | - |
| MR-WMEDIAN | FinnGenR11 | 62 | 1.145 | 1.000 | 1.312 | 0.136 | 0.069 | 5.05e-02 | - | - | - | - | - |
| MR-MBE | FinnGenR11 | 62 | 1.232 | 0.851 | 1.784 | 0.208 | 0.189 | 2.70e-01 | - | - | - | - | - |
| MR-PRESSO | FinnGenR11 | 62 | 1.106 | 1.001 | 1.222 | 0.101 | 0.051 | 5.20e-02 | - | - | - | - | 0 |
| IVW-MR | SAIGE | 62 | 1.16 | 1.091 | 1.233 | 0.148 | 0.031 | 2.15e-06 | 99.075 | 0.001 | - | - | - |
| MR-EGGER | SAIGE | 62 | 1.089 | 0.807 | 1.47 | 0.085 | 0.153 | 5.78e-01 | 98.784 | 0.001 | 0.004 | 6.74e-01 | - |
| MR-WMEDIAN | SAIGE | 62 | 1.162 | 1.077 | 1.253 | 0.15 | 0.039 | 9.83e-05 | - | - | - | - | - |
| MR-MBE | SAIGE | 62 | 1.284 | 1.018 | 1.62 | 0.25 | 0.119 | 3.51e-02 | - | - | - | - | - |
| MR-PRESSO | SAIGE | 62 | 1.16 | 1.091 | 1.233 | 0.148 | 0.031 | 1.33e-05 | - | - | - | - | 0 |
| IVW-MR | PLINK | 62 | 1.175 | 1.098 | 1.257 | 0.161 | 0.035 | 3.11e-06 | 114.679 | 0 | - | - | - |
| MR-EGGER | PLINK | 62 | 1.139 | 0.817 | 1.588 | 0.13 | 0.169 | 4.42e-01 | 114.612 | 0 | 0.002 | 8.52e-01 | - |
| MR-WMEDIAN | PLINK | 62 | 1.194 | 1.103 | 1.293 | 0.177 | 0.041 | 1.22e-05 | - | - | - | - | - |
| MR-MBE | PLINK | 62 | 1.322 | 1.045 | 1.672 | 0.279 | 0.12 | 2.00e-02 | - | - | - | - | - |
| MR-PRESSO | PLINK | 61 | 1.163 | 1.089 | 1.241 | 0.151 | 0.033 | 3.07e-05 | - | - | - | - | 1 |

^*^ Myopia GWAS summary statistics from different data repositories (JIANG refers to GCST90044326, ZHOU refers to GCST90435990 from GWAS Catalog; NEALE refers to ukb-a-419, MRC IEU to ukb-b-6353 from IEU Open GWAS; FinnGen R5/R9/R10 refer to summary statistics obtained from different FinnGen releases, respectively) and two newly performed GWAS, utilizing two methods PLINK/SAIGE, described in detail in Supplementary Note S2. All nine GWAS analyses were conducted on a binary trait for myopia.

^†^ “_transformed” refers to the transformed versions of the two myopia GWAS summary statistics conducted on linear scale (ukb-b-6353 from IEU Open GWAS and ukb-a-419 from Neale lab) (effect size of each genetic variant in the two summary statistics files was transformed from an absolute risk difference scale to a log odds ratio scale according to the MRC IEU UK Biobank GWAS pipeline, version 2, 18/01/2019)

# Supplementary Table S3. Full MR results for analyses examining the effect of EduYears on myopia with Steiger filtering.

| Method | Sum. stats^*^ | Num. IVs | OR | LCI95 | UCI95 | BETA | SE | P | Qstat | Qpval | Egger intercept | Egger P | Num.  MRP outliers |
| --- | --- | --- | --- | --- | --- | --- | --- | --- | --- | --- | --- | --- | --- |
| IVW-MR | PLINK | 61 | 1.164 | 1.090 | 1.242 | 0.152 | 0.033 | 5.44e-06 | 103.345 | 0 | - | - | - |
| MR-EGGER | PLINK | 61 | 1.185 | 0.866 | 1.623 | 0.170 | 0.160 | 2.89e-01 | 103.321 | 0 | -0.001 | 9.07e-01 | - |
| MR-WMEDIAN | PLINK | 61 | 1.194 | 1.105 | 1.292 | 0.178 | 0.040 | 8.57e-06 | - | - | - | - | - |
| MR-MBE | PLINK | 61 | 1.330 | 1.038 | 1.706 | 0.285 | 0.127 | 2.44e-02 | - | - | - | - | - |
| MR-PRESSO | PLINK | 61 | 1.164 | 1.090 | 1.242 | 0.152 | 0.033 | 2.70e-05 | - | - | - | - | 0 |
| IVW-MR | SAIGE | 62 | 1.161 | 1.092 | 1.234 | 0.149 | 0.031 | 1.73e-06 | 98.587 | 0.002 | - | - | - |
| MR-EGGER | SAIGE | 62 | 1.114 | 0.829 | 1.497 | 0.108 | 0.151 | 4.75e-01 | 98.457 | 0.001 | 0.003 | 7.79e-01 | - |
| MR-WMEDIAN | SAIGE | 62 | 1.165 | 1.080 | 1.257 | 0.153 | 0.039 | 7.64e-05 | - | - | - | - | - |
| MR-MBE | SAIGE | 62 | 1.292 | 1.018 | 1.639 | 0.256 | 0.121 | 3.51e-02 | - | - | - | - | - |
| MR-PRESSO | SAIGE | 62 | 1.161 | 1.092 | 1.234 | 0.149 | 0.031 | 1.14e-05 | - | - | - | - | 0 |
| IVW-MR | NEALE | 62 | 0.992 | 0.988 | 0.995 | -0.008 | 0.002 | 1.51e-05 | 102.367 | 0.001 | - | - | - |
| MR-EGGER | NEALE | 62 | 1.001 | 0.984 | 1.019 | 0.001 | 0.009 | 8.80e-01 | 100.422 | 0.001 | -0.001 | 2.81e-01 | - |
| MR-WMEDIAN | NEALE | 62 | 0.991 | 0.987 | 0.995 | -0.009 | 0.002 | 8.00e-05 | - | - | - | - | - |
| MR-MBE | NEALE | 62 | 0.992 | 0.980 | 1.005 | -0.008 | 0.006 | 2.38e-01 | - | - | - | - | - |
| MR-PRESSO | NEALE | 62 | 0.992 | 0.988 | 0.995 | -0.008 | 0.002 | 5.69e-05 | - | - | - | - | 0 |
| IVW-MR | NEALE_transformed^†^ | 62 | 0.894 | 0.850 | 0.941 | -0.112 | 0.026 | 1.51e-05 | 102.367 | 0.001 | - | - | - |
| MR-EGGER | NEALE_transformed^†^ | 62 | 1.019 | 0.799 | 1.299 | 0.019 | 0.124 | 8.80e-01 | 100.422 | 0.001 | -0.009 | 2.81e-01 | - |
| MR-WMEDIAN | NEALE_transformed^†^ | 62 | 0.885 | 0.834 | 0.941 | -0.122 | 0.031 | 8.00e-05 | - | - | - | - | - |
| MR-MBE | NEALE_transformed^†^ | 62 | 0.903 | 0.761 | 1.070 | -0.103 | 0.087 | 2.38e-01 | - | - | - | - | - |
| MR-PRESSO | NEALE_transformed^†^ | 61 | 0.885 | 0.843 | 0.928 | -0.122 | 0.025 | 5.47e-06 | - | - | - | - | 1 |
| IVW-MR | MRCIEU | 62 | 1.010 | 1.006 | 1.014 | 0.010 | 0.002 | 1.63e-07 | 138.406 | 0 | - | - | - |
| MR-EGGER | MRCIEU | 62 | 0.998 | 0.980 | 1.015 | -0.002 | 0.009 | 7.92e-01 | 134.082 | 0 | 0.001 | 1.64e-01 | - |
| MR-WMEDIAN | MRCIEU | 62 | 1.012 | 1.008 | 1.016 | 0.012 | 0.002 | 9.80e-09 | - | - | - | - | - |
| MR-MBE | MRCIEU | 62 | 1.015 | 1.003 | 1.026 | 0.015 | 0.006 | 1.07e-02 | - | - | - | - | - |
| MR-PRESSO | MRCIEU | 62 | 1.010 | 1.006 | 1.014 | 0.010 | 0.002 | 2.13e-06 | - | - | - | - | 0 |
| IVW-MR | MRCIEU_transformed^†^ | 62 | 1.143 | 1.087 | 1.201 | 0.133 | 0.025 | 1.63e-07 | 138.406 | 0 | - | - | - |
| MR-EGGER | MRCIEU_transformed^†^ | 62 | 0.968 | 0.763 | 1.229 | -0.032 | 0.122 | 7.92e-01 | 134.082 | 0 | 0.011 | 1.64e-01 | - |
| MR-WMEDIAN | MRCIEU_transformed^†^ | 62 | 1.172 | 1.110 | 1.237 | 0.159 | 0.028 | 9.80e-09 | - | - | - | - | - |
| MR-MBE | MRCIEU_transformed^†^ | 62 | 1.215 | 1.046 | 1.411 | 0.195 | 0.076 | 1.07e-02 | - | - | - | - | - |
| MR-PRESSO | MRCIEU_transformed^†^ | 62 | 1.143 | 1.087 | 1.201 | 0.133 | 0.025 | 2.13e-06 | - | - | - | - | 0 |
| IVW-MR | FinnGenR5 | 43 | 0.994 | 0.823 | 1.201 | -0.006 | 0.097 | 9.53e-01 | 12.119 | 1.000 | - | - | - |
| MR-EGGER | FinnGenR5 | 43 | 0.895 | 0.329 | 2.437 | -0.111 | 0.511 | 8.28e-01 | 12.075 | 1.000 | 0.007 | 8.34e-01 | - |
| MR-WMEDIAN | FinnGenR5 | 43 | 1.017 | 0.792 | 1.305 | 0.016 | 0.128 | 8.97e-01 | - | - | - | - | - |
| MR-MBE | FinnGenR5 | 43 | 1.028 | 0.624 | 1.694 | 0.027 | 0.255 | 9.14e-01 | - | - | - | - | - |
| MR-PRESSO | FinnGenR5 | 43 | 0.994 | 0.898 | 1.101 | -0.006 | 0.052 | 9.13e-01 | - | - | - | - | 0 |
| IVW-MR | FinnGenR9 | 55 | 1.088 | 0.973 | 1.216 | 0.084 | 0.057 | 1.41e-01 | 36.496 | 0.967 | - | - | - |
| MR-EGGER | FinnGenR9 | 55 | 1.420 | 0.834 | 2.420 | 0.351 | 0.272 | 1.97e-01 | 35.486 | 0.969 | -0.017 | 3.15e-01 | - |
| MR-WMEDIAN | FinnGenR9 | 55 | 1.130 | 0.965 | 1.323 | 0.122 | 0.080 | 1.29e-01 | - | - | - | - | - |
| MR-MBE | FinnGenR9 | 55 | 1.147 | 0.776 | 1.698 | 0.138 | 0.200 | 4.91e-01 | - | - | - | - | - |
| MR-PRESSO | FinnGenR9 | 55 | 1.088 | 0.992 | 1.192 | 0.084 | 0.047 | 7.91e-02 | - | - | - | - | 0 |
| IVW-MR | FinnGen10 | 57 | 1.071 | 0.967 | 1.186 | 0.069 | 0.052 | 1.87e-01 | 47.216 | 0.792 | - | - | - |
| MR-EGGER | FinnGen10 | 57 | 1.283 | 0.807 | 2.039 | 0.249 | 0.236 | 2.92e-01 | 46.603 | 0.783 | -0.012 | 4.34e-01 | - |
| MR-WMEDIAN | FinnGen10 | 57 | 1.117 | 0.963 | 1.295 | 0.111 | 0.075 | 1.43e-01 | - | - | - | - | - |
| MR-MBE | FinnGen10 | 57 | 1.243 | 0.834 | 1.854 | 0.218 | 0.204 | 2.85e-01 | - | - | - | - | - |
| MR-PRESSO | FinnGen10 | 57 | 1.071 | 0.975 | 1.176 | 0.069 | 0.048 | 1.57e-01 | - | - | - | - | 0 |
| IVW-MR | FinnGenR11 | 58 | 1.082 | 0.985 | 1.189 | 0.079 | 0.048 | 9.87e-02 | 53.303 | 0.615 | - | - | - |
| MR-EGGER | FinnGenR11 | 58 | 1.289 | 0.837 | 1.986 | 0.254 | 0.220 | 2.49e-01 | 52.640 | 0.603 | -0.011 | 4.16e-01 | - |
| MR-WMEDIAN | FinnGenR11 | 58 | 1.144 | 0.972 | 1.278 | 0.108 | 0.070 | 1.21e-01 | - | - | - | - | - |
| MR-MBE | FinnGenR11 | 58 | 1.230 | 0.850 | 1.780 | 0.207 | 0.189 | 2.73e-01 | - | - | - | - | - |
| MR-PRESSO | FinnGenR11 | 58 | 1.082 | 0.988 | 1.185 | 0.079 | 0.046 | 9.31e-02 | - | - | - | - | 0 |
| IVW-MR | ZHOU | 45 | 1.034 | 0.841 | 1.271 | 0.033 | 0.105 | 7.52e-01 | 11.043 | 1.000 | - | - | - |
| MR-EGGER | ZHOU | 45 | 0.908 | 0.345 | 2.393 | -0.096 | 0.494 | 8.45e-01 | 10.971 | 1.000 | 0.008 | 7.88e-01 | - |
| MR-WMEDIAN | ZHOU | 45 | 0.986 | 0.753 | 1.290 | -0.014 | 0.137 | 9.16e-01 | - | - | - | - | - |
| MR-MBE | ZHOU | 45 | 0.920 | 0.525 | 1.611 | -0.083 | 0.286 | 7.70e-01 | - | - | - | - | - |
| MR-PRESSO | ZHOU | 45 | 1.034 | 0.932 | 1.147 | 0.033 | 0.053 | 5.31e-01 | - | - | - | - | 0 |
| IVW-MR | JIANG | 62 | 1.152 | 1.097 | 1.210 | 0.142 | 0.025 | 1.57e-08 | 125.789 | 0 | - | - | - |
| MR-EGGER | JIANG | 62 | 1.015 | 0.801 | 1.285 | 0.015 | 0.121 | 9.03e-01 | 123.408 | 0 | 0.008 | 2.82e-01 | - |
| MR-WMEDIAN | JIANG | 62 | 1.170 | 1.108 | 1.236 | 0.157 | 0.028 | 1.93e-08 | - | - | - | - | - |
| MR-MBE | JIANG | 62 | 1.193 | 1.035 | 1.375 | 0.176 | 0.073 | 1.52e-02 | - | - | - | - | - |
| MR-PRESSO | JIANG | 61 | 1.162 | 1.108 | 1.219 | 0.150 | 0.024 | 5.49e-08 | - | - | - | - | 1 |

^*^ Myopia GWAS summary statistics from different data repositories (JIANG refers to GCST90044326, ZHOU refers to GCST90435990 from GWAS Catalog; NEALE refers to ukb-a-419, MRCIEU to ukb-b-6353 from IEU Open GWAS; FinnGen R5/R9/R10 refer to summary statistics obtained from different FinnGen releases, respectively) and two newly performed GWAS, utilizing two methods PLINK/SAIGE, described in detail in Supplementary Note S2. All nine GWAS were conducted on a binary trait for myopia.

^†^ “_transformed” refers to the transformed versions of the two myopia GWAS summary statistics conducted on linear scale (ukb-b-6353 from IEU Open GWAS and ukb-a-419 from Neale lab) (effect size of each genetic variant in the two summary statistics files was transformed from an absolute risk difference scale to a log odds ratio scale according to the MRC IEU UK Biobank GWAS pipeline, version 2, 18/01/2019)

# Supplementary Table S4. Full MR results for analyses examining the effect of myopia on POAG without Steiger filtering.

| Method | Sum. stats* | Num. IVs | OR | LCI95 | UCI95 | BETA | SE | P | Qstat | Qpval | Egger intercept | Egger P | Num. MRP outliers |
| --- | --- | --- | --- | --- | --- | --- | --- | --- | --- | --- | --- | --- | --- |
| IVW-MR | JIANG | 32 | 1.142 | 1.040 | 1.253 | 0.132 | 0.048 | 5.56e-03 | 41.162 | 0.105 | - | - | - |
| MR-EGGER | JIANG | 32 | 1.482 | 1.104 | 1.988 | 0.393 | 0.150 | 8.78e-03 | 37.042 | 0.176 | -0.018 | 6.78e-02 | - |
| MR-WMEDIAN | JIANG | 32 | 1.128 | 0.998 | 1.275 | 0.120 | 0.063 | 5.46e-02 | - | - | - | - | - |
| MR-MBE | JIANG | 32 | 1.113 | 0.874 | 1.417 | 0.107 | 0.123 | 3.85e-01 | - | - | - | - | - |
| MR-PRESSO | JIANG | 32 | 1.142 | 1.040 | 1.253 | 0.132 | 0.048 | 9.33e-03 | - | - | - | - | 0 |
| IVW-MR | ZHOU | 1 | 1.005 | 0.899 | 1.125 | 0.005 | 0.057 | 9.26e-01 | - | - | - | - | - |
| MR-EGGER | - | - | - | - | - | - | - | - | - | - | - | - | - |
| MR-WMEDIAN | - | - | - | - | - | - | - | - | - | - | - | - | - |
| MR-MBE | - | - | - | - | - | - | - | - | - | - | - | - | - |
| IVW-MR | NEALE | 17 | 0.066 | 0.018 | 0.242 | -2.718 | 0.663 | 4.14e-05 | 16.096 | 0.446 | - | - | - |
| MR-EGGER | NEALE | 17 | 0.032 | 0.000 | 2.875 | -3.452 | 2.300 | 1.33e-01 | 15.977 | 0.384 | 0.004 | 7.38e-01 | - |
| MR-WMEDIAN | NEALE | 17 | 0.044 | 0.007 | 0.284 | -3.131 | 0.956 | 1.05e-03 | - | - | - | - | - |
| MR-MBE | NEALE | 17 | 0.203 | 0.007 | 6.175 | -1.595 | 1.743 | 3.60e-01 | - | - | - | - | - |
| MR-PRESSO | NEALE | 17 | 0.066 | 0.018 | 0.242 | -2.718 | 0.663 | 8.37e-04 | - | - | - | - | 0 |
| IVW-MR | NEALE_transformed^†^ | 17 | 0.818 | 0.743 | 0.901 | -0.201 | 0.049 | 4.14e-05 | 16.096 | 0.446 | - | - | - |
| MR-EGGER | NEALE_transformed^†^ | 17 | 0.775 | 0.556 | 1.081 | -0.255 | 0.170 | 1.33e-01 | 15.977 | 0.384 | 0.004 | 7.38e-01 | - |
| MR-WMEDIAN | NEALE_transformed^†^ | 17 | 0.794 | 0.691 | 0.911 | -0.231 | 0.071 | 1.05e-03 | - | - | - | - | - |
| MR-MBE | NEALE_transformed^†^ | 17 | 0.889 | 0.691 | 1.144 | -0.118 | 0.129 | 3.60e-01 | - | - | - | - | - |
| MR-PRESSO | NEALE_transformed^†^ | 17 | 0.818 | 0.743 | 0.901 | -0.201 | 0.049 | 8.37e-04 | - | - | - | - | 0 |
| IVW-MR | MRCIEU | 36 | 8.977 | 2.188 | 36.829 | 2.195 | 0.720 | 2.31e-03 | 63.126 | 0.002 | - | - | - |
| MR-EGGER | MRCIEU | 36 | 206.8 | 2.566 | 16672.69 | 5.332 | 2.240 | 1.73e-02 | 59.321 | 0.005 | -0.015 | 1.40e-01 | - |
| MR-WMEDIAN | MRCIEU | 36 | 7.041 | 1.394 | 35.557 | 1.952 | 0.826 | 1.82e-02 | - | - | - | - | - |
| MR-MBE | MRCIEU | 36 | 10.13 | 0.577 | 177.963 | 2.316 | 1.462 | 1.13e-01 | - | - | - | - | - |
| MR-PRESSO | MRCIEU | 35 | 7.124 | 1.932 | 26.271 | 1.963 | 0.666 | 5.73e-03 | - | - | - | - | 1 |
| IVW-MR | MRCIEU_transformed^†^ | 36 | 1.178 | 1.060 | 1.308 | 0.164 | 0.054 | 2.31e-03 | 63.126 | 0.002 | - | - | - |
| MR-EGGER | MRCIEU_transformed^†^ | 36 | 1.488 | 1.073 | 2.064 | 0.397 | 0.167 | 1.73e-02 | 59.321 | 0.005 | -0.015 | 1.40e-01 | - |
| MR-WMEDIAN | MRCIEU_transformed^†^ | 36 | 1.157 | 1.025 | 1.305 | 0.145 | 0.062 | 1.82e-02 | - | - | - | - | - |
| MR-MBE | MRCIEU_transformed^†^ | 36 | 1.188 | 0.960 | 1.472 | 0.173 | 0.109 | 1.13e-01 | - | - | - | - | - |
| MR-PRESSO | MRCIEU_transformed^†^ | 35 | 1.158 | 1.050 | 1.276 | 0.146 | 0.050 | 5.73e-03 | - | - | - | - | 1 |
| IVW-MR | FinnGenR9 | 5 | 1.108 | 0.992 | 1.238 | 0.102 | 0.057 | 7.04e-02 | 8.3 | 0.081 | - | - | - |
| MR-EGGER | FinnGenR9 | 5 | 0.833 | 0.671 | 1.035 | -0.183 | 0.111 | 9.88e-02 | 0.705 | 0.872 | 0.062 | 5.85e-03 | - |
| MR-WMEDIAN | FinnGenR9 | 5 | 1.149 | 1.031 | 1.281 | 0.139 | 0.055 | 1.20e-02 | - | - | - | - | - |
| MR-MBE | FinnGenR9 | 5 | 1.142 | 0.972 | 1.341 | 0.133 | 0.082 | 1.06e-01 | - | - | - | - | - |
| MR-PRESSO | FinnGenR9 | 5 | 1.108 | 0.992 | 1.238 | 0.102 | 0.057 | 1.45e-01 | - | - | - | - | 0 |
| IVW-MR | FinnGenR10 | 8 | 1.059 | 0.966 | 1.162 | 0.058 | 0.047 | 2.22e-01 | 11.148 | 0.132 | - | - | - |
| MR-EGGER | FinnGenR10 | 8 | 0.816 | 0.679 | 0.982 | -0.203 | 0.094 | 3.14e-02 | 2.093 | 0.911 | 0.059 | 2.62e-03 | - |
| MR-WMEDIAN | FinnGenR10 | 8 | 1.033 | 0.933 | 1.144 | 0.033 | 0.052 | 5.29e-01 | - | - | - | - | - |
| MR-MBE | FinnGenR10 | 8 | 1.022 | 0.892 | 1.171 | 0.022 | 0.069 | 7.53e-01 | - | - | - | - | - |
| MR-PRESSO | FinnGenR10 | 8 | 1.059 | 0.966 | 1.162 | 0.058 | 0.047 | 2.62e-01 | - | - | - | - | 0 |
| IVW-MR | FinnGenR11 | 7 | 1.064 | 0.955 | 1.185 | 0.062 | 0.055 | 2.59e-01 | 11.81 | 0.066 | - | - | - |
| MR-EGGER | FinnGenR11 | 7 | 0.885 | 0.691 | 1.132 | -0.123 | 0.126 | 3.30e-01 | 7.845 | 0.165 | 0.034 | 1.12e-01 | - |
| MR-WMEDIAN | FinnGenR11 | 7 | 1.030 | 0.928 | 1.144 | 0.030 | 0.053 | 5.74e-01 | - | - | - | - | - |
| MR-MBE | FinnGenR11 | 7 | 1.006 | 0.886 | 1.142 | 0.006 | 0.065 | 9.30e-01 | - | - | - | - | - |
| MR-PRESSO | FinnGenR11 | 7 | 1.064 | 0.955 | 1.185 | 0.062 | 0.055 | 3.02e-01 | - | - | - | - | 0 |
| IVW-MR | SAIGE | 48 | 1.119 | 1.033 | 1.213 | 0.113 | 0.041 | 5.72e-03 | 147.671 | 0 | - | - | - |
| MR-EGGER | SAIGE | 48 | 1.221 | 0.996 | 1.496 | 0.199 | 0.104 | 5.48e-02 | 145.071 | 0 | -0.009 | 3.64e-01 | - |
| MR-WMEDIAN | SAIGE | 48 | 1.119 | 1.039 | 1.205 | 0.113 | 0.038 | 2.88e-03 | - | - | - | - | - |
| MR-MBE | SAIGE | 48 | 1.105 | 0.985 | 1.239 | 0.099 | 0.059 | 9.01e-02 | - | - | - | - | - |
| MR-PRESSO | SAIGE | 45 | 1.126 | 1.064 | 1.192 | 0.119 | 0.029 | 1.82e-04 | - | - | - | - | 3 |
| IVW-MR | PLINK | 54 | 1.105 | 1.028 | 1.188 | 0.100 | 0.037 | 6.47e-03 | 132.248 | 0 | - | - | - |
| MR-EGGER | PLINK | 54 | 1.208 | 0.980 | 1.490 | 0.189 | 0.107 | 7.68e-02 | 130.268 | 0 | -0.009 | 3.74e-01 | - |
| MR-WMEDIAN | PLINK | 54 | 1.106 | 1.027 | 1.192 | 0.101 | 0.038 | 8.07e-03 | - | - | - | - | - |
| MR-MBE | PLINK | 54 | 1.109 | 0.975 | 1.262 | 0.103 | 0.066 | 1.16e-01 | - | - | - | - | - |
| MR-PRESSO | PLINK | 50 | 1.113 | 1.054 | 1.175 | 0.107 | 0.028 | 3.40e-04 | - | - | - | - | 4 |

^*^ Myopia GWAS summary statistics from different data repositories (JIANG refers to GCST90044326, ZHOU refers to GCST90435990 from GWAS Catalog; NEALE refers to ukb-a-419, MRC IEU to ukb-b-6353 from IEU Open GWAS; FinnGen R5/R9/R10 refer to summary statistics obtained from different FinnGen releases, respectively) and two newly performed GWAS, utilizing two methods PLINK/SAIGE, described in detail in Supplementary Note S2. All nine GWAS were conducted on a binary trait for myopia.

^†^ “_transformed” refers to the transformed versions of the two myopia GWAS summary statistics conducted on linear scale (ukb-b-6353 from IEU Open GWAS and ukb-a-419 from Neale lab) (effect size of each genetic variant in the two summary statistics files was transformed from an absolute risk difference scale to a log odds ratio scale according to the MRC IEU UK Biobank GWAS pipeline, version 2, 18/01/2019)

# Supplementary Table S5. Full MR results for analyses examining the effect of myopia on POAG with Steiger filtering.

| Method | Sum. stats* | Num. IVs | OR | LCI95 | UCI95 | BETA | SE | P | Qstat | Qpval | Egger intercept | Egger P | Num. MRP outliers |
| --- | --- | --- | --- | --- | --- | --- | --- | --- | --- | --- | --- | --- | --- |
| IVW-MR | JIANG | 32 | 1.142 | 1.040 | 1.253 | 0.132 | 0.048 | 5.56e-03 | 41.162 | 0.105 | - | - | - |
| MR-EGGER | JIANG | 32 | 1.482 | 1.104 | 1.988 | 0.393 | 0.150 | 8.78e-03 | 37.042 | 0.176 | -0.018 | 6.78e-02 | - |
| MR-WMEDIAN | JIANG | 32 | 1.128 | 0.998 | 1.275 | 0.120 | 0.063 | 5.46e-02 | - | - | - | - | - |
| MR-MBE | JIANG | 32 | 1.113 | 0.874 | 1.418 | 0.107 | 0.124 | 3.86e-01 | - | - | - | - | - |
| MR-PRESSO | JIANG | 32 | 1.142 | 1.040 | 1.253 | 0.132 | 0.048 | 9.33e-03 | - | - | - | - | 0 |
| IVW-MR | ZHOU | 1 | 1.005 | 0.899 | 1.125 | 0.005 | 0.057 | 9.26e-01 | - | - | - | - | - |
| MR-EGGER | - | - | - | - | - | - | - | - | - | - | - | - | - |
| MR-WMEDIAN | - | - | - | - | - | - | - | - | - | - | - | - | - |
| MR-MBE | - | - | - | - | - | - | - | - | - | - | - | - | - |
| IVW-MR | NEALE | 17 | 0.066 | 0.018 | 0.242 | -2.718 | 0.663 | 4.14e-05 | 16.096 | 0.446 | - | - | - |
| MR-EGGER | NEALE | 17 | 0.032 | 0.000 | 2.875 | -3.452 | 2.300 | 1.33e-01 | 15.977 | 0.384 | 0.004 | 7.38e-01 | - |
| MR-WMEDIAN | NEALE | 17 | 0.044 | 0.007 | 0.284 | -3.131 | 0.956 | 1.05e-03 | - | - | - | - | - |
| MR-MBE | NEALE | 17 | 0.203 | 0.007 | 6.191 | -1.595 | 1.744 | 3.60e-01 | - | - | - | - | - |
| MR-PRESSO | NEALE | 17 | 0.066 | 0.018 | 0.242 | -2.718 | 0.663 | 8.37e-04 | - | - | - | - | 0 |
| IVW-MR | NEALE_transformed^†^ | 17 | 0.818 | 0.743 | 0.901 | -0.201 | 0.049 | 4.14e-05 | 16.096 | 0.446 | - | - | - |
| MR-EGGER | NEALE_transformed^†^ | 17 | 0.775 | 0.556 | 1.081 | -0.255 | 0.170 | 1.33e-01 | 15.977 | 0.384 | 0.004 | 7.38e-01 | - |
| MR-WMEDIAN | NEALE_transformed^†^ | 17 | 0.794 | 0.691 | 0.911 | -0.231 | 0.071 | 1.05e-03 | - | - | - | - | - |
| MR-MBE | NEALE_transformed^†^ | 17 | 0.889 | 0.691 | 1.144 | -0.118 | 0.129 | 3.60e-01 | - | - | - | - | - |
| MR-PRESSO | NEALE_transformed^†^ | 17 | 0.818 | 0.743 | 0.901 | -0.201 | 0.049 | 8.37e-04 | - | - | - | - | 0 |
| IVW-MR | MRCIEU | 33 | 5.199 | 1.485 | 18.202 | 1.648 | 0.639 | 9.93e-03 | 43.038 | 0.092 | - | - | - |
| MR-EGGER | MRCIEU | 33 | 335.112 | 8.272 | 13575.811 | 5.814 | 1.889 | 2.08e-03 | 36.643 | 0.233 | -0.021 | 2.00e-02 | - |
| MR-WMEDIAN | MRCIEU | 33 | 6.086 | 1.188 | 31.168 | 1.806 | 0.833 | 3.02e-02 | - | - | - | - | - |
| MR-MBE | MRCIEU | 33 | 7.964 | 0.386 | 164.391 | 2.075 | 1.545 | 1.79e-01 | - | - | - | - | - |
| MR-PRESSO | MRCIEU | 33 | 5.199 | 1.485 | 18.202 | 1.648 | 0.639 | 1.47e-02 | - | - | - | - | 0 |
| IVW-MR | MRCIEU_transformed^†^ | 35 | 1.158 | 1.050 | 1.276 | 0.146 | 0.050 | 3.19e-03 | 51.577 | 0.027 | - | - | - |
| MR-EGGER | MRCIEU_transformed^†^ | 35 | 1.419 | 1.048 | 1.921 | 0.350 | 0.155 | 2.36e-02 | 48.744 | 0.038 | -0.013 | 1.65e-01 | - |
| MR-WMEDIAN | MRCIEU_transformed^†^ | 35 | 1.147 | 1.016 | 1.295 | 0.138 | 0.062 | 2.61e-02 | - | - | - | - | - |
| MR-MBE | MRCIEU_transformed^†^ | 35 | 1.175 | 0.940 | 1.469 | 0.161 | 0.114 | 1.56e-01 | - | - | - | - | - |
| MR-PRESSO | MRCIEU_transformed^†^ | 35 | 1.158 | 1.050 | 1.276 | 0.146 | 0.050 | 5.73e-03 | - | - | - | - | 0 |
| IVW-MR | FinnGenR9 | 5 | 1.108 | 0.992 | 1.238 | 0.102 | 0.057 | 7.04e-02 | 8.300 | 0.081 | - | - | - |
| MR-EGGER | FinnGenR9 | 5 | 0.833 | 0.671 | 1.035 | -0.183 | 0.111 | 9.88e-02 | 0.705 | 0.872 | 0.062 | 5.85e-03 | - |
| MR-WMEDIAN | FinnGenR9 | 5 | 1.149 | 1.031 | 1.281 | 0.139 | 0.055 | 1.20e-02 | - | - | - | - | - |
| MR-MBE | FinnGenR9 | 5 | 1.142 | 0.972 | 1.341 | 0.133 | 0.082 | 1.06e-01 | - | - | - | - | - |
| MR-PRESSO | FinnGenR9 | 5 | 1.108 | 0.992 | 1.238 | 0.102 | 0.057 | 1.45e-01 | - | - | - | - | 0 |
| IVW-MR | FinnGenR10 | 8 | 1.059 | 0.966 | 1.162 | 0.058 | 0.047 | 2.22e-01 | 11.148 | 0.132 | - | - | - |
| MR-EGGER | FinnGenR10 | 8 | 0.816 | 0.679 | 0.982 | -0.203 | 0.094 | 3.14e-02 | 2.093 | 0.911 | 0.059 | 2.62e-03 | - |
| MR-WMEDIAN | FinnGenR10 | 8 | 1.033 | 0.933 | 1.144 | 0.033 | 0.052 | 5.29e-01 | - | - | - | - | - |
| MR-MBE | FinnGenR10 | 8 | 1.022 | 0.892 | 1.171 | 0.022 | 0.069 | 7.53e-01 | - | - | - | - | - |
| MR-PRESSO | FinnGenR10 | 8 | 1.059 | 0.966 | 1.162 | 0.058 | 0.047 | 2.62e-01 | - | - | - | - | 0 |
| IVW-MR | FinnGenR11 | 7 | 1.064 | 0.955 | 1.185 | 0.062 | 0.055 | 2.59e-01 | 11.81 | 0.066 | - | - | - |
| MR-EGGER | FinnGenR11 | 7 | 0.885 | 0.691 | 1.132 | -0.123 | 0.126 | 3.30e-01 | 7.845 | 0.165 | 0.034 | 1.12e-01 | - |
| MR-WMEDIAN | FinnGenR11 | 7 | 1.030 | 0.928 | 1.144 | 0.030 | 0.053 | 5.74e-01 | - | - | - | - | - |
| MR-MBE | FinnGenR11 | 7 | 1.006 | 0.886 | 1.142 | 0.006 | 0.065 | 9.30e-01 | - | - | - | - | - |
| MR-PRESSO | FinnGenR11 | 7 | 1.064 | 0.955 | 1.185 | 0.062 | 0.055 | 3.02e-01 | - | - | - | - | 0 |
| IVW-MR | SAIGE | 43 | 1.101 | 1.024 | 1.185 | 0.096 | 0.037 | 9.70e-03 | 103.070 | 0 | - | - | - |
| MR-EGGER | SAIGE | 43 | 1.265 | 1.059 | 1.511 | 0.235 | 0.091 | 9.65e-02 | 96.524 | 0 | -0.016 | 9.54e-02 | - |
| MR-WMEDIAN | SAIGE | 43 | 1.112 | 1.031 | 1.201 | 0.106 | 0.039 | 6.30e-03 | - | - | - | - | - |
| MR-MBE | SAIGE | 43 | 1.103 | 0.979 | 1.242 | 0.098 | 0.061 | 1.06e-01 | - | - | - | - | - |
| MR-PRESSO | SAIGE | 41 | 1.131 | 1.065 | 1.201 | 0.123 | 0.031 | 2.62e-04 | - | - | - | - | 2 |
| IVW-MR | PLINK | 47 | 1.095 | 1.018 | 1.178 | 0.091 | 0.037 | 1.49e-02 | 108.353 | 0 | - | - | - |
| MR-EGGER | PLINK | 47 | 1.214 | 0.990 | 1.490 | 0.194 | 0.104 | 6.29e-02 | 105.710 | 0 | -0.011 | 2.89e-01 | - |
| MR-WMEDIAN | PLINK | 47 | 1.102 | 1.020 | 1.191 | 0.097 | 0.040 | 1.39e-02 | - | - | - | - | - |
| MR-MBE | PLINK | 47 | 1.095 | 0.960 | 1.249 | 0.091 | 0.067 | 1.75e-01 | - | - | - | - | - |
| MR-PRESSO | PLINK | 45 | 1.127 | 1.060 | 1.198 | 0.119 | 0.031 | 3.90e-04 | - | - | - | - | 2 |

^*^ Myopia GWAS summary statistics from different data repositories (JIANG refers to GCST90044326, ZHOU refers to GCST90435990 from GWAS Catalog; NEALE refers to ukb-a-419, MRC IEU to ukb-b-6353 from IEU Open GWAS; FinnGen R5/R9/R10 refer to summary statistics obtained from different FinnGen releases, respectively) and two newly performed GWAS, utilizing two methods PLINK/SAIGE, described in detail in Supplementary Note S2. All nine GWAS were conducted on a binary trait for myopia.

^†^ “_transformed” refers to the transformed versions of the two myopia GWAS summary statistics conducted on linear scale (ukb-b-6353 from IEU Open GWAS and ukb-a-419 from Neale lab) (effect size of each genetic variant in the two summary statistics files was transformed from an absolute risk difference scale to a log odds ratio scale according to the MRC IEU UK Biobank GWAS pipeline, version 2, 18/01/2019)

# Supplementary Table S6. Full MR results for re-analyses examining the effect of myopia on any diabetic retinopathy (AnyDR) and proliferative diabetic retinopathy (PDR).

| Method | Sumstats^*^ | Num. IVs | OR | LCI95 | UCI95 | BETA | SE | P | Qstat | Qpval | Egger_intercept | Egger_P | MRP_outliers |
| --- | --- | --- | --- | --- | --- | --- | --- | --- | --- | --- | --- | --- | --- |
| IVW-MR | PLINK - AnyDR | 41 | 1.131 | 1.074 | 1.191 | 0.123 | 0.026 | 2.80e-06 | 45.692 | 0.248 | - | - | - |
| MR-EGGER | PLINK - AnyDR | 41 | 1.155 | 1.005 | 1.328 | 0.144 | 0.071 | 4.25e-02 | 45.574 | 0.217 | -0.002 | 7.50e-01 | - |
| MR-WMEDIAN | PLINK - AnyDR | 41 | 1.139 | 1.059 | 1.225 | 0.130 | 0.037 | 4.63e-04 | - | - | - | - | - |
| MR-MBE | PLINK - AnyDR | 41 | 1.210 | 1.065 | 1.374 | 0.190 | 0.065 | 3.41e-03 | - | - | - | - | - |
| MR-PRESSO | PLINK - AnyDR | 41 | 1.131 | 1.074 | 1.191 | 0.123 | 0.026 | 3.21e-05 | - | - | - | - | 0 |
| IVW-MR | SAIGE - AnyDR | 36 | 1.138 | 1.082 | 1.197 | 0.129 | 0.026 | 4.27e-07 | 36.382 | 0.404 | - | - | - |
| MR-EGGER | SAIGE - AnyDR | 36 | 1.180 | 1.040 | 1.338 | 0.165 | 0.064 | 1.03e-02 | 35.990 | 0.376 | -0.004 | 5.43e-01 | - |
| MR-WMEDIAN | SAIGE - AnyDR | 36 | 1.200 | 1.115 | 1.291 | 0.182 | 0.037 | 1.09e-06 | - | - | - | - | - |
| MR-MBE | SAIGE - AnyDR | 36 | 1.193 | 1.072 | 1.327 | 0.176 | 0.054 | 1.21e-03 | - | - | - | - | - |
| MR-PRESSO | SAIGE - AnyDR | 36 | 1.138 | 1.082 | 1.197 | 0.129 | 0.026 | 1.35e-05 | - | - | - | - | 0 |
| IVW-MR | PLINK - PDR | 41 | 1.159 | 1.088 | 1.235 | 0.147 | 0.032 | 5.44e-06 | 42.690 | 0.356 | - | - | - |
| MR-EGGER | PLINK - PDR | 41 | 1.232 | 1.039 | 1.462 | 0.209 | 0.087 | 1.67e-02 | 42.071 | 0.339 | -0.006 | 4.49e-01 | - |
| MR-WMEDIAN | PLINK - PDR | 41 | 1.135 | 1.035 | 1.244 | 0.126 | 0.047 | 7.10e-03 | - | - | - | - | - |
| MR-MBE | PLINK - PDR | 41 | 1.091 | 0.916 | 1.299 | 0.087 | 0.089 | 3.29e-01 | - | - | - | - | - |
| MR-PRESSO | PLINK - PDR | 41 | 1.159 | 1.088 | 1.235 | 0.147 | 0.032 | 4.95e-05 | - | - | - | - | 0 |
| IVW-MR | SAIGE - PDR | 36 | 1.184 | 1.110 | 1.264 | 0.169 | 0.033 | 3.38e-07 | 37.614 | 0.350 | - | - | - |
| MR-EGGER | SAIGE - PDR | 36 | 1.217 | 1.032 | 1.434 | 0.196 | 0.084 | 1.93e-02 | 37.477 | 0.313 | -0.003 | 7.25e-01 | - |
| MR-WMEDIAN | SAIGE - PDR | 36 | 1.200 | 1.093 | 1.318 | 0.182 | 0.048 | 1.39e-04 | - | - | - | - | - |
| MR-MBE | SAIGE - PDR | 36 | 1.237 | 1.069 | 1.431 | 0.212 | 0.075 | 4.36e-03 | - | - | - | - | - |
| MR-PRESSO | SAIGE - PDR | 36 | 1.184 | 1.110 | 1.264 | 0.169 | 0.033 | 1.18e-05 | - | - | - | - | 0 |

^*^MR analyses conducted using the myopia binary trait (PLINK/SAIGE) as exposure and AnyDR/PDR as outcomes. Summary statistics of AnyDR and PDR were obtained from FinnGen R5 to match the source used in the original MR study.

# Supplementary Table S7. Full MR results for re-analyses examining the effect of myopia on age-related cataract (ARC).

| Method | Sumstats^*^ | Num. IVs | OR | LCI95 | UCI95 | BETA | SE | P | Qstat | Qpval | Egger_intercept | Egger_P | MRP_outliers |
| --- | --- | --- | --- | --- | --- | --- | --- | --- | --- | --- | --- | --- | --- |
| IVW-MR | PLINK-ARC | 40 | 1.160 | 1.101 | 1.222 | 0.148 | 0.027 | 2.81e-08 | 58.656 | 0.022 | - | - | - |
| MR-EGGER | PLINK-ARC | 40 | 1.169 | 1.016 | 1.346 | 0.156 | 0.072 | 2.95e-02 | 58.632 | 0.017 | -0.001 | 9.00e-01 | - |
| MR-WMEDIAN | PLINK-ARC | 40 | 1.131 | 1.058 | 1.209 | 0.123 | 0.034 | 2.94e-04 | - | - | - | - | - |
| MR-MBE | PLINK-ARC | 40 | 1.074 | 0.940 | 1.226 | 0.071 | 0.068 | 2.93e-01 | - | - | - | - | - |
| MR-PRESSO | PLINK-ARC | 40 | 1.160 | 1.101 | 1.222 | 0.148 | 0.027 | 2.16e-06 | - | - | - | - | 0 |
| IVW-MR | SAIGE-ARC | 35 | 1.166 | 1.106 | 1.230 | 0.154 | 0.027 | 1.67e-08 | 51.610 | 0.027 | - | - | - |
| MR-EGGER | SAIGE-ARC | 35 | 1.213 | 1.061 | 1.388 | 0.194 | 0.069 | 4.86e-03 | 50.994 | 0.024 | -0.005 | 5.28e-01 | - |
| MR-WMEDIAN | SAIGE-ARC | 35 | 1.158 | 1.081 | 1.241 | 0.147 | 0.035 | 2.98e-05 | - | - | - | - | - |
| MR-MBE | SAIGE-ARC | 35 | 1.091 | 0.969 | 1.228 | 0.087 | 0.060 | 1.50e-01 | - | - | - | - | - |
| MR-PRESSO | SAIGE-ARC | 35 | 1.166 | 1.106 | 1.230 | 0.154 | 0.027 | 2.51e-06 | - | - | - | - | 0 |
| IVW-MR | MRCIEU_transformed^†^ - ARC | 28 | 1.203 | 1.100 | 1.316 | 0.185 | 0.046 | 5.27e-05 | 37.00 | 0.095 | - | - | - |
| MR-EGGER | MRCIEU_transformed^†^ - ARC | 28 | 1.399 | 1.064 | 1.841 | 0.336 | 0.140 | 1.63e-02 | 35.236 | 0.107 | -0.010 | 2.54e-01 | - |
| MR-WMEDIAN | MRCIEU_transformed^†^ - ARC | 28 | 1.175 | 1.043 | 1.323 | 0.161 | 0.061 | 7.83e-03 | - | - | - | - | - |
| MR-MBE | MRCIEU_transformed^†^ - ARC | 28 | 1.077 | 0.821 | 1.413 | 0.074 | 0.138 | 5.92e-01 | - | - | - | - | - |
| MR-PRESSO | MRCIEU_transformed^†^ - ARC | 28 | 1.203 | 1.100 | 1.316 | 0.185 | 0.046 | 3.95e-04 | - | - | - | - | 0 |

^*^MR analyses conducted using the myopia binary trait (PLINK/SAIGE) as exposure and ARC as outcome. Summary statistics of ARC was obtained from FinnGen R5 to match the source used in the original MR study.

^†^ MRC IEU_transformed refers to the transformed version of ukb-b-6353 myopia GWAS summary statistics from IEU Open GWAS (effect size of each genetic variant in the ukb-b-6353 summary statistics file was transformed from an absolute risk difference scale to a log odds ratio scale according to the MRC IEU UK Biobank GWAS pipeline, version 2, 18/01/2019)

# Supplementary Table S8. Full MR results for re-analyses examining the effect of myopia on disorders of the vitreous body (vitrbodyglobe) and other unspecified disorders of the vitreous body (vitroth).

| Method | Sumstats | Num_IVs | OR | LCI95 | UCI95 | BETA | SE | P | Qstat | Qpval | Egger_intercept | Egger_P | MRP_outliers |
| --- | --- | --- | --- | --- | --- | --- | --- | --- | --- | --- | --- | --- | --- |
| IVW-MR | Plink-vitrbodyglobe | 41 | 1.245 | 1.160 | 1.337 | 0.219 | 0.036 | 1.54e-09 | 47.288 | 0.199 | - | - | - |
| MR-EGGER | Plink-vitrbodyglobe | 41 | 1.144 | 0.946 | 1.382 | 0.134 | 0.097 | 1.65e-01 | 46.215 | 0.199 | 0.009 | 3.41e-01 | - |
| MR-WMEDIAN | Plink-vitrbodyglobe | 41 | 1.259 | 1.142 | 1.387 | 0.230 | 0.049 | 3.32e-06 | - | - | - | - | - |
| MR-MBE | Plink-vitrbodyglobe | 41 | 1.232 | 1.065 | 1.426 | 0.209 | 0.075 | 5.08e-03 | - | - | - | - | - |
| MR-PRESSO | Plink-vitrbodyglobe | 41 | 1.245 | 1.160 | 1.337 | 0.219 | 0.036 | 4.15e-07 | - | - | - | - | 0 |
| IVW-MR | Saige-vitrbodyglobe | 35 | 1.232 | 1.152 | 1.317 | 0.208 | 0.034 | 1.23e-09 | 32.615 | 0.535 | - | - | - |
| MR-EGGER | Saige-vitrbodyglobe | 35 | 1.169 | 0.989 | 1.383 | 0.156 | 0.086 | 6.78e-02 | 32.172 | 0.508 | 0.006 | 5.05e-01 | - |
| MR-WMEDIAN | Saige-vitrbodyglobe | 35 | 1.230 | 1.117 | 1.354 | 0.207 | 0.049 | 2.62e-05 | - | - | - | - | - |
| MR-MBE | Saige-vitrbodyglobe | 35 | 1.209 | 1.067 | 1.370 | 0.190 | 0.064 | 2.82e-03 | - | - | - | - | - |
| MR-PRESSO | Saige-vitrbodyglobe | 35 | 1.232 | 1.153 | 1.316 | 0.208 | 0.034 | 4.68e-07 | - | - | - | - | 0 |
| IVW-MR | MRCIEU_transformed^†^-vitrbodyglobe | 31 | 1.227 | 1.093 | 1.376 | 0.204 | 0.059 | 5.01e-04 | 26.521 | 0.648 | - | - | - |
| MR-EGGER | MRCIEU_transformed^†^ -vitrbodyglobe | 31 | 1.822 | 1.281 | 2.591 | 0.600 | 0.180 | 8.37e-04 | 21.086 | 0.856 | -0.026 | 1.97e-02 | - |
| MR-WMEDIAN | MRCIEU_transformed^†^ -vitrbodyglobe | 31 | 1.284 | 1.088 | 1.516 | 0.250 | 0.085 | 3.18e-03 | - | - | - | - | - |
| MR-MBE | MRCIEU_transformed^†^ -vitrbodyglobe | 31 | 1.343 | 1.047 | 1.724 | 0.295 | 0.127 | 2.04e-02 | - | - | - | - | - |
| MR-PRESSO | MRCIEU_transformed^†^ -vitrbodyglobe | 31 | 1.227 | 1.101 | 1.367 | 0.204 | 0.055 | 8.62e-04 | - | - | - | - | 0 |
| IVW-MR | Plink-vitroth | 40 | 1.277 | 1.186 | 1.376 | 0.245 | 0.038 | 1.24e-10 | 28.025 | 0.904 | - | - | - |
| MR-EGGER | Plink-vitroth | 40 | 1.266 | 1.039 | 1.542 | 0.236 | 0.101 | 1.95e-02 | 28.015 | 0.882 | 0.001 | 9.22e-01 | - |
| MR-WMEDIAN | Plink-vitroth | 40 | 1.298 | 1.164 | 1.449 | 0.261 | 0.056 | 3.06e-06 | - | - | - | - | - |
| MR-MBE | Plink-vitroth | 40 | 1.320 | 1.128 | 1.546 | 0.278 | 0.080 | 5.47e-04 | - | - | - | - | - |
| MR-PRESSO | Plink-vitroth | 40 | 1.277 | 1.199 | 1.361 | 0.245 | 0.032 | 3.36e-09 | - | - | - | - | 0 |
| IVW-MR | Saige-vitroth | 35 | 1.280 | 1.187 | 1.381 | 0.247 | 0.039 | 1.76e-10 | 24.518 | 0.884 | - | - | - |
| MR-EGGER | Saige-vitroth | 35 | 1.247 | 1.032 | 1.507 | 0.221 | 0.097 | 2.22e-02 | 24.430 | 0.860 | 0.003 | 7.67e-01 | - |
| MR-WMEDIAN | Saige-vitroth | 35 | 1.259 | 1.127 | 1.405 | 0.230 | 0.056 | 4.15e-05 | - | - | - | - | - |
| MR-MBE | Saige-vitroth | 35 | 1.225 | 1.060 | 1.417 | 0.203 | 0.074 | 6.14e-03 | - | - | - | - | - |
| MR-PRESSO | Saige-vitroth | 35 | 1.280 | 1.200 | 1.365 | 0.247 | 0.033 | 1.01e-08 | - | - | - | - | 0 |
| IVW-MR | MRCIEU_transformed^†^ -vitroth | 31 | 1.327 | 1.165 | 1.511 | 0.283 | 0.066 | 1.93e-05 | 27.751 | 0.584 | - | - | - |
| MR-EGGER | MRCIEU_transformed^†^ -vitroth | 31 | 2.123 | 1.427 | 3.159 | 0.753 | 0.203 | 2.03e-04 | 21.729 | 0.831 | -0.031 | 1.41e-02 | - |
| MR-WMEDIAN | MRCIEU_transformed^†^ -vitroth | 31 | 1.439 | 1.193 | 1.736 | 0.364 | 0.096 | 1.46e-04 | - | - | - | - | - |
| MR-MBE | MRCIEU_transformed^†^ -vitroth | 31 | 1.594 | 1.206 | 2.107 | 0.466 | 0.142 | 1.05e-03 | - | - | - | - | - |
| MR-PRESSO | MRCIEU_transformed^†^ -vitroth | 31 | 1.327 | 1.171 | 1.503 | 0.283 | 0.064 | 1.12e-04 | - | - | - | - | 0 |

^*^MR analyses conducted using the myopia binary trait (plink/saige) as exposure and vitrbodyglobe/vitroth as outcomes. Summary statistics of vitrbodyglobe/vitroth were obtained from FinnGen R5 to match the source used in the original MR study.

^†^ MRC IEU_transformed refers to the transformed version of ukb-b-6353 myopia GWAS summary statistics from IEU Open GWAS (effect size of each genetic variant in the ukb-b-6353 summary statistics file was transformed from an absolute risk difference scale to a log odds ratio scale according to the MRC IEU UK Biobank GWAS pipeline, version 2, 18/01/2019)

# Supplementary Table S9. Full MR results for re-analyses examining the effect of asthma and immunoglobulin E (IgE) on myopia.

| Method | Sumstats* | Num_IVs | OR | LCI95 | UCI95 | BETA | SE | P | Qstat | Qpval | Egger_intercept | Egger_P | MRP_outliers |
| --- | --- | --- | --- | --- | --- | --- | --- | --- | --- | --- | --- | --- | --- |
| IVW-MR | a_new - SAIGE | 3 | 0.900 | 0.795 | 1.020 | -0.105 | 0.063 | 9.83e-02 | 3.170 | 0.205 | - | - | - |
| MR-EGGER | a_new - SAIGE | 3 | 1.307 | 0.449 | 3.801 | 0.268 | 0.545 | 6.23e-01 | 2.139 | 0.144 | -0.044 | 4.88e-01 | - |
| MR-WMEDIAN | a_new - SAIGE | 3 | 0.904 | 0.780 | 1.047 | -0.101 | 0.075 | 1.78e-01 | - | - | - | - | - |
| MR-MBE | a_new - SAIGE | 3 | 0.958 | 0.821 | 1.118 | -0.043 | 0.079 | 5.86e-01 | - | - | - | - | - |
| IVW-MR | a_new - PLINK | 3 | 0.871 | 0.766 | 0.990 | -0.138 | 0.065 | 3.50e-02 | 449 | 0.799 | - | - | - |
| MR-EGGER | a_new - PLINK | 3 | 1.066 | 0.502 | 2.263 | 0.064 | 0.384 | 8.68e-01 | 165 | 0.684 | -0.024 | 5.94e-01 | - |
| MR-WMEDIAN | a_new - PLINK | 3 | 0.863 | 0.747 | 0.997 | -0.147 | 0.074 | 4.54e-02 | - | - | - | - | - |
| MR-MBE | a_new - PLINK | 3 | 0.909 | 0.772 | 1.070 | -0.096 | 0.083 | 2.50e-01 | - | - | - | - | - |
| IVW-MR | a_original - SAIGE | 4 | 0.907 | 0.807 | 1.019 | -0.098 | 0.060 | 1.01e-01 | 3.242 | 0.356 | - | - | - |
| MR-EGGER | a_original - SAIGE | 4 | 1.265 | 0.576 | 2.780 | 0.235 | 0.402 | 5.59e-01 | 2.399 | 0.301 | -0.039 | 4.02e-01 | - |
| MR-WMEDIAN | a_original - SAIGE | 4 | 0.940 | 0.811 | 1.089 | -0.062 | 0.075 | 4.10e-01 | - | - | - | - | - |
| MR-MBE | a_original - SAIGE | 4 | 0.963 | 0.818 | 1.134 | -0.038 | 0.083 | 6.49e-01 | - | - | - | - | - |
| MR-PRESSO | a_original - SAIGE | 4 | 0.907 | 0.807 | 1.019 | -0.098 | 0.060 | 2.00e-01 | - | - | - | - | 0 |
| IVW-MR | a_original - PLINK | 5 | 0.936 | 0.863 | 1.016 | -0.066 | 0.042 | 1.14e-01 | 3.188 | 0.527 | - | - | - |
| MR-EGGER | a_original - PLINK | 5 | 1.088 | 0.900 | 1.315 | 0.085 | 0.097 | 3.82e-01 | 229 | 0.973 | -0.025 | 8.54e-02 | - |
| MR-WMEDIAN | a_original - PLINK | 5 | 0.936 | 0.845 | 1.037 | -0.066 | 0.052 | 2.09e-01 | - | - | - | - | - |
| MR-MBE | a_original - PLINK | 5 | 0.993 | 0.893 | 1.103 | -0.007 | 0.054 | 8.92e-01 | - | - | - | - | - |
| MR-PRESSO | a_original - PLINK | 5 | 0.936 | 0.871 | 1.007 | -0.066 | 0.037 | 1.52e-01 | - | - | - | - | 0 |
| Wald Ratio | I_new - SAIGE | 1 | 1.042 | 0.902 | 1.204 | 0.042 | 0.074 | 5.73e-01 | - | - | - | - | - |
| Wald Ratio | I_new - PLINK | 1 | 1.263 | 1.071 | 1.489 | 0.233 | 0.084 | 5.45e-03 | - | - | - | - | - |
| IVW-MR | I_original - SAIGE | 19 | 0.995 | 0.954 | 1.037 | -0.005 | 0.021 | 8.00e-01 | 13.765 | 0.744 | - | - | - |
| MR-EGGER | I_original - SAIGE | 19 | 0.950 | 0.855 | 1.055 | -0.051 | 0.054 | 3.37e-01 | 12.888 | 0.744 | 0.009 | 3.49e-01 | - |
| MR-WMEDIAN | I_original - SAIGE | 19 | 0.989 | 0.933 | 1.047 | -0.011 | 0.029 | 6.98e-01 | - | - | - | - | - |
| MR-MBE | I_original - SAIGE | 19 | 0.977 | 0.882 | 1.081 | -0.024 | 0.052 | 6.48e-01 | - | - | - | - | - |
| MR-PRESSO | I_original - SAIGE | 19 | 0.995 | 0.959 | 1.032 | -0.005 | 0.019 | 7.75e-01 | - | - | - | - | 0 |
| IVW-MR | I_original - PLINK | 19 | 1.019 | 0.971 | 1.068 | 0.018 | 0.024 | 4.49e-01 | 22.190 | 0.224 | - | - | - |
| MR-EGGER | I_original - PLINK | 19 | 0.958 | 0.850 | 1.080 | -0.043 | 0.061 | 4.85e-01 | 20.741 | 0.238 | 0.013 | 2.76e-01 | - |
| MR-WMEDIAN | I_original - PLINK | 19 | 1.039 | 0.976 | 1.106 | 0.038 | 0.032 | 2.28e-01 | - | - | - | - | - |
| MR-MBE | I_original - PLINK | 19 | 1.046 | 0.929 | 1.179 | 0.045 | 0.061 | 4.55e-01 | - | - | - | - | - |
| MR-PRESSO | I_original - PLINK | 19 | 1.019 | 0.971 | 1.068 | 0.018 | 0.024 | 4.59e-01 | - | - | - | - | 0 |

^*^ MR analyses conducted using asthma/IgE as exposures and myopia binary trait (PLINK/SAIGE) as outcomes. Summary statistics of asthma/IgE were obtained from IEU OpenGWAS Project (code: ebi-a-GCST90086044 and prot-a-1456, repectively) to match the source used in the original MR study.

a_original/I_original: MR conducted using IVs of asthma/IgE as independent clumps using the clumping criteria reported in the original paper (p < 5e-06, r^2^ < 0.01, 1000kb);

a_new/I_new: MR conducted using IVs of asthma/IgE as independent clumps using stricter clumping criteria (p < 5e-08, r^2^ < 0.01, 1000kb)

# Supplementary Table S10. Full MR results for re-analyses examining the effect of *Propionibacterium Freudenreichii (P. freudenreichii)* on myopia.

| Method | Sumstats^*^ | Num_IVs | OR | LCI95 | UCI95 | BETA | SE | P | Qstat | Qpval | Egger_intercept | Egger_P | MRP_outliers |
| --- | --- | --- | --- | --- | --- | --- | --- | --- | --- | --- | --- | --- | --- |
| Wald Ratio | *P. freudenreichii -* PLINK | 1 | 0.782 | 0.572 | 1.069 | -0.246 | 0.160 | 1.24e-01 | - | - | - | - | - |
| Wald Ratio | *P. freudenreichii -* SAIGE | 1 | 0.845 | 0.627 | 1.139 | -0.168 | 0.152 | 2.70e-01 | - | - | - | - | - |

^*^ MR analyses conducted using *Propionibacterium Freudenreichii* as exposure and myopia binary trait (PLINK/SAIGE) as outcome.

Summary statistics of *Propionibacterium Freudenreichii* were obtained from the IEU OpenGWAS Project (code: GCST90032525) to match the source used in the original MR study.

# Supplementary Table S11. Full MR results for re-analyses examining the effect of anxiety and depression on myopia.

| Method | Sumstats^*^ | Num_IVs | OR | LCI95 | UCI95 | BETA | SE | P | Qstat | Qpval | Egger_intercept | Egger_P | MRP_outliers |
| --- | --- | --- | --- | --- | --- | --- | --- | --- | --- | --- | --- | --- | --- |
| Wald Ratio | anxiety - PLINK | 1 | 0.901 | 0.773 | 1.050 | -0.104 | 0.078 | 1.83e-01 | - | - | - | - | - |
| Wald Ratio | anxiety - SAIGE | 1 | 0.950 | 0.821 | 1.098 | -0.052 | 0.074 | 4.86e-01 | - | - | - | - | - |
| Wald Ratio | anxiety - MRCIEU_transformed^†^ | 1 | 1.071 | 0.967 | 1.187 | 0.069 | 0.052 | 1.90e-01 | - | - | - | - | - |
| IVW-MR | depression - SAIGE | 18 | 0.910 | 0.781 | 1.061 | -0.094 | 0.078 | 2.28e-01 | 26.889 | 0.060 | - | - | - |
| MR-EGGER | depression - SAIGE | 18 | 1.126 | 0.413 | 3.072 | 0.118 | 0.512 | 8.17e-01 | 26.596 | 0.046 | -0.010 | 6.75e-01 | - |
| MR-WMEDIAN | depression - SAIGE | 18 | 1.012 | 0.847 | 1.209 | 0.012 | 0.091 | 8.95e-01 | - | - | - | - | - |
| MR-MBE | depression - SAIGE | 18 | 1.095 | 0.808 | 1.484 | 0.091 | 0.155 | 5.60e-01 | - | - | - | - | - |
| MR-PRESSO | depression - SAIGE | 17 | 0.965 | 0.860 | 1.083 | -0.036 | 0.059 | 5.54e-01 | - | - | - | - | 1 |
| IVW-MR | depression - PLINK | 19 | 0.974 | 0.820 | 1.158 | -0.026 | 0.088 | 7.69e-01 | 34.834 | 0.010 | - | - | - |
| MR-EGGER | depression - PLINK | 19 | 1.511 | 0.529 | 4.320 | 0.413 | 0.536 | 4.41e-01 | 33.478 | 0.010 | -0.021 | 4.07e-01 | - |
| MR-WMEDIAN | depression - PLINK | 19 | 1.007 | 0.831 | 1.220 | 0.007 | 0.098 | 9.45e-01 | - | - | - | - | - |
| MR-MBE | depression - PLINK | 19 | 1.121 | 0.767 | 1.638 | 0.114 | 0.193 | 5.55e-01 | - | - | - | - | - |
| MR-PRESSO | depression - PLINK | 18 | 1.027 | 0.882 | 1.197 | 0.027 | 0.078 | 7.33e-01 | - | - | - | - | 1 |
| IVW-MR | depression - MRCIEU_transformed^†^ | 18 | 0.952 | 0.824 | 1.101 | -0.049 | 0.074 | 5.09e-01 | 49.981 | 0 | - | - | - |
| MR-EGGER | depression - MRCIEU_transformed^†^ | 18 | 0.977 | 0.374 | 2.554 | -0.023 | 0.490 | 9.62e-01 | 49.973 | 0 | -0.001 | 9.58e-01 | - |
| MR-WMEDIAN | depression - MRCIEU_transformed^†^ | 18 | 1.014 | 0.880 | 1.168 | 0.014 | 0.072 | 8.49e-01 | - | - | - | - | - |
| MR-MBE | depression - MRCIEU_transformed^†^ | 18 | 1.041 | 0.795 | 1.365 | 0.041 | 0.138 | 7.68e-01 | - | - | - | - | - |
| MR-PRESSO | depression - MRCIEU_transformed^†^ | 16 | 0.965 | 0.859 | 1.083 | -0.036 | 0.059 | 5.51e-01 | - | - | - | - | 2 |

^*^MR analyses were conducted using the binary traits anxiety/depression as exposures and the binary trait myopia (PLINK/SAIGE) as outcome.

Summary statistics of depression were obtained from FinnGen R10, matching the source used in the original MR study.

Summary statistics of anxiety were obtained from the Psychiatric Genomics Consortium (PGC) to match the source used in the original MR study.

^†^ MRC IEU_transformed refers to the transformed version of ukb-b-6353 myopia GWAS summary statistics from IEU Open GWAS (effect size of each genetic variant in the ukb-b-6353 summary statistics file was transformed from an absolute risk difference scale to a log odds ratio scale according to the MRC IEU UK Biobank GWAS pipeline, version 2, 18/01/2019)

# Supplementary Table S12. Full MR results for re-analyses examining the effect of adiponectin and HbA1c on myopia.

| Method | Sumstats | Num_IVs | OR | LCI95 | UCI95 | BETA | SE | P | Qstat | Qpval | Egger_intercept | Egger_P | MRP_outliers |
| --- | --- | --- | --- | --- | --- | --- | --- | --- | --- | --- | --- | --- | --- |
| IVW-MR | adiponectin - SAIGE | 13 | 0.979 | 0.849 | 1.129 | -0.021 | 0.073 | 7.72e-01 | 16.381 | 0.174 | - | - | - |
| MR-EGGER | adiponectin - SAIGE | 13 | 0.913 | 0.722 | 1.154 | -0.091 | 0.119 | 4.45e-01 | 15.586 | 0.157 | 0.005 | 4.54e-01 | - |
| MR-WMEDIAN | adiponectin - SAIGE | 13 | 0.953 | 0.809 | 1.122 | -0.048 | 0.083 | 5.63e-01 | - | - | - | - | - |
| MR-MBE | adiponectin - SAIGE | 13 | 0.950 | 0.813 | 1.110 | -0.051 | 0.079 | 5.20e-01 | - | - | - | - | - |
| MR-PRESSO | adiponectin - SAIGE | 13 | 0.979 | 0.849 | 1.129 | -0.021 | 0.073 | 7.77e-01 | - | - | - | - | 0 |
| IVW-MR | adiponectin - PLINK | 12 | 0.949 | 0.784 | 1.150 | -0.052 | 0.098 | 5.94e-01 | 25.902 | 0.700 | - | - | - |
| MR-EGGER | adiponectin - PLINK | 12 | 0.845 | 0.619 | 1.153 | -0.168 | 0.159 | 2.88e-01 | 23.821 | 0.800 | 0.009 | 3.50e-01 | - |
| MR-WMEDIAN | adiponectin - PLINK | 12 | 0.905 | 0.760 | 1.077 | -0.100 | 0.089 | 2.59e-01 | - | - | - | - | - |
| MR-MBE | adiponectin - PLINK | 12 | 0.892 | 0.762 | 1.043 | -0.115 | 0.080 | 1.53e-01 | - | - | - | - | - |
| MR-PRESSO | adiponectin - PLINK | 10 | 0.895 | 0.784 | 1.023 | -0.110 | 0.068 | 1.38e-01 | - | - | - | - | 2 |
| IVW-MR | adiponectin - MRCIEU_transformed^†^ | 12 | 0.907 | 0.834 | 0.986 | -0.098 | 0.043 | 2.21e-02 | 9.712 | 0.556 | - | - | - |
| MR-EGGER | adiponectin - MRCIEU_transformed^†^ | 12 | 0.862 | 0.753 | 0.987 | -0.148 | 0.069 | 3.12e-02 | 8.837 | 0.548 | 0.004 | 3.50e-01 | - |
| MR-WMEDIAN | adiponectin - MRCIEU_transformed^†^ | 12 | 0.895 | 0.796 | 1.006 | -0.111 | 0.060 | 6.28e-02 | - | - | - | - | - |
| MR-MBE | adiponectin - MRCIEU_transformed^†^ | 12 | 0.887 | 0.792 | 0.993 | -0.120 | 0.058 | 3.74e-02 | - | - | - | - | - |
| MR-PRESSO | adiponectin - MRCIEU_transformed^†^ | 12 | 0.907 | 0.838 | 0.981 | -0.098 | 0.040 | 3.30e-02 | - | - | - | - | 0 |
| IVW-MR | HbA1c - SAIGE | 10 | 0.995 | 0.792 | 1.249 | -0.005 | 0.116 | 9.64e-01 | 10.030 | 0.348 | - | - | - |
| MR-EGGER | HbA1c - SAIGE | 10 | 0.810 | 0.474 | 1.383 | -0.211 | 0.273 | 4.39e-01 | 9.224 | 0.324 | 0.009 | 4.03e-01 | - |
| MR-WMEDIAN | HbA1c - SAIGE | 10 | 0.920 | 0.692 | 1.223 | -0.083 | 0.145 | 5.66e-01 | - | - | - | - | - |
| MR-MBE | HbA1c - SAIGE | 10 | 0.894 | 0.647 | 1.234 | -0.112 | 0.164 | 4.95e-01 | - | - | - | - | - |
| MR-PRESSO | HbA1c - SAIGE | 10 | 0.995 | 0.792 | 1.249 | -0.005 | 0.116 | 9.65e-01 | - | - | - | - | 0 |
| IVW-MR | HbA1c - PLINK | 10 | 1.048 | 0.804 | 1.366 | 0.047 | 0.135 | 7.27e-01 | 12.840 | 0.170 | - | - | - |
| MR-EGGER | HbA1c - PLINK | 10 | 1.054 | 0.550 | 2.020 | 0.053 | 0.332 | 8.73e-01 | 12.839 | 0.118 | 0 | 9.84e-01 | - |
| MR-WMEDIAN | HbA1c - PLINK | 10 | 1.028 | 0.764 | 1.384 | 0.028 | 0.151 | 8.53e-01 | - | - | - | - | - |
| MR-MBE | HbA1c - PLINK | 10 | 1.018 | 0.729 | 1.423 | 0.018 | 0.171 | 9.16e-01 | - | - | - | - | - |
| MR-PRESSO | HbA1c - PLINK | 10 | 1.048 | 0.804 | 1.366 | 0.047 | 0.135 | 7.35e-01 | - | - | - | - | 0 |
| IVW-MR | HbA1c - MRCIEU_transformed^†^ | 10 | 1.130 | 0.947 | 1.350 | 0.122 | 0.091 | 1.76e-01 | 12.864 | 0.169 | - | - | - |
| MR-EGGER | HbA1c - MRCIEU_transformed^†^ | 10 | 1.024 | 0.667 | 1.573 | 0.024 | 0.219 | 9.14e-01 | 12.474 | 0.131 | 0.004 | 6.17e-01 | - |
| MR-WMEDIAN | HbA1c - MRCIEU_transformed^†^ | 10 | 1.238 | 1.009 | 1.518 | 0.213 | 0.104 | 4.08e-02 | - | - | - | - | - |
| MR-MBE | HbA1c - MRCIEU_transformed^†^ | 10 | 1.144 | 0.897 | 1.458 | 0.134 | 0.124 | 2.78e-01 | - | - | - | - | - |
| MR-PRESSO | HbA1c - MRCIEU_transformed^†^ | 10 | 1.130 | 0.947 | 1.350 | 0.122 | 0.091 | 2.09e-01 | - | - | - | - | 0 |

^*^MR analyses were conducted using the continuous traits adiponectin/HbA1c as exposures and the binary trait myopia (PLINK/SAIGE) as outcome.

Summary statistics of adiponectin/HbA1c were obtained from the IEU OpenGWAS Project (code: ieu-a-1 and ieu-b-104, respectively) to match the source used in the original MR study.

^†^ MRC IEU_transformed refers to the transformed version of ukb-b-6353 myopia GWAS summary statistics from IEU Open GWAS (effect size of each genetic variant in the ukb-b-6353 summary statistics file was transformed from an absolute risk difference scale to a log odds ratio scale according to the MRC IEU UK Biobank GWAS pipeline, version 2, 18/01/2019)

Figure S1. Graphs of SNP vs. EduYears and SNP vs. Myopia regression coefficients for the inverse variance-weighted Mendelian randomization analyses using different sets of myopia summary statistics. The blue line is the inverse-variance weighted random effects model fit. Error bars indicate 95% confidence intervals.


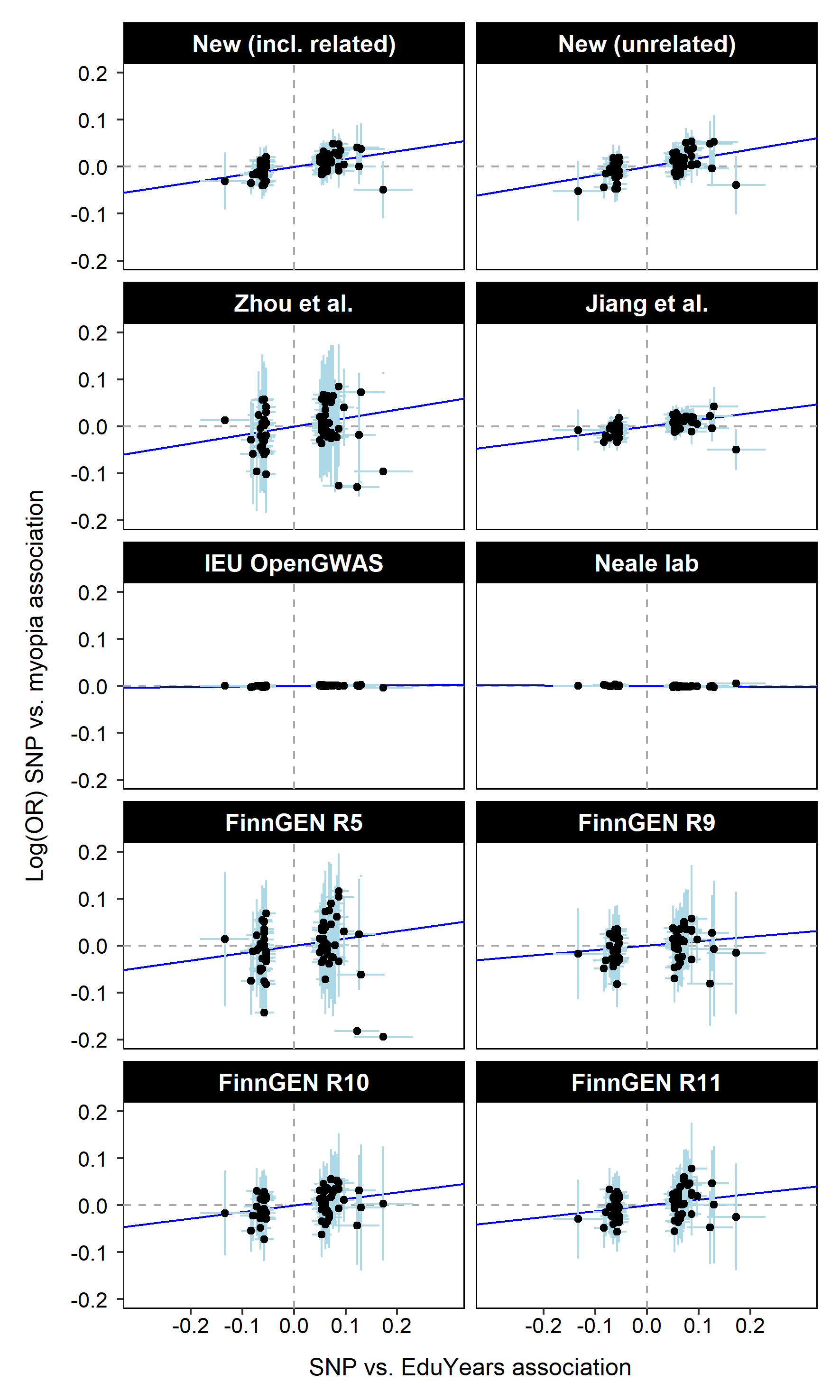


Figure S2. Graphs of SNP vs. Myopia and SNP vs. POAG regression coefficients for the inverse variance-weighted Mendelian randomization analyses using different sets of myopia summary statistics. The blue line is the inverse-variance weighted random effects model fit. Error bars indicate 95% confidence intervals. The number of SNPs (instrumental variables) varies for each set of summary statistics, depending on the number of independent, genome-wide significant SNPs.


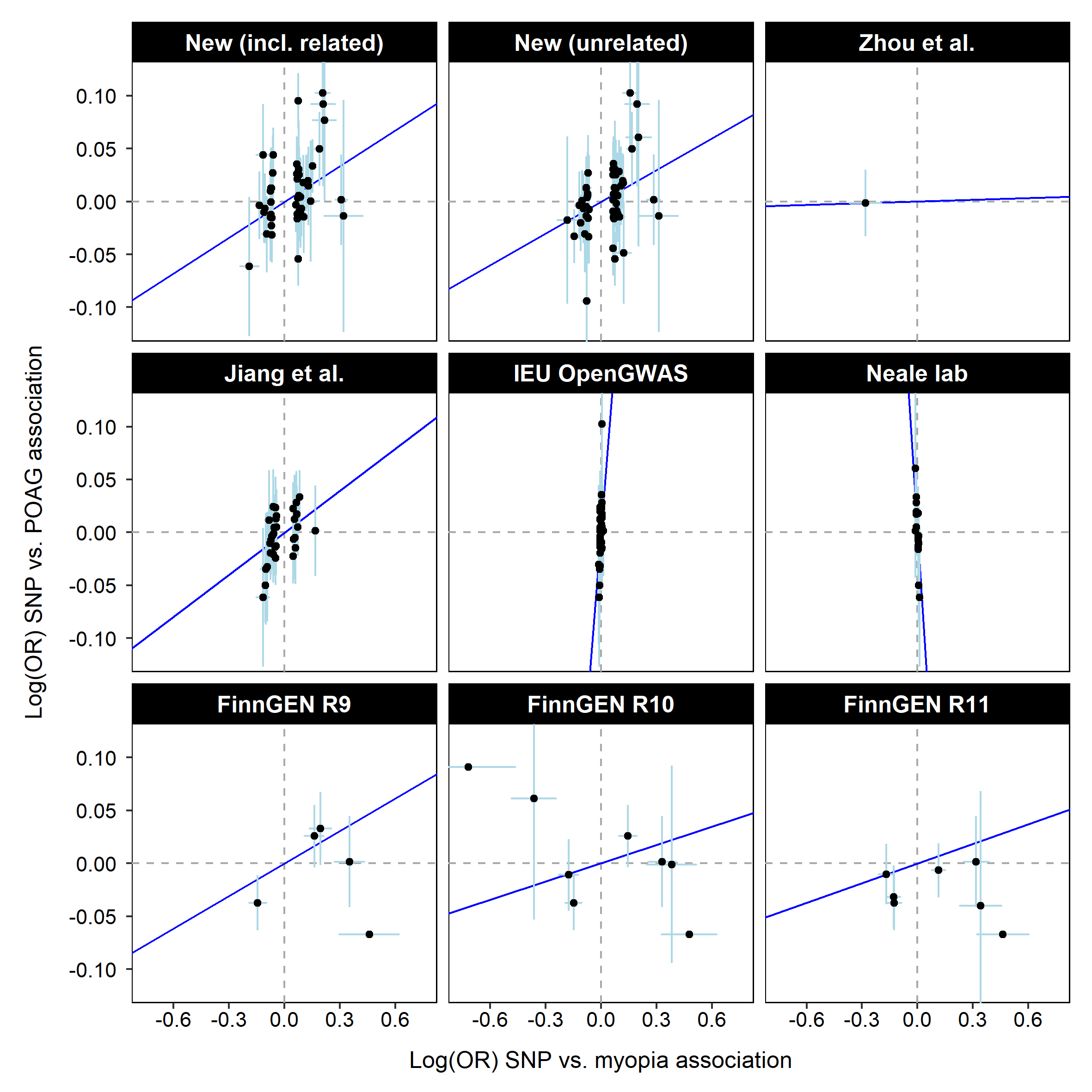


Figure S3. The impact of misinterpretation of GWAS measurement scale on the SNPs used for the EduYears-Myopia MR analysis. The graphs display SNP effect sizes (beta coefficients from GWAS summary statistics) for the 62 SNPs included in the EduYears-Myopia exemplar MR analyses. The blue line depicts the linear association; error bars show 95% confidence intervals. The two upper panels show data for SNPs from the IEU OpenGWAS GWAS summary statistics vs. those from Jiang et al.^2^ The graphs in the two lower panels show data from the Neale lab GWAS summary statistics vs. those from Jiang et al.^2^ The two panels on the left, labeled 'Original', show the untransformed IEU/Neale lab SNP effect sizes (linear measurement scale). The two panels on the right, labeled 'Transformed', display the transformed SNP effect sizes (logOR scale) for the IEU/Neale lab summary statistics.


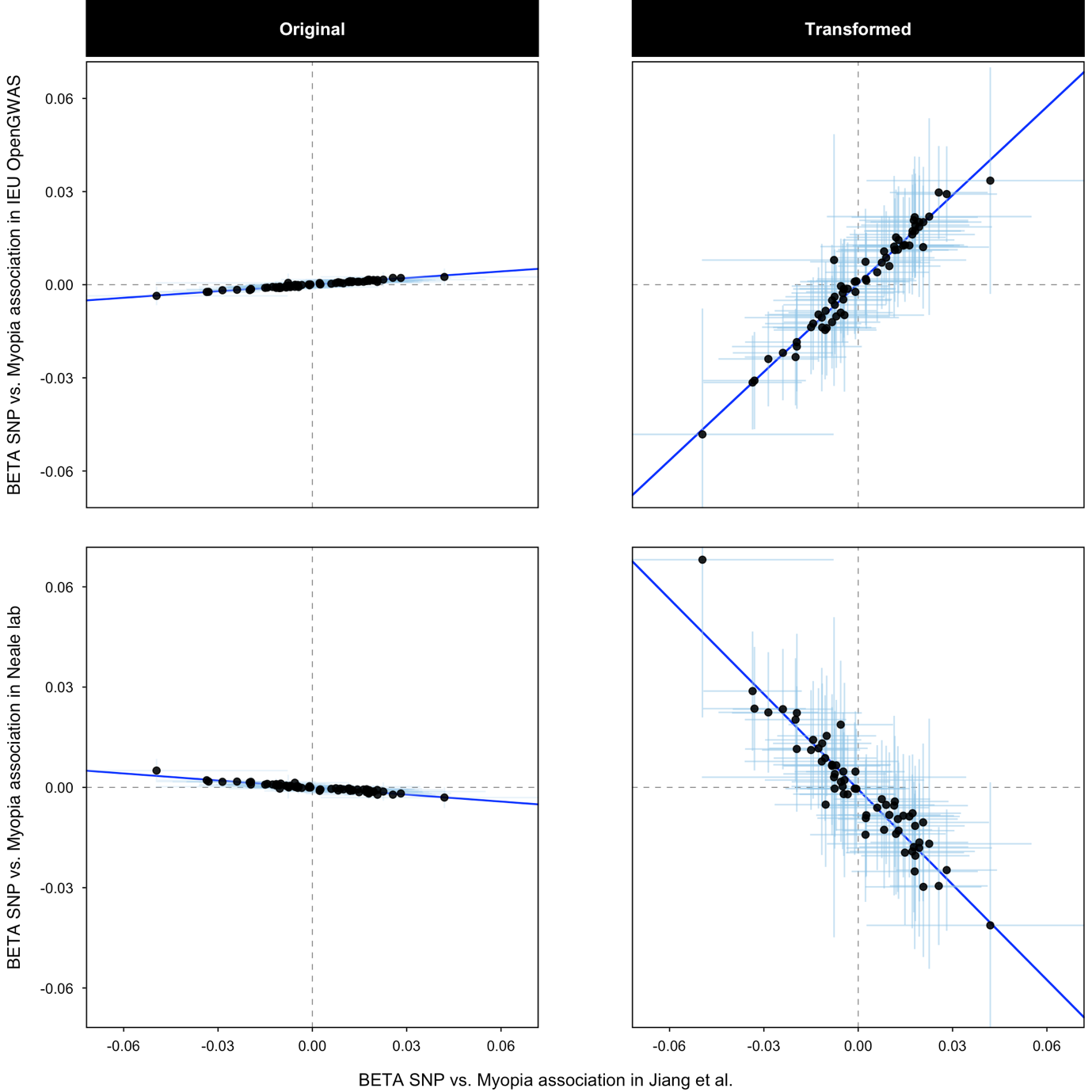


Figure S4. The impact of misinterpretation of GWAS measurement scale on the SNPs used for the Myopia-POAG MR analysis. The graphs display SNP effect sizes (beta coefficients from GWAS summary statistics) for the SNPs included in the Myopia-POAG exemplar MR analyses. The blue line depicts the linear association; error bars show 95% confidence intervals. The two upper panels show data for SNPs (n=18) from the IEU OpenGWAS GWAS summary statistics vs. those from Jiang et al.^2^ The graphs in the two lower panels show data for SNPs (n=12) from the Neale lab GWAS summary statistics vs. those from Jiang et al.^2^ The two panels on the left, labeled 'Original', show the untransformed IEU/Neale lab SNP effect sizes (linear measurement scale). The two panels on the right, labeled 'Transformed', display the transformed SNP effect sizes (logOR scale) for the IEU/Neale lab summary statistics.


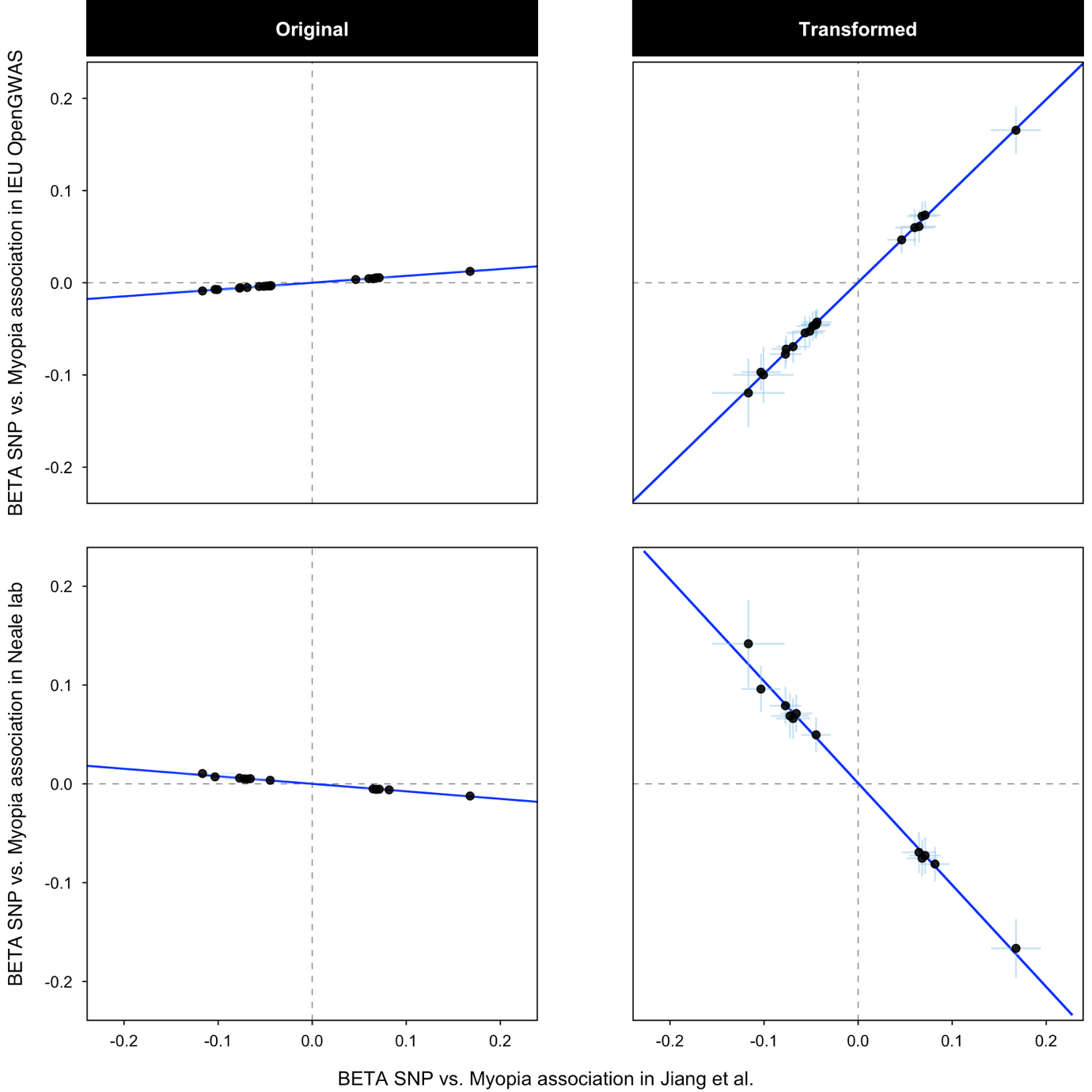


# References

1. Zhou W, Nielsen JB, Fritsche LG, et al. Efficiently controlling for case-control imbalance and sample relatedness in large-scale genetic association studies. *Nat Genet* 2018;50:1335-1341.

2. Jiang L, Zheng Z, Fang H, Yang J. A generalized linear mixed model association tool for biobank-scale data. *Nat Genet* 2021;53:1616-1621.

3. Elsworth BL, Mitchell R, Raistrick CA, Paternoster L, Hemani G, Gaunt TR. MRC IEU UK Biobank GWAS pipeline version 1. 2017:doi: 10.5523/bris.5522fahpksont5521zi5526xosyamqo5528rr.

4. Kurki MI, Karjalainen J, Palta P, et al. FinnGen provides genetic insights from a well-phenotyped isolated population. *Nature* 2023;613:508-518.

5. Gharahkhani P, Jorgenson E, Hysi P, et al. Genome-wide meta-analysis identifies 127 open-angle glaucoma loci with consistent effect across ancestries. *Nat Commun* 2021;12:1258.

6. Chang CC, Chow CC, Tellier LC, Vattikuti S, Purcell SM, Lee JJ. Second-generation PLINK: rising to the challenge of larger and richer datasets. *GigaScience* 2015;4:7.

7. Lee SH, Goddard ME, Wray NR, Visscher PM. A Better Coefficient of Determination for Genetic Profile Analysis. *Genet Epidemiol* 2012;36:214-224.

8. Okbay A, Beauchamp JP, Fontana MA, et al. Genome-wide association study identifies 74 loci associated with educational attainment. *Nature* 2016;533:539–542.

9. Burgess S, Small DS, Thompson SG. A review of instrumental variable estimators for Mendelian randomization. *Stat Methods Med Res* 2017;26:2333-2355.

10. Patel A, Ye T, Xue H, et al. MendelianRandomization v0.9.0: updates to an R package for performing Mendelian randomization analyses using summarized data. *Wellcome Open Res* 2023;8:449.

11. Verbanck M, Chen C-Y, Neale B, Do R. Detection of widespread horizontal pleiotropy in causal relationships inferred from Mendelian randomization between complex traits and diseases. *Nat Genet* 2018;50:693-698.

12. Myers TA, Chanock SJ, Machiela MJ. LDlinkR: An R Package for Rapidly Calculating Linkage Disequilibrium Statistics in Diverse Populations. *Frontiers in Genetics* 2020;11:157.

13. Li X, Luo S, Lin K, et al. Causal Links Between Corneal Biomechanics and Myopia: Evidence from Bidirectional Mendelian Randomization in the UK Biobank. *Bioengineering*; 2025:doi:10.3390/bioengineering12040412.

14. Han X, Ong J-S, An J, et al. Association of Myopia and Intraocular Pressure With Retinal Detachment in European Descent Participants of the UK Biobank Cohort: A Mendelian Randomization Study. *JAMA Ophthalmol* 2020;138:671-678.

15. Wei Zhang S, Guo J, Chen Y, et al. Associations Between Myopia and Brain Volumes: An Observational and Genetic Analysis. *Invest Ophthalmol Vis Sci* 2025;66:57.

16. Lin B, Chen LL, Li DK. Mendelian randomization analysis reveals a causal relationship between preterm birth and myopia risk. *Front Pediatr* 2024;12:1404184.

17. Deng B, Zhou M, Kong X, et al. The lack of causal link between myopia and intraocular pressure: Insights from cross-sectional analysis and Mendelian randomization study. *Photodiagnosis Photodyn Ther* 2024;49:104334.

18. Xu X, Liu N, Yu W. No Evidence of an Association between Genetic Factors Affecting Response to Vitamin A Supplementation and Myopia: A Mendelian Randomization Study and Meta-Analysis. *Nutrients* 2024;16:doi: 10.3390/nu16121933.

19. Dong XX, Xie JY, Li DL, et al. Association of sleep traits with myopia in children and adolescents: A meta-analysis and Mendelian randomization study. *Prev Med* 2024;180:107893.

20. Liu X, Zhao F, Yuan W, Xu J. Causal relationships between height, screen time, physical activity, sleep and myopia: univariable and multivariable Mendelian randomization. *Front Public Health* 2024;12:1383449.

21. Zhang XB, Jiang HH, Zhang LL, et al. Potential causal associations between leisure sedentary behaviors, physical activity, sleep traits, and myopia: a Mendelian randomization study. *BMC Ophthalmol* 2024;24:104.

22. Su M-R, Zou X, Xie H-R, et al. The causal effect of multiple lifestyles and myopia: a Mendelian randomization study. *Sci Rep* 2025;15:13468.

23. Zhu G, Tian R, Zhou D, Qin X. Genetic correlation and causal relationship between sleep and myopia: a mendelian randomization study. *Front Genet* 2024;15:1378802.

24. Xia T, Nakayama K. Signatures of adaptation in myopia-related genes on the sunlight exposure hypothesis. *J Physiol Anthropol* 2023;42:25.

25. Wei P, Han G, Su Q, Jia L, Xue C, Wang Y. Corneal biomechanics as a causal factor in myopia and astigmatism: Evidence from Mendelian randomization. *Ophthalmol Sci* 2025;100738.

26. Fan S, Shi XY, Li X, Li J, Yu SP. Vitamin D levels and risk of ocular disorders: insights from bidirectional and multivariable Mendelian randomization analysis. *Front Med* 2024;11:1431170.

27. Luo X, Ruan Z, Liu L. Causal effect of the 25-Hydroxyvitamin D concentration on ocular diseases: A Mendelian randomization study. *Sci Rep* 2025;15:8701.

28. Deng Z, Buyang Z, Hou T. Visual impairment and frailty: insight from genetic correlation and Mendelian randomization. *Arch Med Sci* 2025;doi:10.5114/aoms/200789.

29. Xu W, Shi W. Deciphering the Microbiome-Gut-Eye Axis: A Mendelian Randomization Analysis of the Causal Influence of Gut Microbiota on Myopia. *Comb Chem High Throughput Screen* 2025;doi:10.2174/0113862073385717250415110224.

30. Mo Q, Liu X, Gong W, et al. Pinpointing Novel Plasma and Brain Proteins for Common Ocular Diseases: A Comprehensive Cross-Omics Integration Analysis. *Int J Mol Sci*; 2024:doi:10.3390/ijms251910236.

31. Jiang X, Xu B, Li Q, Zhao YE. Association between Plasma Metabolite Levels and Myopia: A 2-Sample Mendelian Randomization Study. *Ophthalmol Sci* 2025;5:100699.

32. Liang R, Li T, Gao H, et al. Causal relationships between inflammatory cytokines and myopia: an analysis of genetic and observational studies. *Ann Med Surg* 2024;86:5179-5190.

33. Lv H, Wang Z, Huang C, Yu X, Li X, Song X. Causal Links between Gut Microbiota, Blood Metabolites, Immune Cells, Inflammatory Proteins, and Myopia: A Mendelian Randomization Study. *Ophthalmol Sci* 2025;5:100684.

34. Huang Z, Zhou J, Liu S, et al. The interplay between systemic inflammation and myopia: A bidirectional Mendelian randomization and experimental validation study. *International Immunopharmacology* 2025;157:114803.

35. Huang Z, Chen J, Shi L, Huang J. Causal associations between smoking and ocular diseases: a Mendelian randomization study. *Adv Ophthalmol Pract Res* 2025;5:220-225.

36. Wei D, Wang H, Huang L, et al. A Mendelian randomization study on the causal relationship between smoking, alcohol consumption, and the development of myopia and astigmatism. *Sci Rep* 2024;14:1868.

37. Fan Y, Wang Z, Wu M, Lin L, Chen L, Zheng B. Bidirectional Causal Relationship Between Myopia and Neurodegenerative Diseases: Two-Sample Mendelian Randomization Analyses. *Br J Hosp Med* 2025;86:1-19.

38. Bai W-Y, Zhang H-W, Ye X-F, Xu J-F, Guo X-J, He J. Association Between Body Mass Index and Myopia: Results from NHANES and Mendelian Randomization. *Ophthalmic Epidemiol* 2025;doi: 10.1080/09286586.09282025.02483684.
